# International variation in neighborhood walkability, transit, and recreation environments using geographic information systems: the IPEN adult study

**DOI:** 10.1186/1476-072X-13-43

**Published:** 2014-10-25

**Authors:** Marc A Adams, Lawrence D Frank, Jasper Schipperijn, Graham Smith, James Chapman, Lars B Christiansen, Neil Coffee, Deborah Salvo, Lorinne du Toit, Jan Dygrýn, Adriano Akira Ferreira Hino, Poh-chin Lai, Suzanne Mavoa, José David Pinzón, Nico Van de Weghe, Ester Cerin, Rachel Davey, Duncan Macfarlane, Neville Owen, James F Sallis

**Affiliations:** Exercise and Wellness Program, School of Nutrition and Health Promotion & Global Institute of Sustainability (GIOS), Arizona State University, 425 N. 5th Street (MC3020), Phoenix, Arizona; Health and Community Design Lab, Schools of Population and Public Health and Community and Regional Planning, University of British Columbia, British Columbia, Canada; Urban Design 4 Health, Inc., Seattle, WA USA; Department of Sports Science and Clinical Biomechanics, University of Southern Denmark, Odense, Denmark; Institute for Environment, Sustainability and Regeneration, Staffordshire University, Stoke-on-Trent, United Kingdom; Spatial Epidemiology and Evaluation Research Group, School of Population Health, Sansom Institute for Health Research, University of South Australia, South Australian Health & Medical Research Institute (SAHMRI), Adelaide, South Australia Australia; Nutrition and Health Sciences Research Center, National Institute of Public Health of Mexico, Cuernavaca, Mexico; Stanford Prevention Research Center, Stanford University School of Medicine, Palo Alto, CA USA; University of Queensland, Brisbane, Australia; Institut of Active Lifestyle, Faculty of Physical Culture, Palacky University, Olomouc, Czech Republic; Department of Physical Education, School of Health and Biosciences, Pontifícia Universidade Católica do Paraná, Curitiba, Parana Brazil; Department of Geography, The University of Hong Kong, Hong Kong, SAR China; SHORE and Whāriki Research Centre, School of Public Health, Massey University, Kragujevac, New Zealand (Mavoa); McCaughey VicHealth Centre for Community Wellbeing, School of Population and Global Health, The University of Melbourne, Melbourne, Australia; Urban Design Department, Fundación Universidad de Bogotá Jorge Tadeo Lozano, Bogotá, Colombia; CartoGIS Research Group, Department of Geography, Faculty of Sciences, Ghent University, Ghent, Belgium; Institute of Human Performance, The University of Hong Kong, Hong Kong, SAR China; Centre for Research and Action in Public Health, University of Canberra, Canberra, Australia; Baker IDI Heart and Diabetes Institute, Melbourne, Australia; Department of Family and Preventive Medicine, University of California, San Diego, USA; Nutrition and Health Sciences Program, Emory University, Atlanta, GA USA

**Keywords:** Walkability, Urban planning, Exercise, Built environment, International health, Transportation, Parks, Physical activity

## Abstract

**Background:**

The World Health Organization recommends strategies to improve urban design, public transportation, and recreation facilities to facilitate physical activity for non-communicable disease prevention for an increasingly urbanized global population. Most evidence supporting environmental associations with physical activity comes from single countries or regions with limited variation in urban form. This paper documents variation in comparable built environment features across countries from diverse regions.

**Methods:**

The International Physical Activity and the Environment Network (IPEN) study of adults aimed to measure the full range of variation in the built environment using geographic information systems (GIS) across 12 countries on 5 continents. Investigators in Australia, Belgium, Brazil, Colombia, the Czech Republic, Denmark, China, Mexico, New Zealand, Spain, the United Kingdom, and the United States followed a common research protocol to develop internationally comparable measures. Using detailed instructions, GIS-based measures included features such as walkability (i.e., residential density, street connectivity, mix of land uses), and access to public transit, parks, and private recreation facilities around each participant’s residential address using 1-km and 500-m street network buffers.

**Results:**

Eleven of 12 countries and 15 cities had objective GIS data on built environment features. We observed a 38-fold difference in median residential densities, a 5-fold difference in median intersection densities and an 18-fold difference in median park densities. Hong Kong had the highest and North Shore, New Zealand had the lowest median walkability index values, representing a difference of 9 standard deviations in GIS-measured walkability.

**Conclusions:**

Results show that comparable measures can be created across a range of cultural settings revealing profound global differences in urban form relevant to physical activity. These measures allow cities to be ranked more precisely than previously possible. The highly variable measures of urban form will be used to explain individuals’ physical activity, sedentary behaviors, body mass index, and other health outcomes on an international basis. Present measures provide the ability to estimate dose–response relationships from projected changes to the built environment that would otherwise be impossible.

**Electronic supplementary material:**

The online version of this article (doi:10.1186/1476-072X-13-43) contains supplementary material, which is available to authorized users.

## Background

The world has experienced an explosive growth in population and transition from rural to urban areas over the last half century [1]. For the first time in history, more than half of the world’s population live in cities, and an estimated 70% of the population is expected to do so by the middle of the 21st century [1, 2]. People in urban areas experience epidemic levels of physical inactivity [3]. Worldwide, an estimated 31% of adults are physically inactive, but the prevalence of inactivity varies greatly between World Health Organization (WHO) regions, ranging from 17% in Southeast Asia to 43% in the Americas [4]. Physical inactivity is a leading cause of preventable morbidity and mortality worldwide, equivalent to smoking for total mortality, and inactivity is a major modifiable risk factor of many non-communicable diseases (NCDs) [5, 6].

National and international public health agencies, such as the US Centers for Disease Control and Prevention and the WHO, have adopted multi-level ecological frameworks to reduce physical inactivity [7]. Ecological models postulate that built environment features, along with social and policy environments, can support or inhibit physical activity. Neighborhood built environment features are expected to have influences that differ by domains of physical activity. Transportation-related physical activity (primarily walking and cycling) has been associated with more compact housing [8], diverse and accessible destinations within walking distance [9–11], and connected street networks [12], which collectively have been summed to a “walkability index” [13–15]; and to the availability of options for public transportation [16–19]. Recreational or leisure physical activity has been associated with access to and qualities of public parks and private recreational facilities [7, 20–22].

Although reviews of the relevant bodies of evidence provide overall support for associations between neighborhood environment features and physical activity, the current evidence is not as strong or consistent as expected [23–25]. In their 2012 “umbrella review” of systematic reviews, Bauman and colleagues found that few environmental correlates have been consistently related to active transport or recreational physical activity [26]. One limitation may be the differences in methods and scales used to measure the built environment. Although the Bauman review did not distinguish between objective and perceived environmental measures, self-reports of environmental features and physical activity dominated the studies reviewed. Environmental features, however, can be measured objectively using systematic audits or Geographic Information Systems (GIS) and may be closer to measuring what actually exists, though studying environmental perceptions also can be informative. Brownson et al. (2009) reviewed more than 50 studies using objective environmental measures and found a large degree of variability in how common built environment constructs were operationalized using GIS [27]. Variation was found in (1) scale ranging from parcels to entire jurisdictions; (2) definitions of variables; (3) GIS procedures such as assigning parcels to buffers; (4) neighborhood buffer sizes (e.g. 800 meters vs. 1600 meters) and buffer types (Euclidean vs. street network); and (5) specific metrics such as the number of occurrences per land area versus distance to nearest type of use. The variation in GIS methods across studies presents a significant barrier to the identification of consistent associations that provide the basis for public health and policy recommendations.

Several other methodological limitations that may contribute to the inconsistent associations have been proposed [25]. Most studies have been conducted in regions within a single country, particularly higher income countries in North America, Australasia, and Europe [28], but with recent studies from South America [29–32] and Asia [33, 34]. A few U.S. studies have examined inter-regional differences at the national level [35, 36], but they used extremely coarse (county) level measures of the built environment and failed to assess where people actually lived, worked, and performed recreation activities. Intra-regional studies are constrained by the limits of built environment features that exist in each region and do not account for the true range and variation of built environment features possible. For example, most U.S. cities are far less dense and compact than European and Asian cities [37]. Thus, studies to date had constrained environmental variance that may suppress or attenuate associations, and single-country studies likely examined different portions of the distribution of environmental features.

One study examined the relation between the built environment and physical activity across countries. The 11-country International Prevalence Study (IPS) included common self-report measures of the built environment and a wide range of environments [38]. Stronger associations between built environment features and physical activity were found among the pooled sample, compared to single-country studies that have limited variability [38–40]. Despite the strengths of the 11-country study, the IPS used a brief 7-item survey of environmental factors, lacked objective measurement of the built environment, and was not designed to maximize environmental variability within and across countries.

In summary, the combined lack of behavior-specific measures of environment features, lack of comparability in the methods and operational definitions of measures across studies, and a focus on intra-regional environments with limited variation in environmental features would logically result in mixed findings and underestimated associations between built environment features and physical activity. An international study that evaluates behavior-specific impacts in a broader international range of contexts could help to provide the evidence needed to document built environment features as a global public health priority for physical activity and other health outcomes. The purpose of the present paper was to (1) document the IPEN Adult Study methodological approach for developing a common GIS protocol used by participating countries for measuring the built environment; (2) describe the availability of GIS data and comparability of measures across countries for key components of neighborhood walkability (residential densities, street connectivity, and land use mix), transit environments (access to bus and rail stops/stations) and recreation environments (access to parks and private recreation facilities); (3) present the range of variation observed across countries on key components of walkability, transit, and recreation environments and, (4) discuss observations, lessons learned, and the next steps for examining built environment relations to physical activity across multiple countries.

## Methods

The International Physical Activity and the Environment Network (IPEN) represents a consortium of physical activity and built environment researchers from a wide range of countries (see http://www.ipenproject.org). The IPEN Adult Study emerged from this network and is an observational cross-sectional study of built environments and physical activity from urbanized regions of 12 diverse countries: Adelaide, Australia (AUS); Ghent, Belgium (BEL); Curitiba, Brazil (BRA); Bogotá, Colombia (COL); Olomouc and Hradec Králové, Czech Republic (CZE); Aarhus, Denmark (DNK); Hong Kong, a Special Administrative Region of China (HKG); Cuernavaca, Mexico (MEX); North Shore, Waitakere, Wellington, and Christchurch, New Zealand (NZL); Pamplona, Spain (ESP); Stoke-on-Trent, United Kingdom (GBR); and Seattle and Baltimore, United States of America (USA)). The IPEN Adult Study aimed to apply a common research design to assure a broad range of built environment features and use comparable objective and self-reported measures of physical activity and the built environment [41]. This design facilitates the robust estimation of dose–response relations between built environment features and physical activity and other outcomes.

The overall IPEN Adult Study design, not including details of GIS methods, has been described previously [41]. Briefly, to increase intra-regional and inter-country comparability and to overcome the limited environmental variability common in many built environment studies, an international study was conducted across 12 countries with lead investigators who had the capacity to conduct such a study in urban and suburban regions in those countries. Countries followed a common methodology with coordination occurring through the IPEN Coordinating Center (IPEN CC). Within each country, a two-stage sampling design was adopted from the Neighborhood Quality of Life Study (NQLS; [42]). First, neighborhoods were purposefully selected to maximize within-city environmental and socioeconomic variation. Second, adult participants were recruited from selected neighborhoods, and built environments around their residential addresses were characterized with the GIS measures reported in the present paper.

### Neighborhood sampling

The IPEN study design required the selection of neighborhoods that met criteria for one of four types defined by high or low walkability crossed with high or low socioeconomic status (SES). Investigators in each country used the smallest *administrative unit* (with a sample approximating between 600 and 1,500 people) with SES data available that roughly represented a neighborhood-level geographic scale within each city for the a priori selection of study neighborhoods. Examples of the units are New Zealand meshblocks, U.S. census block groups, and Hong Kong tertiary planning units. For every administrative unit in a study city or region, a walkability index was derived as a function of at least two of the following variables, based on the methods of Frank et al. [9]: (a) net residential density (ratio of residential units to the land area devoted to residential uses); (b) land use mix (diversity of land use types [spatial entropy]), with normalized scores ranging from 0 to 1 and with 0 being single use and 1 indicating an even distribution of area across several types of uses (e.g., residential, retail, entertainment, institutional); and, (c) connectivity of the street network. In five countries, retail floor area ratio (FAR) was also employed as a proxy for pedestrian-oriented design. SES data (e.g. median household income) was used from a country’s most recent census. The walkability index used for neighborhood selection was not subject to the same level of standardization and quality control that was used for individual buffer-based walkability index reported in the present paper.

Administrative units were ranked based on walkability and SES values. Depending on the country, neighborhood-level “high-walkable” and “low-walkable” were defined by median splits (COL, DNK, MEX) or the top and bottom four deciles of administrative units. “High SES” and “low SES” were defined by median splits or top and bottom four deciles (USA) of administrative units. High and low walkability and SES were then crossed to produce a 2×2 matrix of four quadrants: “high walkable/high SES”, “high walkable/low SES”, “low walkable/high SES”, and “low walkable/low SES”. Kerr et al. provide greater detail on the administrative units and neighborhood sampling used by each country [41].

### Participant sampling

A minimum of 250 residents in each country were systematically sampled and recruited from neighborhoods (i.e. administrative units) that met the definitions of walkability/SES quadrants. Kerr et al. provide further sampling details, expected sample sizes, and explained variations across countries [41]. Each country obtained approval for using human subjects from their local ethics review boards between 2002 and 2011. A total of 12,208 participants provided informed consent prior to data collection, had any GIS data, and were included in the current paper.

### Geographic information system (GIS) measures

Once participants in each country were sampled and consented, investigators created street-network buffers around participants’ homes to estimate unique neighborhood values of various built environment features for each participant using GIS. The concept of “neighborhood” is complex, with several possible definitions and procedures for calculation [43–46]. The IPEN Study chose an individual-level street-network approach because it places participants in the center of their neighborhood and captures what participants can actually access on the road network and removes what they cannot, which has several advantages over administrative boundary approaches to defining neighborhoods. Investigators created measures of walkability, recreation and transportation environment features using these individual-level buffers that were independent of the walkability variables used in the neighborhood selection phase of the study. The present paper reports the methods and findings of the individual-level buffer-based GIS measures, with an emphasis on quality control, standard methods, and creation of comparable variables.

### Formative process for GIS measures

**Figure 1:**
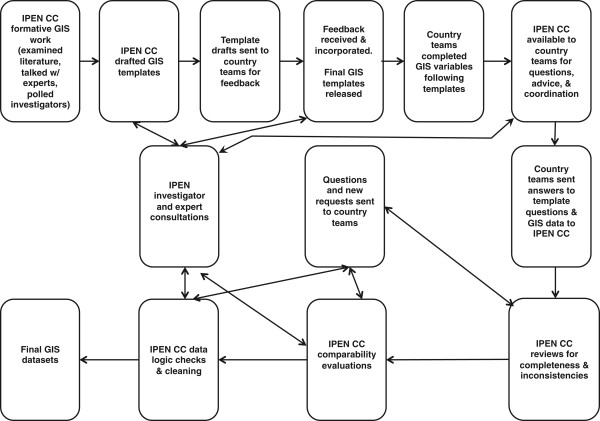
**Flow chart for GIS template creation and IPEN Coordinating Center (IPEN CC) processes.**

Figure 1 presents a flow chart and shows a high-level view of the process for creating and assessing GIS measures. Before the IPEN CC provided guidance on the individual-level GIS component for the study, they reviewed the literature for definitions and procedures for various built environment measures. This process also included discussions with urban planners, a civil engineer, IPEN co-investigators, and GIS analysts in each participating country. This process uncovered meaningful differences in the literature, and early discussions with each country’s GIS team revealed there were important differences in available data, definitions, and GIS procedures used to create variables across countries. For example, residential density could be defined by using the number of people or housing units in the numerator, and street connectivity could be defined as the number of intersections, block length, density of intersections, or link-to-node comparisons [47]. As others have noted, the adoption of standard metrics of connectivity could improve comparisons of results [12].

As a result of these findings, GIS teams in each country were formally polled during this formative process about types of variables that would be possible to create based on available data and technical capacity. Moreover, each analyst reported how he or she planned to calculate the variables. The intent was to identify procedures that would create comparable variables across as many countries as possible.

### GIS templates

Variation in the type and quality of data available within each country mandated the need for an organizing framework to arrive at a common protocol across GIS variables. A “least common dominator” approach was adopted for each GIS variable. In some cases, GIS teams with more detailed datasets for a variable were asked to develop basic versions of variables to be comparable to other countries. In other cases, teams with more basic data were asked to find alternative ways to improve the detail for a specific variable by using another data source, supplementing the dataset, or both. However, countries with more detailed data were also invited to complete more complex versions of variables. The end result was a comparable set of metrics across study regions. The IPEN CC provided common guidance on GIS definitions and procedures across countries and documented several possible choice points for organizing and processing GIS data during variable creation.

The solution was to develop a set of IPEN GIS templates for 11 core built environment constructs considered a priority for IPEN analyses, based on common themes in the physical activity literature. The core GIS templates included: 1) Street Network Buffers, 2) Residential Land Use, 3) Retail Land Use, 4) Civic and Institutional Land Use, 5) Entertainment Land Use, 6) Recreation Land Use, 7) Food-related Land Use, 8) Street Connectivity, 9) Public Transit Access, 10) Private Recreation Facility Access, and 11) Park Access.

Development of the templates was a three-step process. First, the IPEN CC identified important environmental constructs from the physical activity literature, and an expert team drafted each template. The team included two behavioral scientists, an urban and transportation planner, and a civil engineer, all of whom had expertise in measuring the built environment for physical activity. This team developed the set of templates over 6 months with multiple revisions. Second, drafts were sent to investigators and GIS analysts in each country who were asked to provide feedback. Third, countries’ feedback were summarized and presented to the initial drafting team. The team discussed these comments and possible solutions, which were incorporated into the final set of documents.

The 107-page document of IPEN Adult GIS templates aimed to provide GIS teams in each country with specificity for common concepts, clear and consistent definitions, guidance on preferred variables and procedures, and a place to document necessary deviations from the protocol. The 11 core GIS templates correspond to the 11 core constructs listed above and are available for public use [see Additional file 1] [48].

### Data discovery

Countries were instructed to obtain the highest quality spatial data from local sources. The general rule across the countries was to use best available source of information, rather than a similar source of data. Secondary datasets on dwellings and populations were obtained mainly from government censuses (e.g. Colombia’s Unidad Administrativa Especial de Catastro Distal, Denmark’s Central Register of Buildings and Dwellings, Mexico’s National Institute of Statistics and Geography of Mexico, Hong Kong’s Census and Statistics Department, United States’ Census Bureau, New Zealand’s Census, Brazil’s Census, United Kingdom’s Office for National Statistics). Land use data were provided by national or regional repositories (e.g. Planning South Australia, Hong Kong Planning Department, The Institute of Registries and Cadaster of the state of Morelos, Mexico, Maryland Department of Planning and King County GIS Center (USA), Urban Planning Institute of Curitiba (BRA), and United Kingdom’s Ordnance Survey. Private recreation places were obtained from business listings, phone book listings, marketing firm’s address lists, other online internet sources, and parcel data. Park datasets were obtained from multiple sources including field visits, government supplied park lists, GIS shapefiles showing park boundaries, parcel data (indicating park land uses), Google maps, Thomas Guides, internet websites created by various entities, and aerial photography.

### Required and desired variables and procedures

Specific nomenclature was developed for the templates to aid in the process of creating GIS variables. Templates provided guidance on “required, desired, and speculative” GIS variables and procedures. A “required” variable meant that this variable had been judged by IPEN CC staff to be the lowest common denominator (i.e. most likely to be completed) across all countries (e.g., total retail *land area* in a buffer). All countries were asked to produce required variables, if information on that built environment factor was available. A “desired” variable meant that this variable was considered to be of greater importance or higher quality than the required variables (e.g., total retail *floor area* in a buffer), but could be computed by only a subset of participating countries. Desired variables were calculated *in addition* to the required variables. Speculative variables included exploratory measures that only some countries or regions might have available, such as vertical mixing of land uses within parcels.

The IPEN CC provided a guiding hierarchy and nomenclature for GIS procedures. These recommendations for procedures were not enforced as strictly as the definitions because GIS analysts made key decisions that were appropriate given the unique nature of their datasets. “Recommended” procedures were promoted over “acceptable” procedures. Recommended procedures were judged to be more precise methods of calculating the variables. Acceptable procedures were used if recommended procedures could not be used, or if recommended procedures had been deemed inappropriate for country-specific reasons. Speculative procedures were to be used only if recommended or accepted procedures could not be accomplished, but such cases were rare because of feedback obtained from each country during the template development process.

Each country’s GIS team was instructed to adhere closely to the templates. Teams documented their decisions during the variable creation process by answering a series of questions placed at the end of each template for each variable. These questions were designed to ensure a transparent GIS variable creation process and to make explicit specific areas where GIS analysts deviated from IPEN operational definitions during the variable creation process. For example, for each GIS variable we asked whether the GIS analyst adopted the definition provided in the template and whether they deviated from it in any way, either voluntarily or because their dataset attributes were not suitably specific. Because it was possible to use different procedures in GIS to create similar variables, we asked analysts to document their specific procedures (e.g., for calculations of land area, assigning parcels to a participant’s buffer if the parcel centroid fell completely within a buffer vs. if any area of the parcel fell within the buffer). Sometimes these procedural differences were unavoidable because of limitations in attribute information available in each country. Nonetheless, such decisions were documented to make them explicit for the IPEN evaluation of comparability.

### Buffer size and type

Two neighborhood buffers, 500 meters and 1-kilometers (km) in size, were created around each home address for each participant in each city because the optimal buffer size has not been clearly established [45, 46, 49]. Although 11 of 12 countries created street-network buffers, only a subset of countries was able to develop “desired” pedestrian network buffers that included pedestrian sidewalks and informal pathways in addition to the street network. These two buffer types were labeled “street network” and “pedestrian-enhanced street network” buffers, respectively. Thus, all environmental variables were computed in GIS around each individual’s home address for both the street network and, when available, pedestrian-enhanced buffers defined by 500 meter and 1 km.

### Comparability evaluation

Many decision points occur for GIS analysts during the GIS variable creation process. IPEN used a two-step process to ensure quality control of the GIS datasets to make GIS variable creation more comparable and transparent. Each country provided the IPEN CC with their answers to template questions and datasets of computed GIS variables. The IPEN CC performed a preliminary check to ensure completeness and resolve obvious deviations and errors. The IPEN CC provided feedback to the individual countries on template completeness, and if needed, additional requests were made of the GIS staff to clarify the data, definitions, or processes used. Once all countries provided their final templates and GIS data, the IPEN CC initiated a cross-country evaluation of comparability. Two non-independent raters examined template answers across countries for each GIS variable and compared template answers with the definitions of the required and desired variable requests in the templates. The two raters highlighted any deviations in dozens of responses to template questions from one or a subgroup of countries and noted any concerns about comparability. The raters also provided their suggestions to the IPEN CC on how to minimize the comparability concerns. Sometimes, templates were revised to accommodate the capabilities of multiple countries. The goal of this approach was to come to a consensus on comparability and develop strategies to enhance comparability. Thus, inter-rater reliability was not estimated. Their evaluations and solutions were combined and discussed with the IPEN co-investigators to ensure they aligned with other components of the study. Solutions often required that a country or subset of countries recalculate their GIS variables or further clarify their work to ensure comparability across countries.

### Statistical analyses

Once the comparability evaluation was completed, data were cleaned and summarized. Table 1 (see Additional file 2) presents the descriptive values for each built environment variable across cities and for the pooled sample. Figures are used as a visual analysis to highlight the variation both within and across cities for each built environment feature. Box and whisker plots show five pieces of information for each built environment variable: the median, 25th and 75th percentiles, and low and high values represented as 1.5× the height of the interquartile range or minimum or maximum values; 95% of the data are expected to fall between the high and low whiskers. Cities were ranked in figures from lowest to highest by median values. It was not possible to present data for all possible variables in the current paper because dozens of variables were created for each buffer type and size. Therefore, this paper presents data for 1-kilometer (km) street-network buffers only (selected arbitrarily) to demonstrate and document within and between-country variability in built environment features across cities. The data for 500-meter street network buffers are provided as additional files (see Additional file 3 [table] and Additional files 4, 5, 6, 7, 8, 9 and 10 [figures]). All density variables are presented in units per square kilometer (sq km).

## Results

### Geocoding and buffers

Eleven of 12 countries and a total of 15 cities (Pamplona, ESP and Hradec Králové, CZE excluded) had access to spatial datasets and geocoded participants to the level of parcel or street address. An additional exception was Colombia which geocoded participants to the center of the street block. Colombia was not able to obtain precise addresses due to local recruitment circumstances. Eleven countries were able to calculate 500-meter and 1-kilometer street-network buffers using ESRI’s ArcGIS and Network Analyst with the “detailed no trim” setting for buffers. Four countries (i.e., Belgium, the Czech Republic, Denmark, and the United Kingdom) had access to data on sidewalks and/or informal paths and were also able to create 500-m and 1-km pedestrian-enhanced street-network buffers. The comparability evaluation judged that countries closely adhered to the buffer template, and although Colombia geocoded to the center of the block, this imprecision was acceptable given that ESRI algorithms already interpolate specific addresses based on street length.

### Net residential density

**Figure 2:**
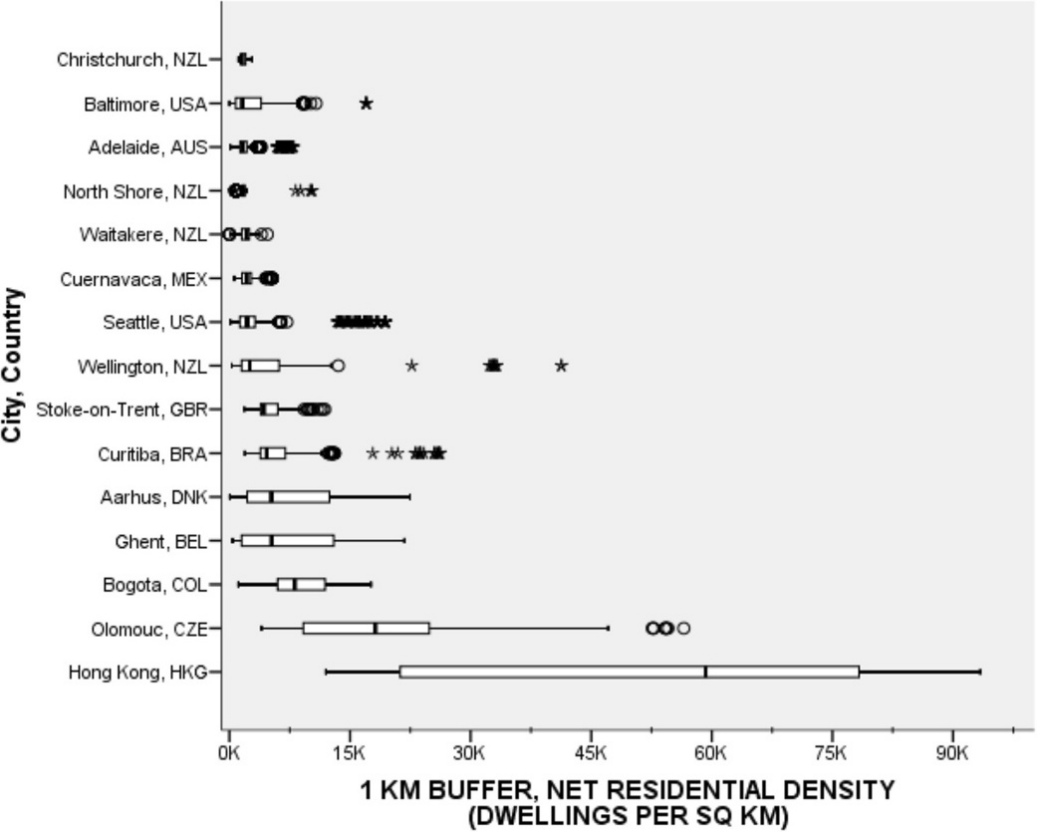
**Net residential density (dwellings per km** ^**2**^ **) for participants’ 1-km network buffers across cities and countries.** Circles are outliers that extend past the whiskers and asterisks represent extreme outliers defined as values greater than three times the length of the interquartile range.

Net residential density was calculated as the number of dwellings (numerator) divided by the land area within participants’ buffers devoted to residential use (denominator). The comparability evaluation revealed that each country, except the Czech Republic, obtained data on dwelling units from their country’s census data, which was used to apportion dwellings to a participant’s buffer. The Czech Republic had access to census population counts and therefore the number of dwelling units in the Czech Republic was estimated by dividing the number of people by the average family size.Figure 2 shows a wide range in median net residential densities between countries, demonstrating an exponentially increasing pattern across the 15 cities. For 1-km buffers, median residential densities ranged from a low of 1,544 dwelling unit/sq km in Christchurch, NZL to a high of 59,247 units/sq km in Hong Kong, HKG. Hong Kong’s lowest density value exceeded the highest density values in Adelaide, AUS, Cuernavaca, MEX, Christchurch, North Shore, and Waitakere NZL, and Stoke-on-Trent, GBR. Hong Kong’s highest density values exceeded 90,000 dwellings/sq km and its within-country variation exceeded that of all the other countries. The pooled median net residential density was 2,504 dwellings/sq km.

### Land use and mixing

**Figure 3:**
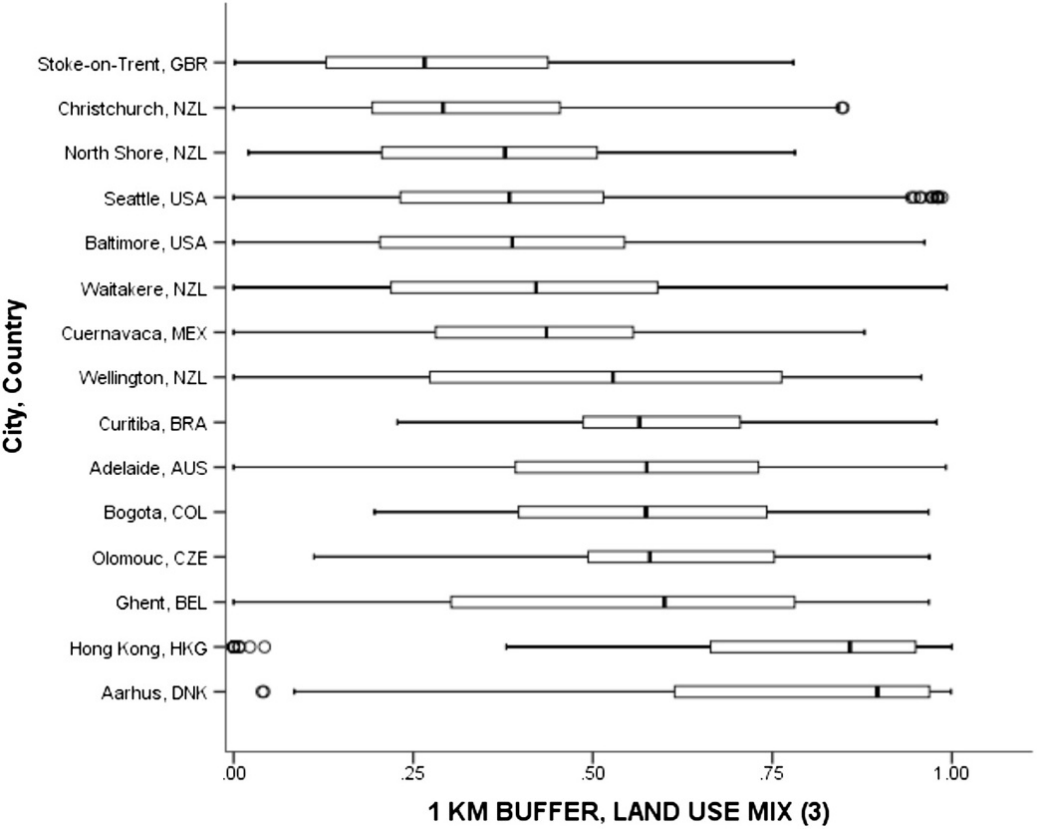
**Land use mix between residential, retail combined, and civic/institutional for participants’ 1-km network buffers across cities and countries.** Circles are outliers that extend past the whiskers and asterisks represent extreme outliers defined as values greater than three times the length of the interquartile range.

IPEN study templates provided guidance on 6 land uses: residential, retail/commercial, civic/institutional, entertainment, food-related, and recreation. During the development process for the templates, measures based on land area were considered possible across all of countries and therefore designated as a required variable, while building floor area measures were designated as a desired variable. The comparability evaluation confirmed that land area measures were possible across 11 countries and 15 cities, building floor areas were available in 3 cities only (Curitiba, BRA, and Baltimore and Seattle, USA), and building footprints were available in Ghent, BEL, only. The evaluation also documented that parcel data were used to quantify land uses in 10 regions, while more crude land cover data was only available in Cuernavaca, MEX. Additionally, the comparability evaluation revealed that the maximum number of comparable land uses across 15 cities and 11 countries was three (i.e. residential, “retail combined”, and institutional/civic). Some countries had coarse classifications, while others had more detailed classifications. For example, the datasets available to Colombia did not separate entertainment and food use from retail/commercial. To resolve this incompatibility, we recoded the data during the analysis phase to arrive at a common retail classification labeled “retail combined” that included retail, food and entertainment land uses for countries with entertainment and food uses separated from retail use. The next largest number of comparable land uses was four across 8 countries (i.e., residential, retail combined, institutional/civic, and recreation). Land use mix was calculated using an entropy equation [50, 51], where values closer to 1.0 indicated an equal distribution of the available residential, retail combined, and institutional/civic land area within the buffer across uses. Values closer to zero indicated a single land use dominated which is typically only the case in residential environments.Figure 3 shows the range of mixed residential, retail combined, and institutional/civic land use mix across 15 cities for 1-km buffers. Many cities had wide within-city ranges of land use mix from a low of zero to a high of near 1.0. Across cities, the lowest median land use mix value was observed in Stoke-on-Trent, GBR (0.27) and the highest median land use mix value was observed in Aarhus, DNK (0.90). Figure 3 shows that median land use values tend to cluster in 4 levels: low (0.27–0.29, i.e., Stoke-on-Trent, GBR and Christchurch, NZL), medium low (0.38–0.44, e.g., North Shore, NZL to Cuernavaca, MEX) medium high (0.53–60, e.g. Wellington, NZL to Ghent, BEL), and high (0.86–0.90, e.g. Hong Kong, HKG and Aarhus, DNK). Notably, the pooled median land use was 0.50.

### Street connectivity

**Figure 4:**
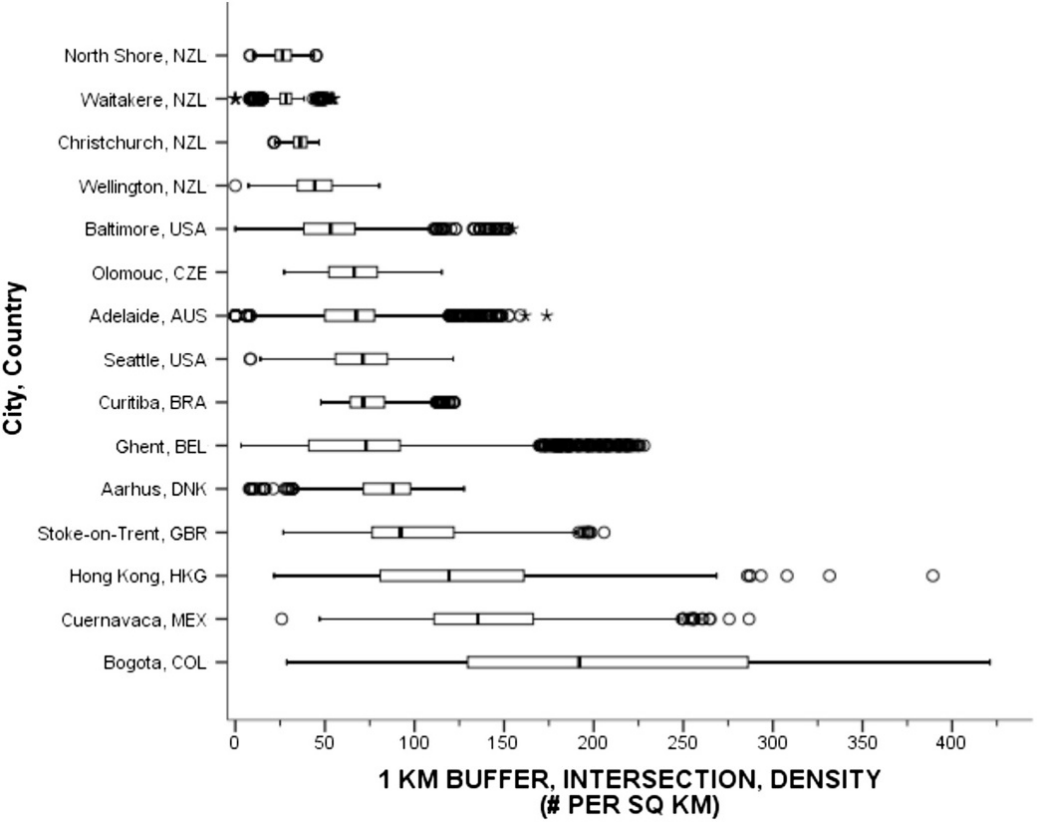
**Intersection densities for participants’ 1-km network buffers across cities and countries.** Circles are outliers that extend past the whiskers and asterisks represent extreme outliers defined as values greater than three times the length of the interquartile range.

Street connectivity was operationalized as intersection density or the ratio of the number of intersections within each participant’s buffer (numerator) divided by the total buffer area. Intersection density is a well-established measure of route directness and captures the ability to traverse between destinations in a direct pathway [52, 12]. An intersection was defined as a point where three or more road segments intersected, after removal of limited access roads and pseudo intersection nodes. Countries were instructed to remove roads where pedestrians were prohibited, such as freeways and freeway on-ramps, before identifying and enumerating intersections. The evaluation revealed that 10 countries removed limited access roads from their analysis. The exception was Colombia, where local circumstances allowed for study participants to reside along what are considered limited access freeways in North America. However, IPEN CC decided after discussion to defer to Colombia’s decision regarding their local circumstance, and the comparability evaluation found that 11 countries computed comparable intersection density variables across 15 cities.Figure 4 shows the variation in intersection densities with the median values demonstrating a cubic-type pattern across cities. The 4 cities in New Zealand represented the lower end of the range, with the lowest median density of intersections across cities (26 per sq km) observed in North Shore, NZL. The highest median was 190 per square km in Bogotá, COL, which also showed the greatest within-city variation, representing a large difference over the pooled median density of 70 intersections per square km. However, because including limited access roads in the intersection density calculation can artificially inflate the number of intersections, many of which were not pedestrian friendly or were completely unwalkable, the value of 135 per square km in Cuernavaca, Mexico was considered the most comparable median value on the high end.

### Walkability

**Figure 5:**
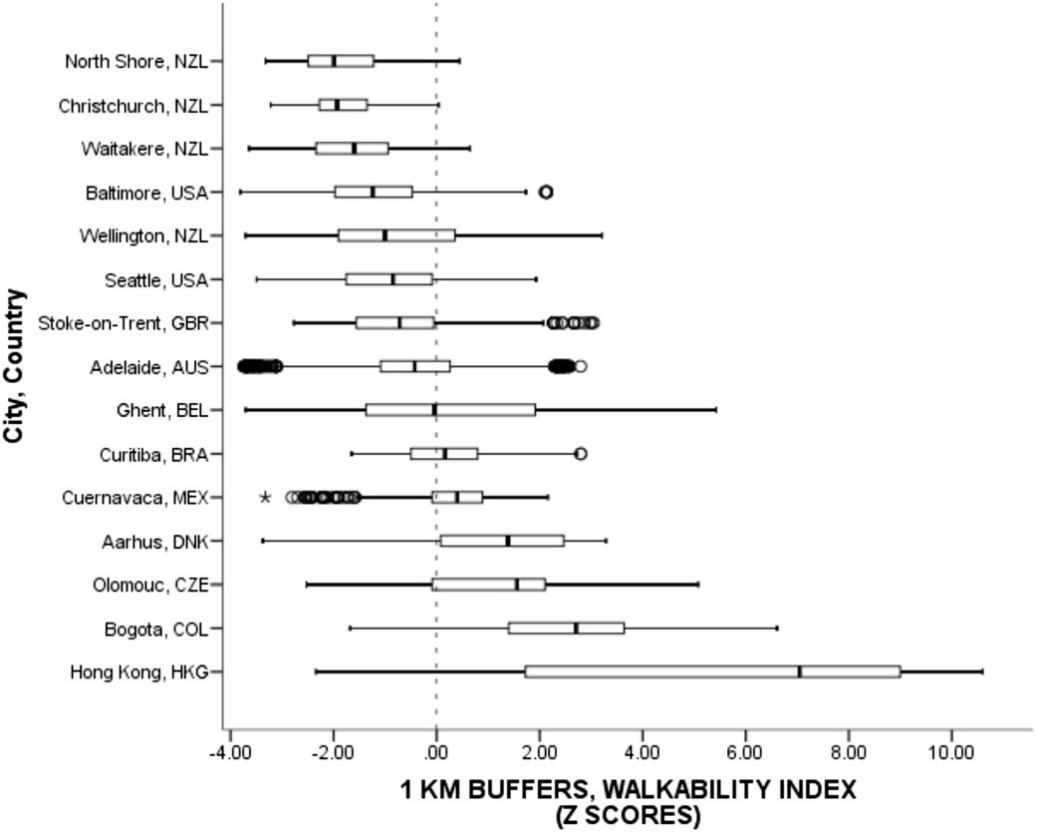
**Walkability scores across cities and countries within participants’ 1-km network buffer.** ^1^Circles are outliers that extend past the whiskers and asterisks represent extreme outliers defined as values greater than three times the length of the interquartile range. ^2^Walkability z-score equaled the sum of z-scores for residential density, land use mix, and intersection density. Z-scores allowed for standardized pooled standard deviations necessary for comparisons across countries.

**Figure 6:**
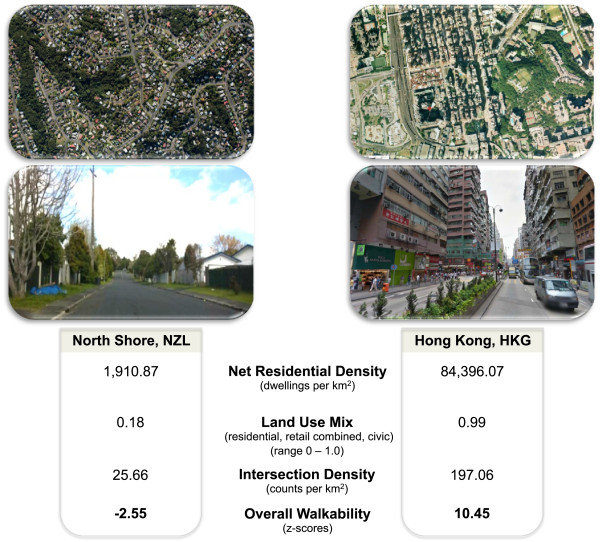
**Aerial and ground views with walkability component and index** ^**1**^ **scores of one of the lowest walkable (North Shore, NZL) and one of the highest walkable (Hong Kong, HKG) neighborhoods in the IPEN Adult Study.** ^1^Walkability index z-score equaled the sum of z-scores for residential density, land use mix, and intersection density. Z-scores allowed for standardized pooled standard deviations necessary for comparisons across countries.

A walkability index was computed as the sum of z-scores (i.e. standardized scores) of net residential density, land use mix, and intersection density. Z-scores were based on pooled unstandardized datasets inclusive of all cities and countries. The walkability index was adapted from Frank et al. [10] but differed from the original index in two ways: (a) land area measures were used instead of floor area measures for land use mix, and (b) “retail floor area ratios” were not included because floor areas were available in only 4 countries. The adapted version, while not the most precise version, still captured both *proximity* and *connectivity* – the two main theoretical constructs of walkability [10]. The IPEN study design maximized within-city variation in net residential density, land use mix, and intersection density and therefore the walkability index values should reflect the maximum range within each city while the comparison across cities should reflect true between-country variation.Figure 5 presents a box plot of the walkability index documenting the within- and between-country variation across 15 IPEN cities. Median walkability z-scores appeared to increase linearly across cities and ranged from a low of -1.99 in North Shore, NZL to a high of 7.05 in Hong Kong, HKG. The pooled median walkability index z-score was -0.37. Importantly, a very large difference of 9.04 standard deviations in median walkability was observed across these 15 cities representing 11 countries and 5 continents. To illustrate the magnitude difference, Figure 6 provides an example of aerial and ground views along with associated walkability and component scores for one of the lowest walkability (North Shore, NZL) and one of the highest walkability (Hong Kong, HKG) neighborhoods in the IPEN Adult Study.

### Public transportation

**Figure 7:**
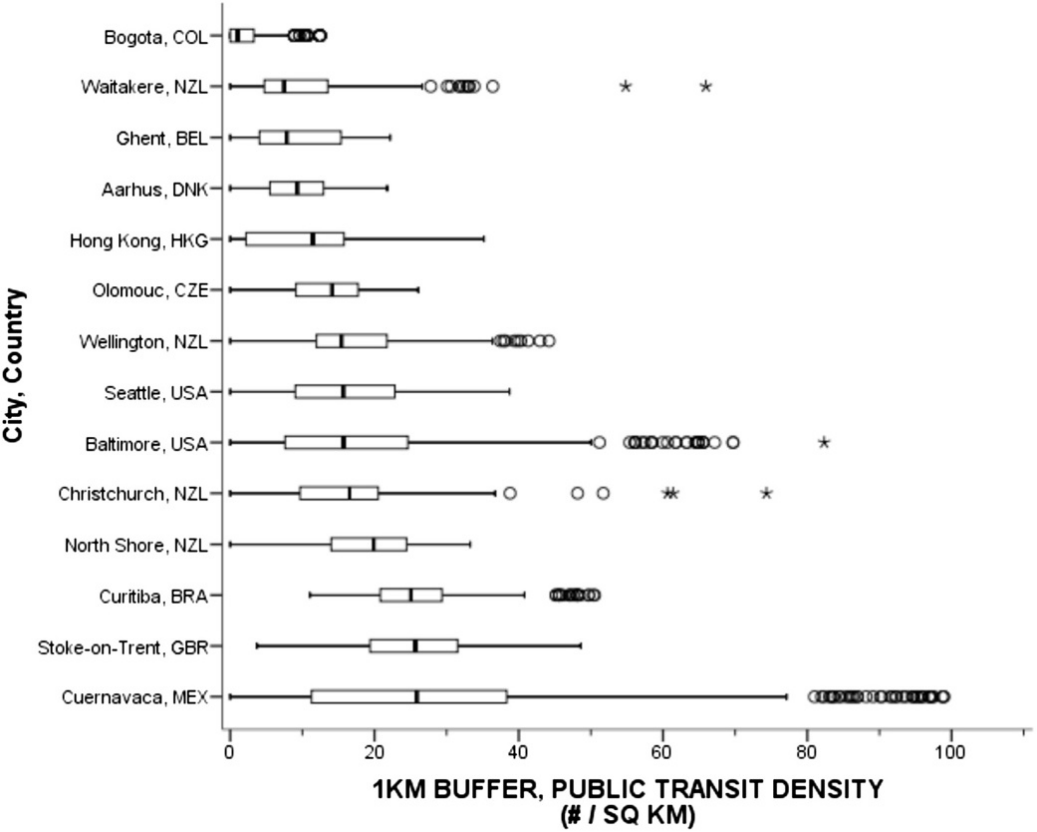
**Public transportation stop density using participants’ 1-km network buffers across cities and countries.** Circles are outliers that extend past the whiskers and asterisks represent extreme outliers defined as values greater than three times the length of the interquartile range.

The public transit template and comparability evaluation determined that the multimodal transit network datasets were complex, and data were limited in some cities and countries. The complexity was reflected by a variety of modes (i.e., bus, rail and ferry) and mode types (e.g., regular bus vs. bus rapid transit, light vs. heavy rail) present within and across cities. The evaluation determined that none of the public transit datasets were available in Adelaide, AUS. Only bus rapid transit stops and stations were available in Bogotá, COL, even though other forms of public transportation existed, such as regular buses. Indeed, Bogotá, COL and Hong Kong, HKG had informal and private bus networks that allow passengers to embark and disembark at any location; hence it was impossible to measure stops. Cuernavaca, MEX had access to regular bus lines only, and bus stops were imputed where bus lines crossed major roads. The remaining countries had comparable public transit datasets. Public transit density was operationalized as the number of public bus, rail, or ferry stops and stations divided by the buffer area.Figure 7 presents the box plot of overall transit density across 14 cities. This figure includes access to any type of public transit network available in the city. Considering the limitations of Bogotá, COL, which had the lowest median transit density at 1.19 stops/sq km when only bus rapid transit was measured, the next lowest median densities were observed in Waitakere, NZL (7.45 stops/sq km) and Ghent, BEL (7.81 stops/sq km). The highest median density was found in Stoke-on-Trent, GBR (25.67 stops/sq km) and Cuernavaca, MEX (25.88 stops/sq km). The pooled median public transit density was 14.17 stops per sq km.

### Parks

**Figure 8:**
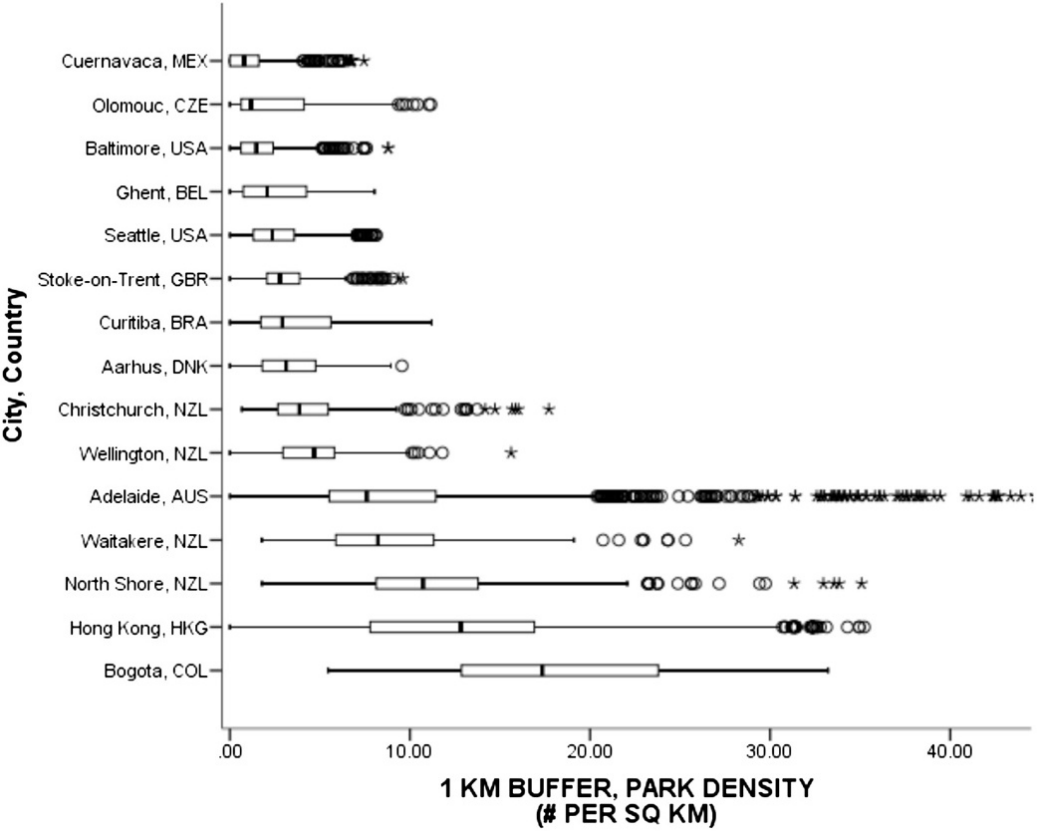
**Density of parks (any size) using participants’ 1-km network buffers across cities and countries.** Circles are outliers that extend past the whiskers and asterisks represent extreme outliers defined as values greater than three times the length of the interquartile range.

Park access was defined as access to a government-designated park of any size that was free and open to the public and maintained by a government agency. Parks included improved and unimproved areas. The comparability evaluation noted that 11 countries were able to identify park datasets for 15 cities; sources varied from government supplied lists to aerial photography. All of the countries were able to quantify the density of parks for six park sizes (i.e., <0.25 acres to >50 acres). The evaluation noted all countries counted park polygons if any portion of the park intersected with participants’ buffers. Park density was computed as the number of parks (numerator) divided by the entire buffer area.Figure 8 shows the median densities of parks (of any size) ranged from a low of 0.79 parks per square km in Cuernavaca, MEX to a high of 17.33 parks per sq km in Bogotá, COL. Median park densities across cities reflected an exponentially increasing type pattern. Within country variation in park densities appeared to increase with higher densities. The pooled median was 4.27 parks per sq km.

### Private recreation facilities

**Figure 9:**
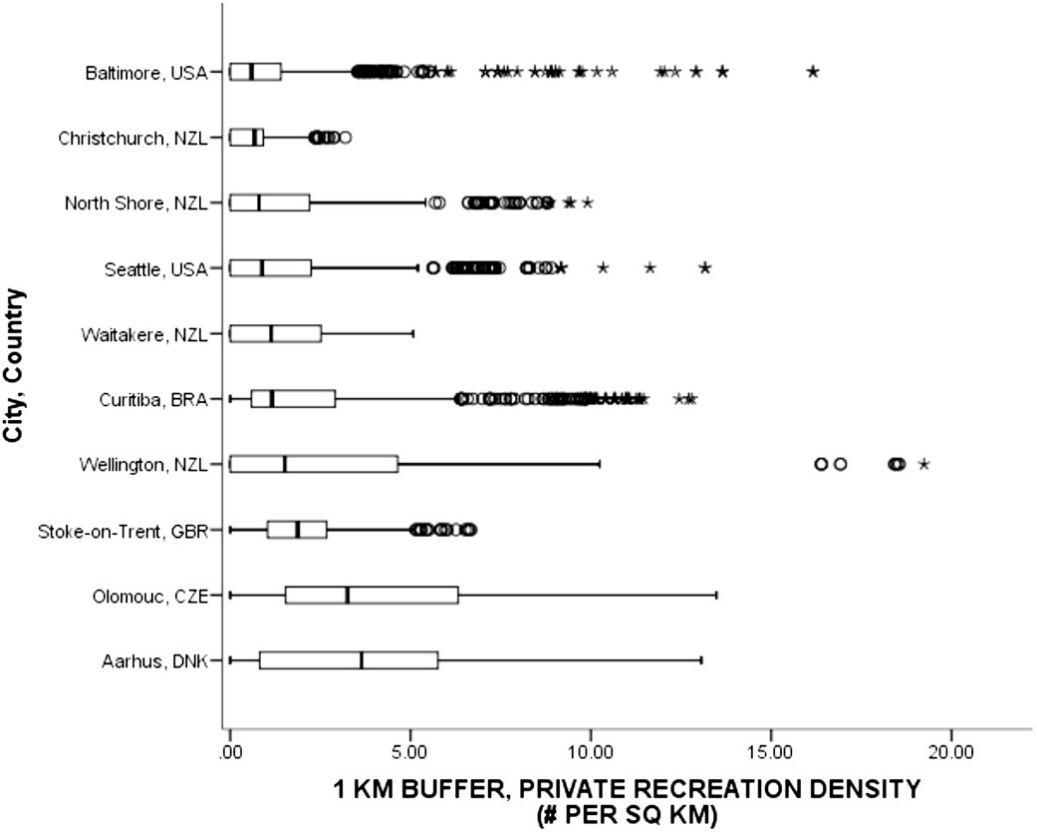
**Density of recreation facilities for participants’ 1-km network buffers across cities and countries.** Circles are outliers that extend past the whiskers and asterisks represent extreme outliers defined as values greater than three times the length of the interquartile range.

The density of private recreation locations was operationalized in the template as indoor and outdoor places where people can be physically active, but the places were not free for public use. Private facilities included fitness centers, health clubs, tennis centers, swimming pools, and golf courses. Public parks were not considered private recreation locations. The comparability evaluation noted that 6 of 11 countries had access to private recreation data covering 10 cities. All adhered to the operational definitions given in the template for private recreation. The evaluation also noted variation in the procedures used to count private recreation spaces: 3 countries counted parcels designated as recreation use, while others counted the number of actual facilities present. Brazil, Czech Republic, and New Zealand counted parcels, which may underestimate the number of recreation facilities present on any parcel. Private recreation density was calculated by dividing the number of parcel/facilities by the entire buffer area.Figure 9 shows that the median private recreation densities ranged from a low of 0.59 places per sq km in Baltimore, USA to a high of 3.64 places per sq km in Aarhus, DNK. A linear-type pattern in median densities was observed across countries. The pooled median private recreation density was 1.16 private recreation places per sq km.

## Discussion

This paper demonstrated the feasibility of creating comparable GIS-derived variables that characterize built environment features relevant to physical activity from urban and suburban regions of 11 diverse countries in the IPEN Adult Study. A key rationale for IPEN was that international studies are required to maximize variability of environments to avoid underestimating associations between built environments and physical activity. Using the common design and measures of IPEN, the detailed GIS templates, and the comparability evaluation, results showed large within-city variability and very large between-city variability in built environments. These results support the feasibility of the IPEN goal of improving on methodological limitations of prior studies.

Early work to create GIS templates revealed the need for clear operational definitions and a hierarchy of possible variables that could be completed across countries. The comparability evaluation uncovered the need to make GIS analyst decisions transparent to arrive at comparable variables. The methodological templates described here were informed by the detailed GIS procedures of Forsyth et al. [53], but the IPEN templates demonstrate methods and a framework now known to be feasible to implement across numerous countries on five continents. Other investigators with interests in international built environment research can use the templates and comparability evaluation to create variables that can provide the basis for further built environment-physical activity comparisons across countries.

Profound differences in physical activity-related built environments were documented across countries. As expected, for most variables the variability in built environment features was greater between countries where cultural norms, urban development approaches, and transit investments vary considerably. There was a 41-fold difference in medians of residential density, a 5-fold difference in medians of intersection density, and an 18-fold difference in median park density across cities and regions. Comparing variation in the walkability index across regions with diverse and countries and cultures has not been possible before. Previous studies intentionally created a standardized score based on each city [10, 54, 55]. In the present study we were able to create a walkability index based on global variation, so for the first time it was possible to compare 15 cities, which revealed a difference of 9 standard deviations in walkability.

These large differences are the result of periods of development, topography, economic conditions, cultural norms, and to a lesser extent leisure and public health planning practices and recent policies [52]. Regions developing before the advent of the train are different than those that developed after, and even more radical shifts occurred after the advent of the car [56]. The IPEN study will help gauge the potential health benefits that could accrue if regions developed more recently around the automobile were transformed in ways that typify more compact, connected and mixed-use regions. The results also demonstrate the degree to which urban environments differ internationally, and these places can now be quantified and ranked for physical activity friendliness at a far more detailed level than previously thought. The very large environmental differences are likely to reveal associations with multiple health, environmental, economic and policy outcomes that studies of single countries with less variability would not be able to detect.

Most countries showed large within-city variations in most built environment variables, which was the goal of the high-low walkability and high-low SES design. Net residential density and intersection density had the lowest within-country variation, but the mixed use variable ranged from 0.0 to 1.0 for the majority of countries. There was substantial variability in the summary walkability measure, with each city covering a different portion of the distribution. Thus, the cities included in the IPEN Adult Study cover a very wide spectrum, and each city seems to make a unique contribution. The high within-city variation enhances the power of single-city and single-country analyses.

### Limitations and strengths

As would be expected with an international study making secondary use of GIS data, there are many limitations in the ways data can be collected and integrated. The study was an effort in making the best use of existing data to measure the built environment in a highly detailed manner that adhered to the principles of an ecological model of physical activity behavior [7]. The current methods relied on land area rather than floor space data to measure mixed use and walkability, which limits the ability to capture the inherently three-dimensional nature of the built environment. Simply put, Hong Kong is a vertical built environment; not a flat one. Transit service was measured using the best available objective data that resulted in a measure of density of transit stops rather than more robust measure of level of service that captures travel time to destinations. Other limitations included consistency by which street connectivity was measured, noting the case of Bogotá, Colombia where local circumstances made it unrealistic to exclude roads that would be considered limited access freeways in North America.

An accomplishment of the present study was development of a set of methods to create a wide range of GIS variables across countries. However, no reported or objective measures, including GIS-derived measures, are perfect. The present study was affected by imprecision that resulted from differences observed across countries in categorization of land uses, availability and resolution of spatial data, and the size of administrative units that determined the spatial basis for sampling within the study design. The IPEN study approach traded some imprecision for external validity. In order to include as many countries as possible in analyses, some variances from the protocol across countries were accepted, and these variances in data and methods certainly introduced error. Variances in methods were unavoidable given the administrative, legal, cultural, and political systems that govern land uses, transportation, and public health in these countries. However, investigators documented these variances in the templates and during the comparability evaluation, making them transparent, and interpretations of results can be adjusted based on known differences in methods.

It was most difficult to create comparable variables for land use mix, because each city and country used a different number of land use categories, and these could not always be reconciled. However, solutions were developed that allowed all countries with GIS data to contribute land use mix variables. Public transportation data also lacked comparability across countries. The transit density in Bogota, COL and Hong Kong, HKG was likely underestimated due to incomplete data and the presence of informal transit networks. Both cities are known for their remarkable public transit systems. Bus stops for Cuernavaca, MEX were imputed when bus lines crossed road intersections, which introduced imprecision. In reality one could signal for a bus to stop anywhere along a route in Cuernavaca. The final public transit variables reported here have known errors, but transit is an important enough policy area that some error was considered acceptable so the relation of public transit access to physical activity could be investigated. Finally, investigators followed common definitions for classifying public parks, but features and amenities within parks could not be assessed.

The production date of the GIS data in each country was not always documented, and it was not possible for the IPEN CC to independently evaluate the accuracy of data. An independent evaluation of accuracy would have required either primary data collection or ground checks for specific variables. We assume there are errors that vary by country. We relied on GIS analysts in each country to conduct analyses, but they were trained in different ways, and their many decisions in creating variables were not directly observed, though they were documented through the template questions.

We were only able to study variables in existing GIS databases, and these variables were collected by multiple governments for various purposes, most of which were not explicitly for public health. Many environmental variables believed to be relevant for physical activity were not available in GIS, such as sidewalk presence and characteristics, intersection characteristics such as pedestrian signals and crosswalk striping, and traffic calming interventions. Ideally, geospatial variables of interest to public health will become routinely collected and available in accessible GIS databases.

## Conclusion

The rapid population growth in cities will be one of the most important global health issues of the 21st century, because there is growing evidence that design of cities affects multiple health outcomes [57]. The design of sustainable, healthy cities means in part that professionals in public health, urban and transportation planning, along with policy-makers need to work together to create health promoting urban environments. A critical component of this collaboration will be to create the relevant knowledge bases to achieve and sustain healthy behaviors such as physical activity. Walkability is a relatively consistent correlate of transportation and overall physical activity, but effect sizes with physical activity have been smaller than expected, in part due to the predominance of single-region or -country studies with limited variation in urban form [27, 28]. The present results indicate the IPEN Adult Study design was successful in documenting large within-country and between-country variation in virtually all built environment variables examined. As expected, between-country variation was particularly striking for residential density, intersection density, and the overall walkability index, but also for park density.

The GIS templates document in detail the methods used to create comparable measures, and other investigators working within multi-cultural contexts can now use these templates to enhance the comparability of their methods and results. The effort devoted to creating comparable GIS variables will allow planned analyses of objective built environment variables in relation to multiple physical activity and health outcomes that are the primary goals of IPEN Adult. It is hoped these results can be used to inform practices and policies in the city planning, transportation, and parks and recreation sectors. Perhaps these methods could be applied for commercial tools, such as Walk Score [58, 59]. An additional benefit of the present study was building capacity for GIS methods and cross-sector research collaborations across diverse countries.

## Electronic supplementary material

Additional file 1: **“Built Environment and Physical Activity: GIS Templates and Variable Naming Conventions for the IPEN Studies”.** (PDF 12 MB)

Additional file 2: Table S1: “Descriptive statistics for built environment variables (1-km buffers) across 15 cities representing 11 countries”. (XLS 46 KB)

Additional file 3: **“Descriptive statistics for built environment variables (500-m buffers) across 15 cities representing 11 countries”.** (XLS 45 KB)

Additional file 4: **“Net residential density (dwellings per km** ^**2**^ **) for participants’ 500-m network buffers across cities and countries”.** (PDF 61 KB)

Additional file 5: **“Land use mix between residential, retail combined, and civic/institutional for participants’ 500-m network buffers across cities and countries”.** (PDF 53 KB)

Additional file 6: **“Intersection densities for participants’ 500-m network buffers across cities and countries”.** (PDF 61 KB)

Additional file 7: **“Walkability scores across cities and countries within participants’ 500-m network buffer”.** (PDF 60 KB)

Additional file 8: **“Public transportation stop density using participants’ 500-m network buffers across cities and countries”.** (PDF 60 KB)

Additional file 9: **“Density of parks (any size) using participants’ 500-m network buffers across cities and countries”.** (PDF 65 KB)

Additional file 10: **“Density of recreation facilities for participants’ 500-m network buffers across cities and countries”.** (PDF 57 KB)

## Abbreviations

IPEN CC: International physical activity and environment network study coordinating center
GIS: Geographic information systems
SES: Socioeconomic status.
