# Supplementary material for: International variation in neighborhood walkability, transit, and recreation environments using geographic information systems: the IPEN adult study
Source: Int J Health Geogr. 2014 Oct 25;13:43. doi: 10.1186/1476-072X-13-43 (PMC4221715; doi:10.1186/1476-072X-13-43)
Supplement: Supplementary file 1 — Additional file 1: “Built Environment and Physical Activity: GIS Templates and Variable Naming Conventions for the IPEN Studies”. (PDF 12 MB) [file 12942_2014_609_MOESM1_ESM.pdf]

# Built Environment and Physical Activity: GIS Templates and Variable Naming Conventions

For the IPEN Studies*

April 2012

Marc A. Adams, Ph.D.[1]

James Chapman, MSCE[2]

James F. Sallis, Ph.D.[1]

Larry D. Frank, Ph.D.[2]

& International Physical Activity and Environment Network (IPEN) Study Coordinating Center[1]

[1]University of California, San Diego

[2] Urban Design for Health, Inc.

*This comprehensive set of documents was created as part of the International Physical Activity and Environment Network (IPEN) Study. It attempts to provide a common set of built environment definitions and measurement procedures for investigators in the IPEN study. This set of documents represents an evolving product that will be updated as needed for the Adult and Adolescent studies.

# Table of Contents


1. **Template Instructions**
2. **Variable Naming Instructions**
3. **Neighborhood Buffers**
4. **Residential Density and Land Use**
5. **Commercial/Retail Land Use**
6. **Civic and Institutional Land Use**
7. **Entertainment Land Use**
8. **Recreational Land Use**
9. **Food-related and Restaurant Land Use**
10. **Intersection Density**
11. **Public Transportation**
12. **Private Recreation Facilities**
13. **Public Parks**
14. **500 meter Street Network Buffers Variable Names**
15. **1 km Street Network Buffers Variable Names**
16. **500 meter Pedestrian Enhanced Buffers Variable Names**
17. **1 km Pedestrian Enhanced Buffers Variable Names**
18. **Park Distance Variable Names**
19. **Character Key for Variable Names**

# SECTION 1: IPEN GIS TEMPLATES INTRODUCTIONS

These templates provide greater specificity to common concepts, clearer definitions, and guidance on required, desired, and speculative variables and GIS procedures. We believe that following these templates closely will ensure that the IPEN study has a common set of comparable GIS variables, and can document any deviations where necessary. This process is important for maximizing comparability across countries. Please read carefully.

Note the nomenclature used in the GIS templates:

- **Desired variable** means that this variable has been judged to be of greater importance or higher quality. Desired variables should be calculated **in addition** to the required variables.
- **Required variable** means that this variable has been judged to be the lowest common denominator (most likely to be completed) across all countries. **All countries should produce the required variables,** if you have information on that built environment factor.
- **Speculative variable** means that it is unknown whether this variable can be completed by a subset of countries. Speculative variables should be calculated but considered exploratory. However, these variables may be very important for future consideration.

- **Recommended procedures** should be used **instead of** acceptable procedures. Recommended procedures have been judged to be more precise methods of calculating the variables.
- **Acceptable procedures** should be used if recommended procedures cannot be used, or if recommended procedures have been deemed inappropriate for country-specific reasons. Acceptable procedures are less accurate than desired procedures, but acceptable to use.
- **Speculative procedures** are the least accurate procedural option. Should only be used if required or desired procedures cannot be accomplished.

# SECTION 2: NAMING CONVENTION INSTRUCTIONS

We encourage you to start to name your variables by reviewing the visual diagrams for each concept that corresponds to a template. These diagrams are provided in sections 14 to 18. Do not start with the character key. The character key is a reference for all the diagrams, but it does include overlapping and competing concepts when viewed by character order (first, second, etc.). This occurred because we were limited to 8 characters total, and needed to consider the complex conceptual order that can occur for each visual diagram. If you have variables that are not listed in the Naming Convention, please send a list and we will provide unique variable names for your study.

# SECTION 3: NEIGHBORHOOD BUFFERS

| | |
|---|---|
| **Aim:** | Develop a standardized spatial definition of 'neighborhood' to be used in creating land use and other variables, on which participants will be compared across countries. |
| **Task:** | To create street-network buffers (aggregation polygons) around participants' residences of 500 and 1000 meters (required).<br><br>To create pedestrian-enhanced street-network buffers (aggregation polygons) around participants' residences of 500 and 1000 meters (desired). |
| **Datasets:** | Road network |
| **Definition:** | Street network buffers determine a walkable accessible area around participant residences. "Walkable" means the road network used for buffer creation includes only those roads on which pedestrians are allowed. Roads where pedestrians are prohibited (e.g. limited-access freeways, toll roads) are removed from the network for buffer creation.<br><br>Pedestrian-enhanced street-network buffers include the walkable road network and the addition of any non-motorized paths accessible to pedestrians. |
| **Sidenotes:** | Ensure that trimming limited access roads does not accidentally exclude roads where walking or cycling may occur commonly.<br><br>In the US, ArcGIS software was used for GIS analyses. We recommend creating detailed (not generalized) service area buffers using Network Analyst. The US removed limited access roads before buffer creation, but did not set the trim function in Network Analyst (see Appendix A). |

| **Details:** **Please respond to the questions below:** | Excluded/ NO | Included/ YES |
|---|---|---|
| 1. Did you geocode participants to their street address? If no, please tell us how you geocoded participants residences (e.g. geocoded using the center of participant's block or cross streets). | | |
| 2. Were you able to create 500 meter street-network buffers (required)? | | |
| 3. Were you able to create 1000 meter street-network buffers | | |

| | | |
|---|---|---|
| (required)? | | |
| 4. Were you able to create 500 meter pedestrian-enhanced street-network buffers (desired)? | | |
| 5. Were you able to create 1000 meter pedestrian-enhanced street-network buffers (desired)? | | |
| 6. If you answered "NO" to questions 1-4, what type of buffer did you create? Please describe below: | | |
| 7. Did you create detailed or generalized buffers (an option on some software programs)? | | |
| 8. Was the road network trimmed in any way? | | |
| 8.1. Were limited access freeways (highway interchanges, highway on/off ramps, toll roads) included or excluded from the road network prior to buffer creation? | | |
| 8.2. Were nationally or regionally important highways without limited access included or excluded? Many busy roads can still be used by pedestrians. | | |
| 9. Was bi-directional travel on one-way streets restricted when buffers were created? Our preference is to allow for bi-directional travel because pedestrians can use roads this way **(required)**. | | |
| 10. Was the road network (or buffers) modified or trimmed in another way? If so, please describe below. | | |
| 11. Please tell us which GIS software was used to compute street network buffers and all other variables (e.g. ESRI's ArcGIS)? | | |
| 12. Please tell us the version of this software below: | | |

# Appendix A.

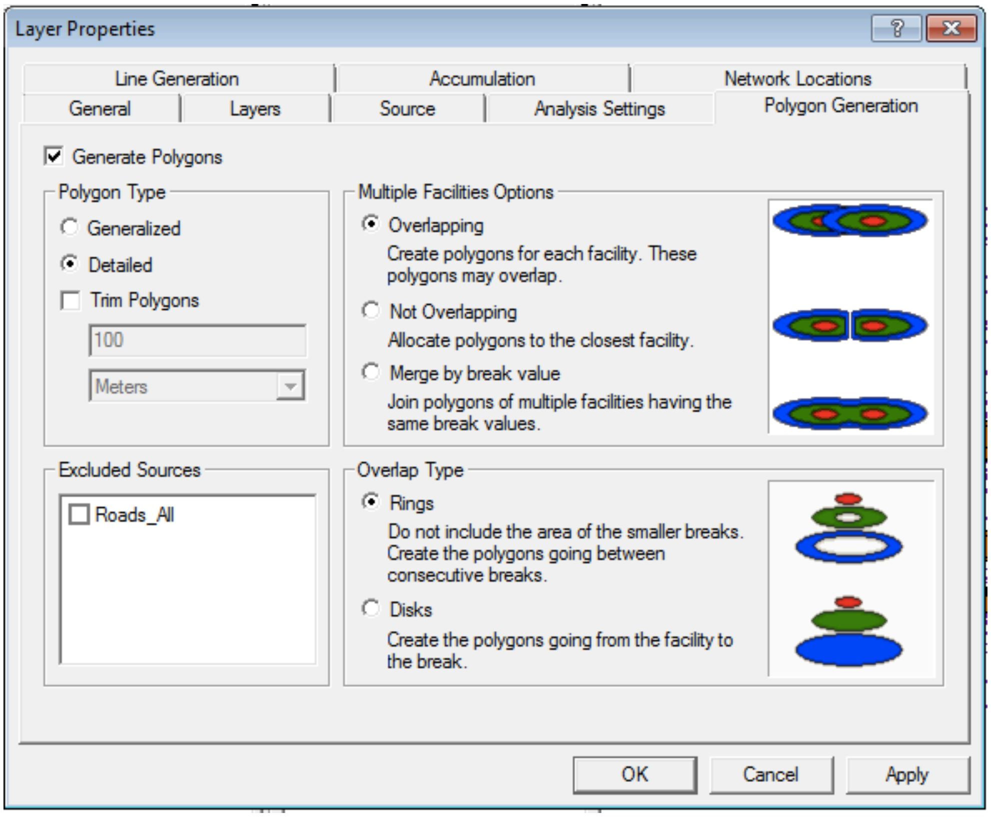

Layer Properties
Line Generation
Accumulation
Network Locations
General
Layers
Source
Analysis Settings
Polygon Generation
Generate Polygons
Polygon Type
Generalized
Detailed
Trim Polygons
100
Meters
Multiple Facilities Options
Overlapping
Create polygons for each facility. These polygons may overlap.
Not Overlapping
Allocate polygons to the closest facility.
Merge by break value
Join polygons of multiple facilities having the same break values.
Excluded Sources
Roads_All
Overlap Type
Rings
Do not include the area of the smaller breaks. Create the polygons going between consecutive breaks.
Disks
Create the polygons going from the facility to the break.
OK
Cancel
Apply

## SECTION 4: RESIDENTIAL DENSITY AND LAND USE

<table>
<tr><td>Aim:</td><td>To develop a standardized definition of <u>residential land use</u> <u>and residential density</u> to be compared across participants across countries. The ‘residential land use area’ sums will be used to calculate the land use mix variable. Net and/or gross density of residential housing units within participants' buffers is part of the walkability index variable.</td></tr>
<tr><td>Tasks:</td><td>• Identify parcels designated as residential.<br>• Calculate the number of single family and the number of multi-family residential parcels within participants’ 500 and 1000 meter buffers (required).<br>• Calculate the sum of single family and the sum of multi-family<br>o land area (required) and/or<br>o building floor area (desired) and/or<br>o building footprint area (speculative)<br>within participants’ 500 and 1000 meter buffers.<br>• Calculate the gross residential density (required) and/or net residential density (desired) within participants’ 500 and 1000 meter buffers.</td></tr>
<tr><td>Datasets:</td><td>Buffers; parcel land use data including land and/or building floor area; spatially referenced housing unit data</td></tr>
<tr><td>Definition:</td><td>Residential density is a ratio of residential housing units (preferred) or population (alternative) (numerator) to the land area devoted to residential use in the road network buffer (denominator). When only residential land area is used in the denominator (the desired way) then net-residential density is calculated. When the total land area of a buffer (all uses) is used then gross-residential density is calculated (required).<br><br>For the denominator, include housing where people live permanently, year-round (mostly), in non-moveable (at least not easily moved housing/dwelling units (e.g. recreational mobile homes are easily moved). Include single and multiple family housing units. Include mobile home parks and school-related housing (e.g. dormitories/apartments). Do NOT include temporary housing (e.g. hotels, motels, hostels) or institutionalized restricted living (e.g. prisons, hospitals, nursing homes).<br><br>Total ‘residential land use area’ is defined as the sum of the single family and multi-family land area (acreage) of all parcels with a residential land use within a participant’s buffer (required). The</td></tr>
</table>

| | |
|---|---|
| | single family sums and the multi-family sums should be provided separately, as well as combined.<br><br>Total 'residential building floor area' is defined as the sum of the single family and multi-family building floor area (from all floors) of all residential buildings within a participant's buffer (**desired**). The single family sums and the multi-family sums should be provided separately, as well as combined.<br><br>Total 'residential building footprint area' is defined as the sum of the single family and multi-family building footprint area of all residential buildings within a participant's buffer (**speculative**). The single family sums and the multi-family sums should be provided separately, as well as combined. |
| **Side notes:** | The goal is for each country to use the best available source of information. Use the best available data and methods, which most accurately report the number of housing units on each parcel.<br><br>In the U.S. housing unit totals from Census blocks were used as control totals. These counts were apportioned to specific residential parcels, within the blocks, based on the type of residential use on each parcel. This was done because of the lack of complete and reliable parcel-level housing unit counts in the provided parcel data.<br><br>The following criteria were used in the U.S. to apportion the census control totals to each residential parcel and to distinguish between Multi-Family and Single Family Housing Units.<br>*(Note: The following process requires that each parcel is already identified with some differentiation of the type of residential present on the parcel.)*<br>1. In the US the residential parcels within each block were determined.<br>2. Any available attribute information in the parcel data was used as part of the apportionment method. A housing unit count was assigned to each parcel based on the residential land use type provided in the parcel data. This assigned count was then used to apportion the census control total housing unit count. Steps 3 and 4 below describe the process to assign counts based on the parcel land use classification, and |

| | |
|---|---|
| | guidance is given on what types of uses are single family and which are multi-family. See the text in parentheses for guidance on the housing unit count.<br>3. For Single Family (SF):<br>    a. Single housing unit under one roof (count equals one).<br>        i. Exceptions--include as single family the following:<br>            1. "Semi-attached" (count equals number of semi-attached units).<br>            2. Duplex (two) homes connected by a wall, under same roof (count equals number of duplex structures times two).<br>            3. House with accessory apartment (count equals number of houses with accessory apartments times two)<br>4. For Multi-Family (MF):<br>    a. Three or more housing units under one roof (count equals the number of separate units under one roof)<br>        i. Exception: a "mobile home park" is considered multi-family even though the mobile homes are not connected to one another under one roof. They are considered multi-family due the levels of densities achieved by these small homes on typically small lots (count equals number of separate mobile homes).<br>        ii. Duplexes (two attached units) are not considered multi-family due to the structure and size typically being similar to two single family homes that are attached.<br>5. Once all residential parcels have been assigned a housing unit count (based on the parcel land use categories) the total SF and MF counts are summed for the residential parcels in each block, and then are used to assign the census based control totals.<br>    a. For example, a census block with 100 housing units (according to the census) contains 30 residential parcels. 20 of these parcels are single family and 10 are multi-family.<br>        i. Each single family parcel is assigned one census unit each (leaving 80 to distribute across the 10 multi-family parcels).<br>        ii. If nothing else is known about these multi-family |

| | | | |
|---|---|---|---|
| | parcels (e.g. more disaggregate groupings (such as triplexes, 4-10 units, 25 plus units) or building floor area) then each multi-family parcel is assigned 8 units. If information is present to make a more refined, differentiated assignment of the 80 units to each parcel then that should be used.<br>6. Once each residential parcel is assigned a census-based housing unit count the parcels' census unit SF and MF counts can be individually summed to each participant buffers.<br><br>Parcels should be used instead of land cover data in the denominator, if available. See Appendix A. | | |
| **Details:** | **Please respond to the questions below:** | Excluded / NO | Included / YES |
| 1. Did you use the residential land use definitions provided above? | | | |
| a. Did your residential land use definition differ from the one provided above in any way? Please describe below: | | | |
| 2. Did you use parcels or land cover to calculate land use (See appendix A for an example graphic.)? If neither, please describe below the areal unit that has land use category attributes in your country. | | | |
| 3. Did you sum the number of residential parcels within participants' buffers (**required**)? | | | |
| a. Did you sum the single family amount separate from the multi-family? | | | |
| 4. Did you sum residential land area (**required**)? | | | |
| a. Did you sum the single family amount separate from the multi-family? | | | |
| 5. Did you sum residential building floor area (**desired**)? | | | |
| a. Did you sum the single family amount separate from the multi-family? | | | |
| *6.* Did you sum residential footprint area (**speculative**)? | | | |
| a. Did you sum the single family amount separate from the | | | |

| | | |
|---|---|---|
| multi-family? | | |
| 7. Did you calculate net residential density (**desired**)? | | |
| a. Did you use 'residential land area' in the denominator (**desired**)? If not, please explain your calculation below: | | |
| b. Did you calculate single family net residential density separate from multi-family net residential density? | | |
| 8. Did you calculate gross residential density (**required**)? | | |
| a. Did you calculate single family gross residential density separate from multi-family gross residential density? | | |
| *9. For the numerator, there are different types of counts. Please indicate below if you used housing units, people, or both.* | | |
| a. For the numerator, did you use a count of housing units/dwellings? (**recommended**) What is the source of this data? | | |
| b. For the numerator, did you use a count of people? (**acceptable**) What is the source of this data? | | |
| c. How was the number of housing units or people in each participant's buffer determined? | | |
| d. If you used a count of people, what is an average number of people per household? | | |
| 10. For the denominator, did you use the residential land use definitions provided above? | | |
| *e.* Did you include temporary or institutional housing? | | |
| *f.* Did you include other housing types? | | |
| *g.* Did your land use definition differ from the one provided above in any other way? Please describe below: | | |
| *11.* Did you use "land use area" to calculate the denominator for residential land use? | | |
| *12. For the denominator, there are different methods of handling residential land use polygons that intersect and partially overlap participants' buffers. Please indicate which one of these procedures you used:* | | |
| a. An <u>entire residential parcel</u> was included in the sum of 'residential land area' if a residential parcel's <u>centroid</u> was | | |

| | | |
|---|---|---|
| contained within the buffer polygon (**acceptable**). | | |
| b. An entire residential parcel was included in the sum of 'residential land area' if any portion of a residential-parcel polygon intersected the buffer polygon (**recommended**). | | |
| c. Only a partial area of each residential parcel intersecting the buffer polygon was assigned to the buffer polygon. That is, land area based apportionment was used to assign the proportion of the residential parcel area to the aggregation polygon (**speculative**). | | |
| d. We used another method of handling the partial overlap of residential land use on buffer polygons (**speculative**). Please describe below: | | |
| *13. For the denominator, there are different methods for handling vertically mixed buildings (e.g. 1st floor retail/ 2nd floor office/ 3ed floor residential).* | | |
| a. Does your parcel or land use dataset include an indicator (code) of vertically mixed buildings? | | |
| b. For vertically mixed buildings, does your calculation of residential land area equal the total land area associated with the building? (**recommended**) | | |
| c. For vertically mixed buildings, does your residential land area equal some proportion of the total land area associated with the building? (**acceptable**) | | |
| d. For vertically mixed buildings, did you use another approach? If so, please describe below. | | |
| *14. For the denominator, there are different methods for handling the apportionment of total parcel land area between multiple, single use building (e.g. apartment building next to office building) on the same parcel.* | | |
| a. Did you proportionally divide total parcel land area between the different buildings based on the area of their footprint (ground floor area)? | | |
| b. Did you equally divide total parcel land area between the | | |

| | | |
|---|---|---|
| different buildings? | | |
| c. Did you use another approach? If so, please describe below. | | |

**Appendix A.**

Figure 1: Land Cover Data (left) vs. Parcel Data (right). Source: Urban Design 4 Health, Inc.

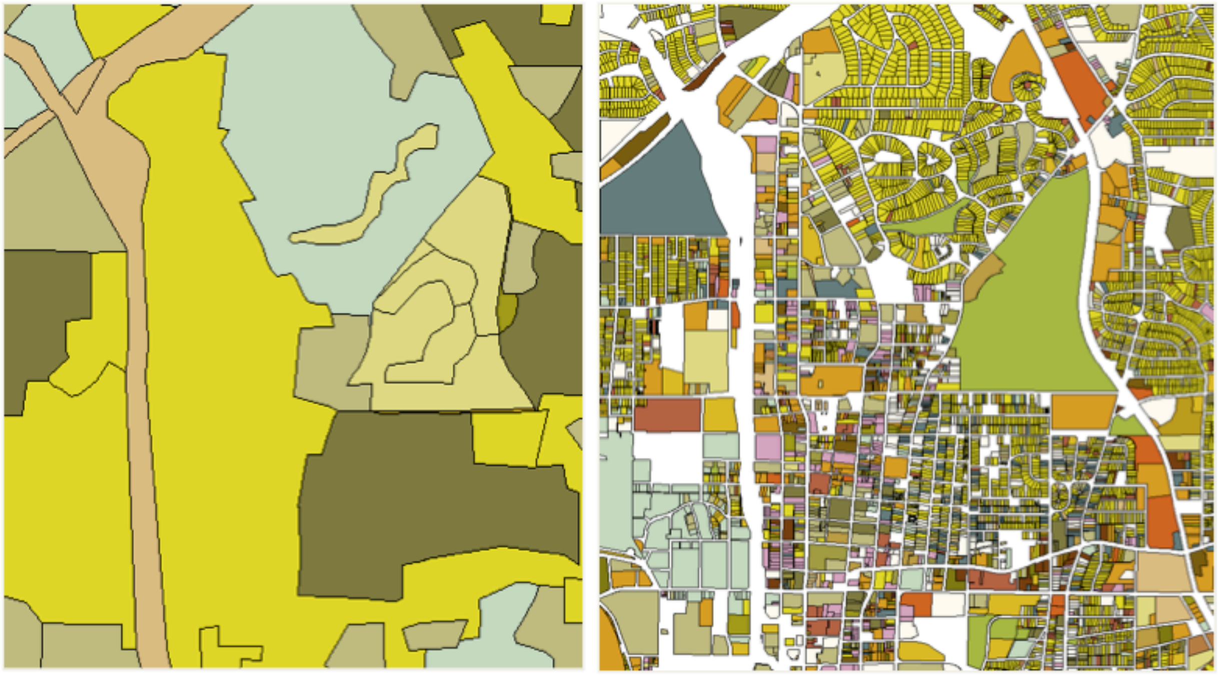

# SECTION 5: COMMERCIAL/RETAIL LAND USE

| | |
|---|---|
| **Aim:** | To develop a standardized definition of **retail land use** that can be compared across participants across countries. Many investigators will not have retail floor space. Identifying retail parcels is necessary to count the number of retail locations, retail land area or retail building floor area (if available). Numbers of retail uses and land area in retail use also creates predictive land use measures. Those that have retail floor area should also calculate a retail floor area measure and a floor space based land use mix measure both of which are part of the walkability index variable. |
| **Tasks:** | Identify parcels designated as retail.<br>Calculate the number of retail parcels within participants' 500 and 1000 meter buffers (**required**).<br>Calculate the sum of the land area for the retail parcels (**required**) and/or building floor areas (**desired**) and/or building footprint areas (**speculative**) for buildings on retail parcels for within participants' 500 and 1000 meter buffers. |
| **Datasets:** | Land use parcel data with parcel and building floor area attributes; participant buffers |
| **Definition:** | A parcel has a retail land use if participants can shop for certain types of goods and services. Retail land use can include independent retail stores, clustered shops, strip malls, specialty markets, bazaars, and shopping malls (neighborhood, community, or regional). **Please see Appendix A for a list of included and excluded retail land uses.** Some examples of types of retail land use include department stores, banking, gas stations with associated retail store, and clothing shops/boutiques. Excluded are automobile-dependent "region-serving" or "big box" (e.g. Costco, Wal-Mart) stores, and/or uses of 300,000 square feet (27,871 square meters) or larger. Entertainment (e.g. movie and performance theaters), food-related, restaurants, recreation facilities (e.g. health clubs, bowling), educational, civic/institutional, and office land uses are excluded from retail, as they will be classified into their own land use area. Retail land use excludes parcels which contain only automobile parking lots.<br><br>Total 'retail land area' is defined as the sum of the land area (acreage) of all parcels with a retail land use within a participant's buffer (**required**).<br><br>Total 'retail building floor area' is defined as the sum of the building floor area (all floors) of all retail buildings within a participant's buffer (**desired**) (note that some countries may not have building floor area information).<br><br>Total 'retail building footprint area' is defined as the sum of the building |

| | | | |
|---|---|---|---|
| | footprint areas of all retail buildings within a participant's buffer (**speculative**) (note that some countries may not have building footprint area information). | | |
| **Sidenotes:** | All food-related and restaurant land uses (if available) are excluded from retail land use. Food-related and restaurant land uses should be separated into its own land use area variables (similar to entertainment, recreation, civic variables).<br><br>Parcels should be used instead of land cover data, if available.<br>See Appendix B. | | |
| **Details:** | **Please respond to the questions below:** | Excluded/ NO | Included/ YES |
| | | | |
| 15. Did you use the retail land use definitions provided above? | | | |
| a. Did you exclude automobile-dependent "region-serving" or "big box" (e.g. Costco, Wal-Mart) stores, and/or uses of 300,000 square feet or larger in your calculation of retail land use (**required**)? | | | |
| b. Did you exclude parcels classified as parking (**required**)? | | | |
| c. Did your land use definition differ from the one provided above in any other way? Please describe below: | | | |
| 16. Did you use parcels or land cover to calculate land use (See appendix B)? If neither, please describe below the areal unit that has land use category attributes in your country. | | | |
| *17.* Did you sum the number of retail parcels (**required**)? | | | |
| 18. Did you sum retail land area (**required**)? | | | |
| *19.* Did you sum building floor area (**desired**)? | | | |
| 20. Did you sum building footprint areas (**speculative**)? | | | |
| 21. *There are different methods of handling multiple retail polygons that intersect and partially overlap participants' buffers. Please indicate which one of these procedures best describes the method you used:* | | | |
| a. An entire retail parcel was included in the sum of retail land area if the retail parcel's centroid was contained within the buffer polygon (**acceptable**). | | | |

| | | |
|---|---|---|
| b. An entire retail parcel was included in the sum of retail land area if any portion of a retail-parcel polygon intersected the buffer polygon (**recommended**). | | |
| c. Only a partial area of each retail parcel intersecting the buffer polygon was assigned to the buffer polygon. Land area based apportionment was used to assign a proportion of the retail floor area to the aggregation polygon. | | |
| d. We used another method of handling the partial overlap of land use on aggregation polygons. Please describe below: | | |
| 22. *There are different methods for handling vertically mixed buildings (e.g. 1st floor retail/ 2nd floor office/ 3rd floor residential).* | | |
| a. Does your parcel or land use dataset include an indicator (code) for vertically mixed buildings? | | |
| b. For vertically mixed buildings, does your calculation of retail land area equal the total land area associated with the building? (**recommended**) | | |
| c. For vertically mixed buildings, does your retail land area equal a proportion of the total land area associated with the building? (**acceptable**) If so how was the retail proportion determined? | | |
| d. For vertically mixed buildings, did you use another approach? If so, please describe below. | | |
| 23. *There are different methods for handling multiple, single use buildings (e.g. retail store next to office building) on the same parcel.* | | |
| a. How did you determine what proportion of the parcel land area to assign to the retail building(s)? Please describe below: | | |

| | | |
|---|---|---|
| | | |
| b. Did you use another approach? If so, please describe below. | | |

# Appendix A: NQLS Land Use Categories

## Included in retail land use:
## Retail
Shopping Ctr(Nghbrhood)
Shopping Ctr(Community)
Shopping Ctr(Regional)
Shopping Ctr(Maj Retail)
Shopping Ctr(Specialty)
Retail(Line/Strip)
Retail Store
Auto Service Station w/Convenience Store
Auto Service Station w/High Volume Gas Sales
Bank branch
Bank building
Barber Shop or Hair Salon
Store convenience market
Store department
Store discount
Store laundromat
Store liquor
Store lumber yard
Store retail

## Excluded from retail land use:
**Entertainment**
Movie theater
Art gallery/museum/soc srvc
Historic prop(rec/entertain)
Theater, live stage (379)
Theater, cinema (380)

**Restaurants**
Sit-down
Fast food

**Recreational**
Bowling alley (306)

City club (310)
Clubhouse (311)
Country club (314)
Skating rink (405)
Tennis club, indoor (416)
Handball-racquetball club (417)
Health club (418)
Fitness center (483)
Natatorium (485)
Field houses (486)
Arcade (573)

**Civic**
Church with Sunday school (308)
Church (309)
Fire station (staffed) (322)
Government building (327)
Library, public (337)
Fire station (volunteer) (427)
Convention center (482)
Jail - police station (489)
Government community service building (491)

**Office**
Office building
Office park
Condominium(office)
Historic prop(office)

**Educational**
School(public)
School(private)

# Appendix B.

**Figure 1: Land Cover Data (left) vs. Parcel Data (right). Source: Urban Design 4 Health, Inc.**

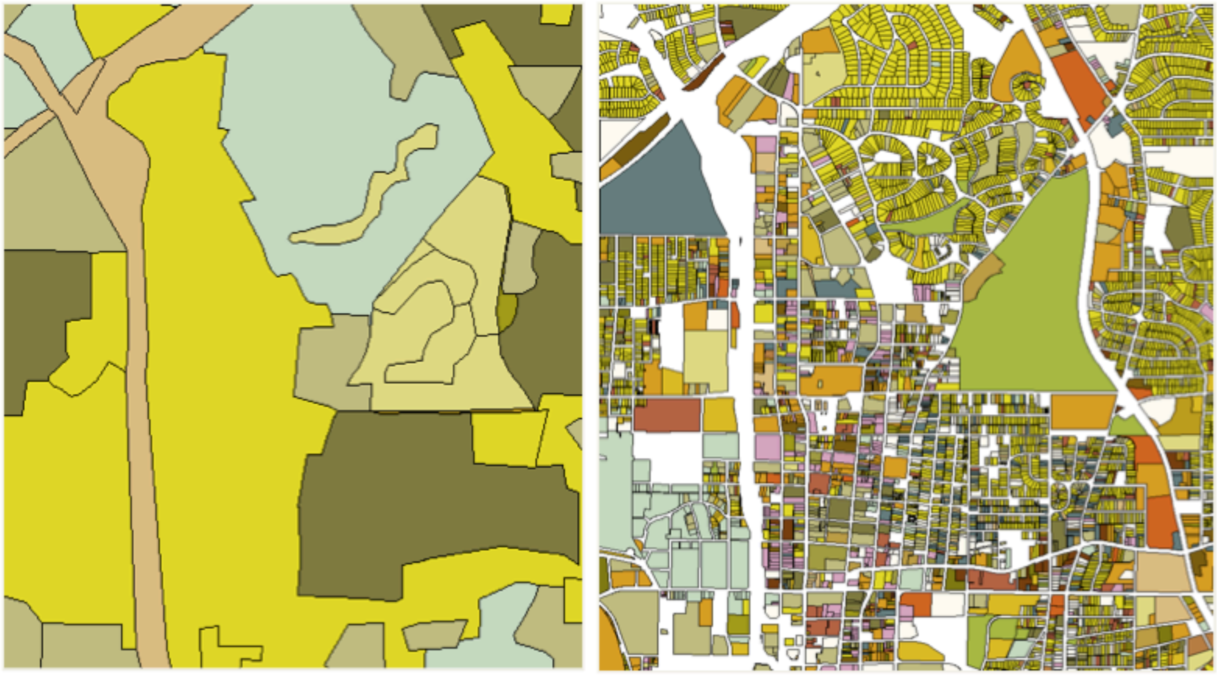

# SECTION 6: CIVIC AND INSTITUTIONAL LAND USE

| | |
|---|---|
| **Aim:** | To develop a standardized definition of **<u>civic and institutional land use</u>** to be compared across participants across countries. The civic/institutional land use area and building floor area (if available) sums will be used to calculate the land use mix variable, which is part of the walkability index variable. |
| **Tasks:** | • Identify parcels designated as civic and institutional.<br>• Count the number of parcels designated as civic and institutional within participants' 500 and 1000 meter buffers (**required**).<br>• Calculate the sum of the land area (**required**) and/or building floor area (**desired**) and/or building footprint area (**speculative**) of civic and institutional land use parcels within participants' 500 and 1000 meter buffers. |
| **Datasets:** | Land use parcel data with land and building floor area attributes; participant buffers |
| **Definition:** | A parcel has a civic and/or institutional land use if the setting is used for educational, religious, health, historical, governmental, correctional, police or military facilities. Civic and institutional land uses may include public facilities such as libraries and some types of private facilities (for non-profit or for profit) such as private colleges. Examples of public civic and institutional facilities include schools, colleges, fire stations, police stations, military bases, government post offices, public works, and other governmental facilities. Private facilities must be related to one of the settings noted above (e.g. private school, private-for-profit hospital). More examples of the types of locations to be designated as civic/institutional are provided in **Appendix A. Please see Appendix A for a list of included and excluded land uses.** Retail, entertainment, food, recreation and office land uses are excluded from civic/institutional land uses, as they will be classified into their own land use type. Civic/institutional land use area can be calculated from land use area or building floor area.<br><br>Total 'civic/institutional land area' is defined as the sum of the land area (acreage) of all parcels with a civic/institutional land use within a participant's buffer<br><br>Total 'civic/institutional building floor area' is defined as the sum of the building floor area (from all floors) of all civic/institutional buildings within a participant's buffer.<br><br>Total 'civic/institutional building footprint area' is defined as the sum of the building footprint area of all civic/institutional buildings within a |

| | | | |
|---|---|---|---|
| | participant's buffer (**speculative**). | | |
| **Sidenotes:** | Parcels should be used instead of land cover data, if available. See Appendix B. | | |
| **Details:** | **Please respond to the questions below:** | Excluded/ NO | Included/ YES |
| | | | |
| 24. Did you use the civic/institutional definitions provided above? | | | |
| a. Did your civic/institutional land use definition differ from the one provided above in any way? Please describe below: | | | |
| 25. Did you use parcels or land cover to calculate land use (See appendix B)? If neither, please describe below the areal unit that has land use category attributes in your country. | | | |
| 26. Did you count the number of civic/institutional parcels within participants' buffers (**required**)? | | | |
| *27.* Did you sum civic/institutional land area (**required**)? | | | |
| *28.* Did you sum civic/institutional building floor area (**desired**)? | | | |
| *29.* Did you sum civic/institutional building footprint area (**speculative**)? | | | |
| 30. *There are different methods of handling multiple civic/institutional polygons that intersect and partially overlap participants' buffers. Please indicate which one of these procedures best describes the method you used:* | | | |
| a. An entire civic/institutional parcel was included in the sum of 'civic/institutional land area' if a civic/institutional parcel's centroid was contained within the buffer polygon (**acceptable**). | | | |
| b. An entire civic/institutional parcel was included in the sum of 'civic/institutional land area' if any portion of an civic/institutional-parcel polygon intersected the buffer polygon (**recommended**). | | | |
| c. Only a partial area of each civic/institutional parcel intersecting the buffer polygon was assigned to the buffer | | | |

| | | |
|---|---|---|
| polygon. That is, land area based apportionment was used to assign the proportion of the civic/institutional parcel area to the aggregation polygon (**speculative**). | | |
| d. We used another method of handling the partial overlap of civic/institutional land use on buffer polygons. Please describe below: | | |
| 31. *There are different methods for handling vertically mixed buildings (e.g. 1st floor* civic/institutional*/ 2nd floor office/ 3rd floor residential).* | | |
| a. Does your parcel or land use dataset include an indicator (code) for vertically mixed buildings? | | |
| b. For vertically mixed buildings, does your calculation of civic/institutional land area equal the total land area associated with the building? (recommended) | | |
| c. For vertically mixed buildings, does your civic/institutional land area equal a proportion of the total land area associated with the building? (acceptable) If so how was the civic/institutional proportion determined? | | |
| d. For vertically mixed buildings, did you use another approach? If so, please describe below. | | |
| 32. *There are different methods for handling the apportionment of total parcel land area between multiple, single use building (e.g.* | | |

| | | |
|---|---|---|
| *civic/institutional facility next to office building) on the same parcel.* | | |
| a. Did you proportionally divide total parcel land area between the different buildings based on the area of their footprint (ground floor area)? | | |
| b. Did you equally divide total parcel land area between the different buildings? | | |
| c. Did you use another approach? If so, please describe below. | | |

## Appendix A Land Use Categories

### Included in civic/institutional land use:

Educational
School(public)
School(private)

CHURCH WITH SUNDAY SCHOOL (308)
CHURCH (309)
FIRE STATION (STAFFED) (322)
GOVERNMENT BUILDING (327)
LIBRARY, PUBLIC (337)
FIRE STATION (VOLUNTEER) (427)
CONVENTION CENTER (482)
JAIL - POLICE STATION (489)
GOVERNMENT COMMUNITY SERVICE BUILDING (491)
JUR Commission for Historical Preservation
JUR Detention Center
JUR Fire Department
JUR Library
JUR Market and Comfort Statio
JUR Police Station
JUR Public Works Property
PUB Military Installation
STA Armory
STA Department of Public Work
STA Metropolitan Transit Authority
STA Police Station/Barrack
STA State Roads Commission (Mass Transit Administration

## Excluded:

**Entertainment**

Movie Theater
Art Gallery/Museum/Soc Srvc
Historic Prop(Rec/Entertain)
THEATER, LIVE STAGE (379)
THEATER, CINEMA (380)
REC Movie Theater
REC Social Club

**Retail**
Shopping Ctr(Nghbrhood)
Shopping Ctr(Community)
Shopping Ctr(Regional)
Shopping Ctr(Maj Retail)
Shopping Ctr(Specialty)
Retail(Line/Strip)
Retail Store
Auto Service Station w/Convenience Store
Auto Service Station w/High Volume Gas Sales
Bank branch
Bank building
Barber Shop or Hair Salon
Store convenience market
Store department
Store discount
Store laundromat
Store liquor
Store lumber yard
Store retail

**Office**
Office building
Office park
Condominium(office)
Historic prop(office)

**Recreational**
Bowling alley (306)
City club (310)
Clubhouse (311)
Country club (314)
Skating rink (405)
Tennis club, indoor (416)
Handball-racquetball club (417)
Health club (418)
Fitness center (483)
Natatorium (485)

Field houses (486)
Arcade (573)

# Appendix B.

**Figure 1: Land Cover Data (left) vs. Parcel Data (right). Source: Urban Design 4 Health, Inc.**

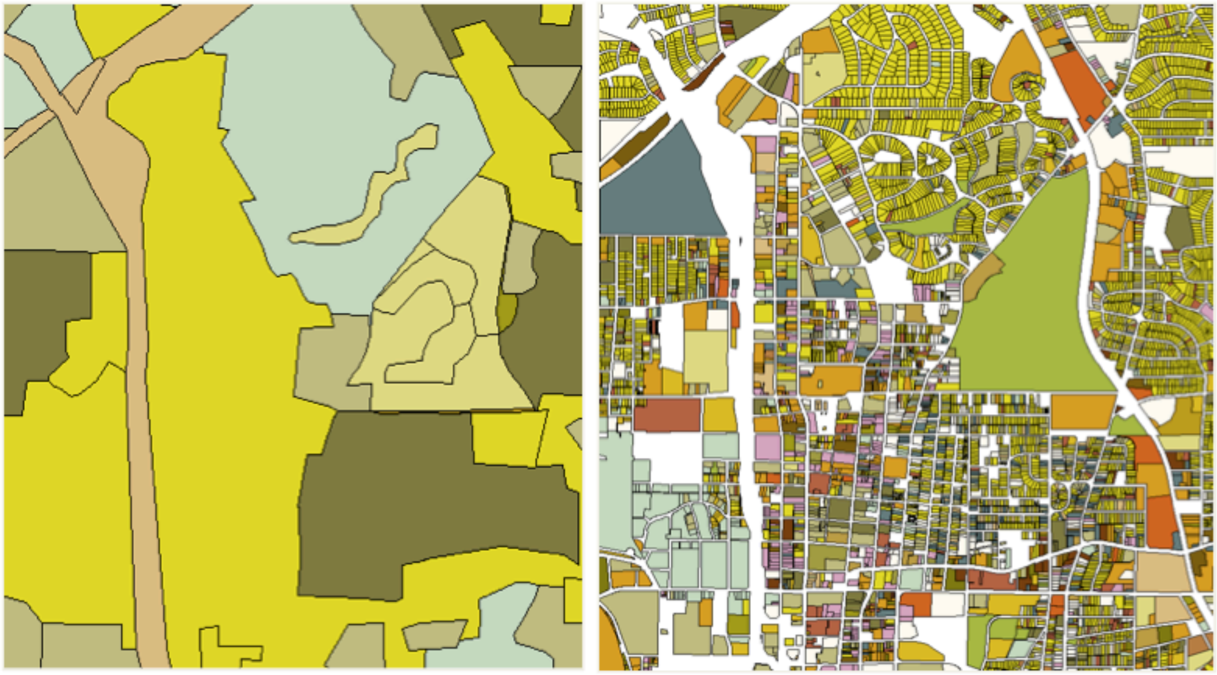

# SECTION 7: ENTERTAINMENT LAND USE

| | |
|---|---|
| **Aim:** | To develop a standardized definition of **entertainment land use** to be compared across participants across countries. The entertainment land use area or building floor space (if available) sums will be used to calculate the land use mix variable, which is part of the walkability index variable. |
| **Tasks:** | • Identify parcels designated as entertainment.<br>• Count the number of parcels designated as entertainment within participants' 500 and 1000 meter buffers (**required**).<br>• Calculate the sum of the land area (**required**) and/or building floor area (**desired**) and/or building footprint area (**speculative**) of entertainment land use parcels within participants' 500 and 1000 meter buffers. |
| **Datasets:** | Land use parcel data with land and/or building floor area attributes; participant buffers |
| **Definition:** | A parcel has an entertainment land use if participants can visit the location for certain types of social activities. Entertainment uses are defined as day or night settings where individuals go to be social with other people or to be entertained. Entertainment uses may include club settings (bars, night clubs), coffee shops, cinemas, theaters, museums, or other social clubs. Excluded from entertainment uses are recreation facilities where people can be social and physically active (e.g. bowling alley, ice skating rinks, country clubs). **Please see Appendix A for a list of included and excluded entertainment land uses.** Retail, recreational, educational, civic/institutional, and office land uses are excluded from entertainment, as they will be classified into their own land use type. Entertainment land use area can be calculated from land use area or building floor area.<br><br>Total 'entertainment land use area' is defined as the sum of the land area (acreage) of all parcels with a entertainment land use within a participant's buffer<br><br>Total 'entertainment building floor area' is defined as the sum of the building floor area (from all floors) of all entertainment buildings within a participant's buffer.<br><br>Total 'entertainment building footprint area' is defined as the sum of the building footprint area of all entertainment buildings within a participant's buffer. |
| **Sidenotes:** | Parcels should be used instead of land cover data, if available. |

| | | | |
|---|---|---|---|
| | See Appendix B. | | |
| **Details:** | **Please respond to the questions below:** | Excluded/ NO | Included/ YES |
| | | | |
| 33. Did you use the entertainment definitions provided above? | | | |
| a. Did your entertainment land use definition differ from the one provided above in any way? Please describe below: | | | |
| 34. Did you use parcels or land cover to calculate land use (See appendix B)? If neither, please describe below the areal unit that has land use category attributes in your country. | | | |
| 35. Did you count the number of entertainment parcels within participants' buffers (**required**)? | | | |
| 36. Did you sum entertainment land area (**required**)? | | | |
| 37. Did you sum entertainment building floor area (**desired**)? | | | |
| 38. Did you sum entertainment building footprint area (**speculative**)? | | | |
| 39. *There are different methods of handling multiple entertainment polygons that intersect and partially overlap participants' buffers. Please indicate which one of these procedures best describes the method you used:* | | | |
| a. An entire entertainment parcel was included in the sum of 'entertainment land area' if an entertainment parcel's centroid was contained within the buffer polygon (**acceptable**). | | | |
| b. An entire entertainment parcel was included in the sum of 'entertainment land area' if any portion of an entertainment-parcel polygon intersected the buffer polygon (**recommended**). | | | |
| c. Only a partial area of each entertainment parcel intersecting the buffer polygon was assigned to the buffer polygon. That is, land area based apportionment was used to assign the proportion of the entertainment parcel area and entertainment related building floor space (if available) to the aggregation polygon (**speculative**). | | | |
| d. We used another method of handling the partial overlap of entertainment land use on buffer polygons. Please describe below: | | | |

| | | |
|---|---|---|
| | | |
| 40. *There are different methods for handling vertically mixed buildings (e.g. 1st floor entertainment/ 2nd floor office/ 3rd floor residential).* | | |
| a. Does your parcel or land use dataset include an indicator (code) for vertically mixed buildings? | | |
| b. For vertically mixed buildings, does your calculation of entertainment land area equal the total land area associated with the building? (**recommended**) | | |
| c. For vertically mixed buildings, does your entertainment land area equal some proportion of the total land area associated with the building? (**acceptable**) If so how was the entertainment proportion determined? | | |
| d. For vertically mixed buildings, did you use another approach? If so, please describe below. | | |
| 41. *There are different methods for handling multiple, single use buildings (e.g. entertainment store next to office building) on the same parcel.* | | |
| a. Did you proportionally divide total parcel land area between the different buildings based on the area of their footprint (ground floor area)? | | |
| b. Did you equally divide total parcel land area between the different buildings? | | |

| | | |
|---|---|---|
| c. Did you use another approach? If so, please describe below. | | |

# Appendix A Land Use Categories

## Included in entertainment land use:

**Entertainment**

Movie Theater
Art Gallery/Museum/Soc Srvc
Historic Prop(Rec/Entertain)
THEATER, LIVE STAGE (379)
THEATER, CINEMA (380)
REC Movie Theater
REC Social Club
Casinos

## Excluded:

**Recreational**

Bowling alley (306)
City club (310)
Country club (314)
Skating rink (405)
Tennis club, indoor (416)
Handball-racquetball club (417)
Health club (418)
Fitness center (483)
Natatorium (485)
Field houses (486)
Arcade (573)

## Retail

Shopping Ctr(Nghbrhood)
Shopping Ctr(Community)
Shopping Ctr(Regional)
Shopping Ctr(Maj Retail)
Shopping Ctr(Specialty)
Retail(Line/Strip)
Retail Store
Auto Service Station w/Convenience Store
Auto Service Station w/High Volume Gas Sales
Bank branch

Bank building
Barber Shop or Hair Salon
Store convenience market
Store department
Store discount
Store laundromat
Store liquor
Store lumber yard
Store retail

**Civic**
Church with Sunday school (308)
Church (309)
Fire station (staffed) (322)
Government building (327)
Library, public (337)
Fire station (volunteer) (427)
Convention center (482)
Jail - police station (489)
Government community service building (491)

**Office**
Office building
Office park
Condominium(office)
Historic prop(office)

**Educational**
School(public)
School(private)

# Appendix B.

Figure 1: Land Cover Data (left) vs. Parcel Data (right). Source: Urban Design 4 Health, Inc.

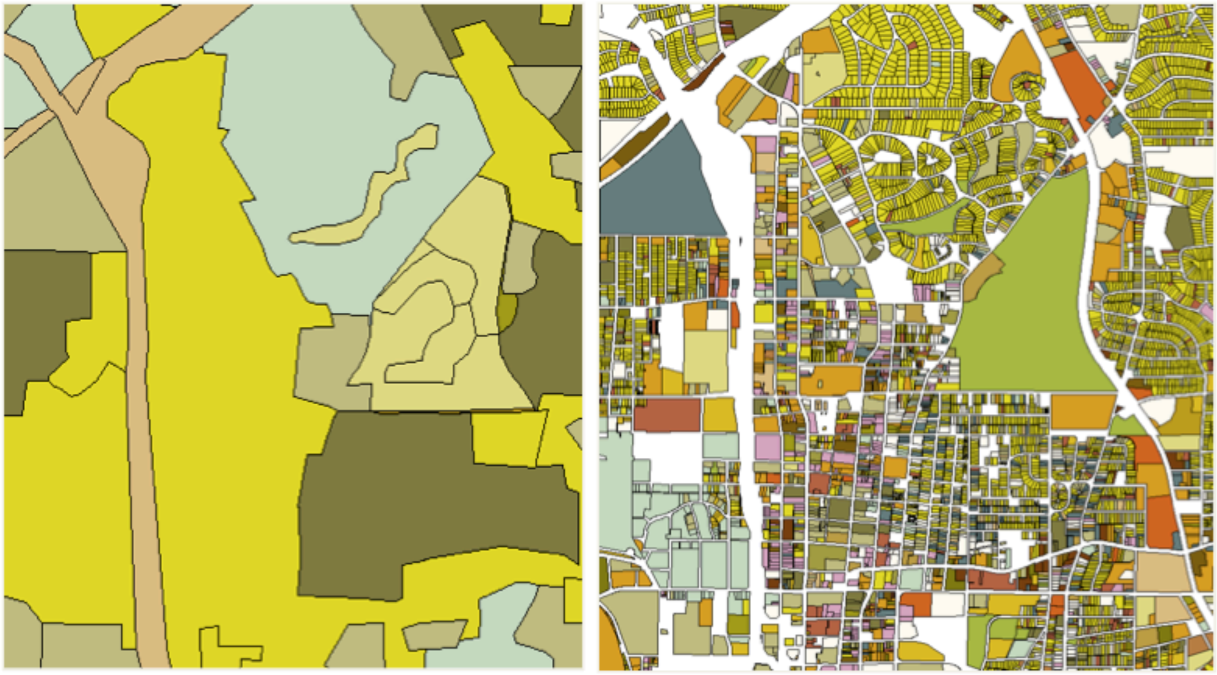

# SECTION 8: RECREATION LAND USE

| | |
|---|---|
| **Aim:** | To develop a standardized definition of public and private **recreation land use** to be compared across participants across countries. The 'recreation land use area' sums will be used to calculate the land use mix variable, which is part of the walkability index variable. **There are separate templates for public park and private recreation facilities for computing count, density, and distance.** |
| **Tasks:** | • Identify parcels designated as public or private recreation.<br>• Count the number of parcels designated as public or private recreation within participants' 500 and 1000 meter buffers (**required**).<br>• Calculate the sum of the land area (**required**) and/or building floor area (**desired**) and/or building footprint area (**speculative**) of recreation land use parcels within participants' 500 and 1000 meter buffers. |
| **Datasets:** | Land use parcel data with building floor area and land area attributes; participant buffers |
| **Definition:** | A parcel has a recreation land use if participants are usually physically active there. Recreation land uses include both public outdoor spaces (e.g. parks, recreational spaces) and private recreation facilities. Examples of private recreation facilities include fitness centers, health clubs, tennis centers, swimming pools, golf courses, outdoor arenas, camp sites, etc. Public outdoor spaces that function as parks are included. Examples of the types of locations to be designated as recreation are provided in **Appendix A.** Excluded from recreation land use are vacant lots (or unusable open space) and outdoor and indoor locations that are not designed for physical activity. **Please see Appendix A for a list of included and excluded recreation land uses.** Retail, entertainment, educational, civic/institutional, and office land uses are excluded from recreation land uses, as they will be classified into their own land use type. Recreation land use area should be calculated from land area and building floor area (if available).<br><br>Total 'recreation land area' is defined as the sum of the land area (acreage) of all parcels with a recreation land use within a participant's buffer (**required**).<br><br>Total 'recreation building floor area' is defined as the sum of the building floor area (from all floors) of all recreation buildings within a participant's buffer (**desired**). Recreation parcels with no building on them (e.g. a public park) will have zero building floor area. |

| | | | |
|---|---|---|---|
| | Total 'recreation building footprint area' is defined as the sum of the building footprint area of all recreation buildings within a participant's buffer (**speculative**). Recreation parcels with no building on them (e.g. a public park) will have zero building footprint area. | | |
| **Sidenotes:** | **NOTE: The tasks described here to identify recreation land use area are different than the tasks described in the template called, "Creation of Private Recreation Count/Density Variables." However, the locations of private recreation locations used for both these tasks are the same. The recreation land use area based variables also includes public recreation locations, unlike the private recreation count/density variables.**<br><br>Parcels should be used instead of land cover data, if available.<br>See Appendix B. | | |
| **Details:** | **Please respond to the questions below:** | Excluded/ NO | Included/ YES |
| | | | |
| 42. Did you use the recreation definitions provided above and in Appendix A? | | | |
| a. Did your recreation land use definition differ from the one provided above in any way? Please describe below: | | | |
| 43. Did you use parcels or land cover to calculate land use (See appendix B)? If neither, please describe below the areal unit that has land use category attributes in your country. | | | |
| 44. Did you count the number of recreational parcels within participants' buffers (**required**)? | | | |
| 45. Did you sum recreation land area (**required**)? | | | |
| 46. Did you sum recreation building floor area (**desired**)? | | | |
| *47.* Did you sum recreation building footprint area (**speculative**)? | | | |
| 48. *There are different methods of handling multiple recreation polygons that intersect and partially overlap participants' buffers. Please indicate which one of these procedures best describes the method you used:* | | | |
| a. An entire recreation parcel was included in the sum of 'recreation land area' if a recreation parcel's centroid was | | | |

| | | |
|---|---|---|
| contained within the buffer polygon (**acceptable**). | | |
| b. An entire recreation parcel was included in the sum of 'recreation land area' if any portion of a recreation-parcel polygon intersected the buffer polygon (**recommended**). | | |
| c. Only a partial area of each recreation parcel intersecting the buffer polygon was assigned to the buffer polygon. That is, land area based apportionment was used to assign the proportion of the recreation parcel area to the aggregation polygon (**speculative**). | | |
| d. We used another method of handling the partial overlap of recreation land use on buffer polygons. Please describe below: | | |
| 49. *There are different methods for handling vertically mixed buildings (e.g. 1st floor recreation/ 2nd floor office/ 3rd floor residential).* | | |
| a. Does your parcel or land use dataset include an indicator (code) for vertically mixed buildings? | | |
| b. For vertically mixed buildings, does your calculation of recreation land area equal the total land area associated with the building (**recommended**)? | | |
| c. For vertically mixed buildings, does your recreation land area equal a proportion of the total land area associated with the building (**acceptable**)? If so how was the recreation proportion determined? | | |
| d. For vertically mixed buildings, did you use another approach? If so, please describe below. | | |

| | | |
|---|---|---|
| | | |
| 50. *There are different methods for handling the apportionment of total parcel land area between multiple, single use building (e.g. recreation facility next to office building) on the same parcel.* | | |
| a. Did you proportionally divide total parcel land area between the different buildings based on the area of their footprint (ground floor area)? | | |
| b. Did you equally divide total parcel land area between the different buildings? | | |
| c. Did you use another approach? If so, please describe below. | | |

## Appendix A Land Use Categories

# Included in recreation land use:

**Recreational**

Bowling alley (306)
City club (310)
Clubhouse (311)
Country club (314)
Skating rink (405)
Tennis club, indoor (416)
Handball-racquetball club (417)
Health club (418)
Fitness center (483)
Natatorium (485)
Field houses (486)
Arcade (573)
Recreation Centers
Senior Centers
Public Parks

## Excluded:

**Entertainment**

Movie Theater
Art Gallery/Museum/Soc Srvc
Historic Prop(Rec/Entertain)

THEATER, LIVE STAGE (379)
THEATER, CINEMA (380)
REC Movie Theater
REC Social Club

**Retail**
Shopping Ctr(Nghbrhood)
Shopping Ctr(Community)
Shopping Ctr(Regional)
Shopping Ctr(Maj Retail)
Shopping Ctr(Specialty)
Retail(Line/Strip)
Retail Store
Auto Service Station w/Convenience Store
Auto Service Station w/High Volume Gas Sales
Bank branch
Bank building
Barber Shop or Hair Salon
Store convenience market
Store department
Store discount
Store laundromat
Store liquor
Store lumber yard
Store retail

**Civic**
Church with Sunday school (308)
Church (309)
Fire station (staffed) (322)
Government building (327)
Library, public (337)
Fire station (volunteer) (427)
Convention center (482)
Jail - police station (489)
Government community service building (491)

**Office**
Office building
Office park
Condominium(office)
Historic prop(office)

**Educational**
School(public)
School(private)

## Appendix B.

Figure 1: Land Cover Data (left) vs. Parcel Data (right). Source: Urban Design 4 Health, Inc.

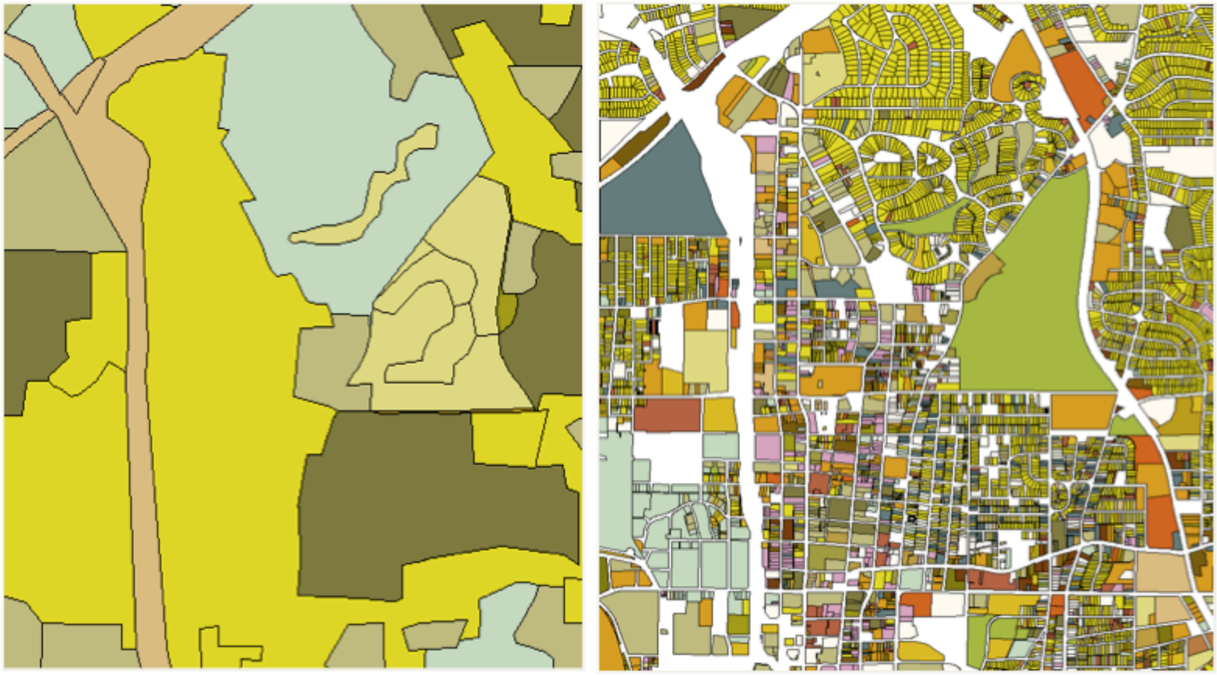

# SECTION 9: FOOD-RELATED AND RESTAURANT LAND USE

| | |
|---|---|
| **Aim:** | To develop a standardized definition of **food-related** and **restaurant land use** (combined and separated, if possible) to be compared across participants across countries. The 'food-related land area' and/or 'food-related building floor space' (if available) sums will be used to calculate the land use mix variable, which is part of the walkability index variable. |
| **Tasks:** | • Identify parcels designated as food-related and restaurant (combined and separately, if possible)<br>• Count the number of parcels designated as food related (**required**) and/or restaurant (**desired**) within participants' 500 and 1000 meter buffers (**required**).<br>• Calculate the sum of the land area (**required**) and/or building floor area (**desired**) and/or building footprint area (**speculative**) of food related and restaurant land use parcels (combined and separately, if possible) within participants' 500 and 1000 meter buffers. |
| **Datasets:** | Land use parcel data with parcel and building floor area attributes; participant buffers |
| **Definition:** | A parcel has a food-related land use if participants primarily visit the location in order to purchase unprepared food, or eat or take away fully prepared food. Food related land uses such as grocery stores, convenience stores, bakeries, restaurants, fast food chains, or any stores mainly selling food should be included (not department stores or shopping malls). Do not include multi-use facilities with restaurants, such as hotels, department stores, shopping malls, and office buildings. **Please see Appendix A for a list of included and excluded restaurant land uses.** Retail, entertainment, recreational, educational, civic/institutional, and office land uses are excluded from food-related land use, as they will be classified into their own land use type.<br><br>A restaurant land use should be calculated in addition to food-related land use if possible. Restaurant land use includes any limited or full service location where you eat or take away fully prepared food. Do not include multi-use facilities described above.<br><br>Total 'food-related land area' is defined as the sum of the land area (acreage) of all parcels with a food-related land use within a participant's buffer (**required**).<br><br>Total 'food-related building floor area' is defined as the sum of the building floor area (from all floors) of all food-related buildings within a participant's buffer (**desired**). |

| | | | |
|---|---|---|---|
| | Total 'food-related building footprint area' is defined as the sum of the building footprint area of all food-related buildings within a participant's buffer (**speculative**).<br><br>Total 'restaurant land area' is defined as the sum of the land area (acreage) of all parcels with a restaurant land use within a participant's buffer (**desired**).<br><br>Total 'restaurant building floor area' is defined as the sum of the building floor area (from all floors) of all restaurant buildings within a participant's buffer (**desired**).<br><br>Total 'restaurant-related building footprint area' is defined as the sum of the building footprint area of all restaurant buildings within a participant's buffer (**speculative**). | | |
| **Sidenotes:** | Parcels should be used instead of land cover data, if available.<br>See Appendix B. | | |
| **Details:** | **Please respond to the questions below:** | Excluded /NO | Included/ YES |
| | | | |
| 51. Did you use the food-related and restaurant definitions provided above? | | | |
| a. Did your food-related land use definition differ from the one provided above in any way? Please describe below: | | | |
| b. Did your restaurant land use definition differ from the one provided above in any way? Please describe below: | | | |
| 52. Did you use parcels or land cover to calculate land use (See appendix B)? If neither, please describe below the areal unit that has land use category attributes in your country. | | | |
| 53. Did you count the number of food-related parcels within participants' buffers (**required**)? | | | |
| 54. Did you sum food-related land area (**required**)? | | | |
| 55. Did you sum food-related building floor area (**desired**)? | | | |

| | | |
|---|---|---|
| 56. Did you sum food-related building footprint area (**speculative**)? | | |
| *57.* Did you count the number of restaurant parcels within participants' buffers (**desired**)? | | |
| *58.* Did you sum restaurant land area (**desired**)? | | |
| *59.* Did you sum restaurant building floor area (**desired**)? | | |
| *60.* Did you sum restaurant building footprint area (**speculative**)? | | |
| 61. *There are different methods of handling multiple food-related and* restaurant *polygons that intersect and partially overlap participants' buffers. Please indicate which one of these procedures best describes the method you used:* | | |
| a. An entire food-related parcel was included in the sum of 'food-related land/building floor area' if a food-related parcel's centroid was contained within the buffer polygon (**acceptable**). | | |
| b. An entire food-related parcel was included in the sum of 'food-related land/building floor area' if any portion of a food-related parcel polygon intersected the buffer polygon (**recommended**). | | |
| c. Only a partial area of each food-related parcel intersecting the buffer polygon was assigned to the buffer polygon. That is, land/building floor area based apportionment was used to assign the proportion of the food-related parcel area to the aggregation polygon (**speculative**). | | |
| d. We used another method of handling the partial overlap of food-related land use on buffer polygons. Please describe below: | | |
| 62. *There are different methods for handling vertically mixed buildings (e.g. 1st floor food-related/ 2nd floor office/ 3rd floor residential).* | | |
| a. Does your parcel or land use dataset include an indicator | | |

| | | |
|---|---|---|
| (code) for vertically mixed buildings? | | |
| b. For vertically mixed buildings, does your calculation of food-related land area equal the total land area associated with the building? (**recommended**) | | |
| c. For vertically mixed buildings, does your food-related land area equal a proportion of the total land area associated with the building? (**acceptable**) If so, how was the food-related proportion determined? | | |
| d. For vertically mixed buildings, did you use another approach? If so, please describe below. | | |
| 63. *There are different methods for handling multiple, single use buildings (e.g.* grocery *store next to office building) on the same parcel.* | | |
| a. Did you proportionally divide total parcel land area between the different buildings based on the area of their footprint (ground floor area)? | | |
| b. Did you equally divide total parcel land area between the different buildings? | | |
| c. Did you use another approach? If so, please describe below. | | |

# Appendix A Land Use Categories

## Included in Restaurant land use:

**Restaurant**

RESTAURANT Banquet/Catering Facility

RESTAURANT Bar/Tavern

RESTAURANT Converted Dwelling

RESTAURANT Family Style

RESTAURANT Fast Food

STORE- Food store

Store convenience

Grocery store

Market

## Excluded:

**Recreational**

Bowling alley (306)
City club (310)
Clubhouse (311)
Country club (314)
Skating rink (405)
Tennis club, indoor (416)
Handball-racquetball club (417)
Health club (418)
Fitness center (483)
Natatorium (485)
Field houses (486)
Arcade (573)

## Entertainment

Movie Theater

Art Gallery/Museum/Soc Srvc

Historic Prop(Rec/Entertain)

THEATER, LIVE STAGE (379)

THEATER, CINEMA (380)

REC Movie Theater

REC Social Club

## Retail

Shopping Ctr(Nghbrhood)
Shopping Ctr(Community)
Shopping Ctr(Regional)
Shopping Ctr(Maj Retail)
Shopping Ctr(Specialty)
Retail(Line/Strip)

Retail Store
Auto Service Station w/Convenience Store
Auto Service Station w/High Volume Gas Sales
Bank branch
Bank building
Barber Shop or Hair Salon
Store convenience market
Store department
Store discount
Store laundromat
Store liquor
Store lumber yard
Store retail

**Civic**
Church with Sunday school (308)
Church (309)
Fire station (staffed) (322)
Government building (327)
Library, public (337)
Fire station (volunteer) (427)
Convention center (482)
Jail - police station (489)
Government community service building (491)

**Office**
Office building
Office park
Condominium(office)
Historic prop(office)

**Educational**
School(public)
School(private)

# Appendix B.

Figure 1: Land Cover Data (left) vs. Parcel Data (right). Source: Urban Design 4 Health, Inc.

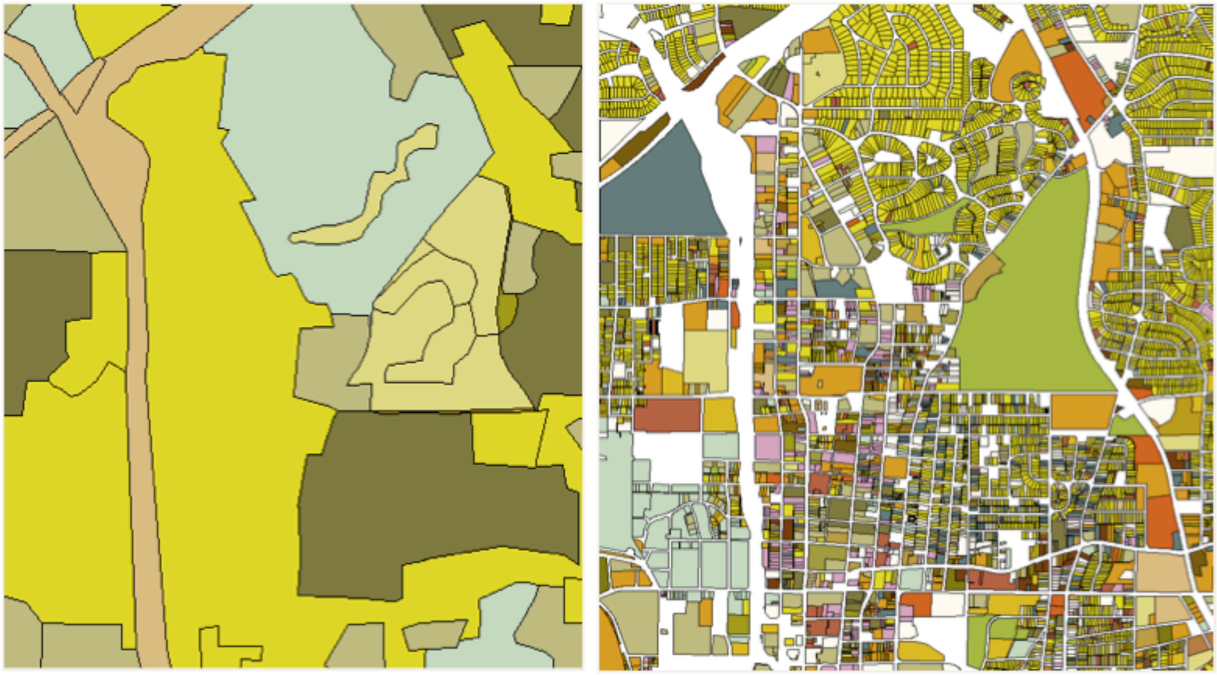

# SECTION 10: INTERSECTION DENSITY

| | | | |
|---|---|---|---|
| **Aim:** | Develop a standardized definition of **intersection density** to be compared across participants across countries. | | |
| **Task:** | To identify and count intersections on a walkable road network that are within participants' 500 and 1000 meter buffers.<br><br>To divide the buffer-level intersection counts by the total buffer land area, thereby creating intersection density (counts per sq km). | | |
| **Datasets:** | Road network and participant buffers | | |
| **Definition:** | The same walkable road network used for buffer creation should be used here.<br><br>Roads where pedestrians are prohibited such as freeways and ramps are removed from the network before intersections are identified and counted.<br>For example, limited-access freeways, toll roads, on and off ramps to them, interchanges between these road types should be removed from the network.<br><br>"Intersection" means a point where 3 or more walkable road segments intersect. | | |
| **Sidenotes:** | Some countries may need to buffer intersection points because some segments in the road network were not properly aligned. Investigators should decide whether spatial misalignment is a problem or not. If it is a problem, then each country should decide the appropriate buffer size after observing their data. | | |
| **Details:** | **Please respond to the questions below:** | Excluded /NO | Included /YES |
| | | | |
| 64. Was the same walkable road network used for buffer creation used for the creation of intersection density? If a different road network was used how was it different? What was the reason for using a different road network? Please describe below: | | | |
| 65. Were pseudo-nodes (i.e. nodes that split road segments at non-intersections) and cul-de-sacs removed from the road network | | | |

| | | |
|---|---|---|
| prior to counting intersections (**required**)? If not, please explain why: | | |
| 13. Were intersections with *3 or more* walkable road segments only counted (**required**)? If not, please describe what types of intersections were counted: | | |
| 14. Was there a need to buffer intersection points because some segments in the road network were not properly aligned?<br><br>14.1. If so, what size buffer did you use to define intersections with slight spatial mismatch (e.g. 15 meters)?<br><br>14.2. If not, do you have a sense whether spatial mismatch was an issue in your road network dataset? Please describe: | | |
| 15. To calculate density, did you divide intersection counts by the total land area for the buffer (**required**)? If not, please describe your calculation: | | |
| 16. Do you have information on whether or not intersections are signalized? | | |
| 17. If so, can you create a variable for density of signalized and unsignalized intersections (**desired**)? | | |

## SECTION 11: PUBLIC TRANSPORTATION

| | | | |
|---|---|---|---|
| **Aim:** | To develop a standardized definition of access to **public transportation** to be compared across participants across countries, and create related public transportation access variables. | | |
| **Tasks:** | 1. Identify locations designated as public rail stations and bus stops.<br><br>2. Count the number of public rail stations and bus stops (combined and separately, if possible) within participants' 500 and 1000 meter buffers (**combined required, separate desired**).<br><br>3. Calculate a public rail station and bus stop density (combined and separately, if possible) for each buffer (count/divided by total land area) (**combined required, separate desired**).<br><br>4. Calculate the walkable-road network based distance from each participant to the nearest public rail station and bus stop (combined and separately, if possible) (**combined required, separate desired**). | | |
| **Datasets:** | Public rail stations and bus stop locations; walkable road network, participant buffers | | |
| **Definition:** | Public transportation includes services that operate on a published schedule, which has fixed routes and locations where people can get on or get off. Public transportation can include all types of vehicles, such as commuter rail, subway, elevated rail, light rail, bus, bus rapid transit, trolley, etc. Typically a larger, regional network of transit can be accessed from any given station/stop by means of transfers to other routes at connecting points. Our definition of public transportation does not include taxi stands, bicycle sharing stations, and private van and shuttle services with no fixed routes and operate on an as needed basis. | | |
| **Sidenotes:** | The same walkable road network used for buffer creation should be used here. "Walkable" road network means roads where pedestrians are prohibited are removed from the network before intersections are identified and counted. | | |
| **Details:** | **Please respond to the questions below:** | Excluded/ NO | Included/ YES |
| | | | |
| 66. Did you use the public transit station/stop definitions provided above? | | | |
| a. If the definition you used differed from the one provided above in any way? Please describe below: | | | |

| | | |
|---|---|---|
| 67. What types of public transportation serve the stations/stops you used? Potential types include: | | |
| a. Commuter rail | | |
| b. Subway | | |
| c. Elevated rail | | |
| d. Light rail | | |
| e. Bus | | |
| f. Bus rapid transit | | |
| g. Trolley | | |
| h. Other? Please describe below | | |
| | | |
| 68. Did you create count variable for all transportation options (**required**)? | | |
| 69. Did you create count variable for specific types of transit (rail versus bus) (**desired**)? | | |
| 70. Did you create a density variable that included all possible transportation options (**required**)? | | |
| 71. Did you create a density variable that included specific types (rail versus bus) separately (**desired**)? | | |
| 72. Did you create distance variable for all transportation options (**required**)? | | |
| 73. Did you create distance variable for specific types of transit (rail versus bus) (**desired**)? | | |
| 74. What unit is the density measurement in (e.g. number/square km)? | | |
| 75. What unit is the distance measurement in (e.g. meters, kilometers)? | | |
| 76. Was the same walkable road network used for buffer creation also used to determine the distance to the nearest transportation location? If a different road network was used how was it different? What was the reason for using a different road network? Please describe below: | | |

| | | |
|---|---|---|
| | | |
| 77. Do you have other types of transit level of service data or can you create other measures from existing variables? These may include travel time based measures, time span between trains or buses (headway), numbers of routes, or others (**speculative**). Please describe below: | | |

## SECTION 12: PRIVATE RECREATION FACILITIES

| | | | |
|---|---|---|---|
| **Aim:** | To develop a standardized definition of **private recreation facilities** to be compared across participants across countries, and create count and road network-based distance variables. | | |
| **Tasks:** | 5. Identify locations designated as private recreation.<br>6. Count the number of private recreation facilities within participants' 500 and 1000 meter buffers.<br>7. Calculate a private recreation facilities density for each buffer (count/divided by total land area).<br>8. Calculate the walkable-road network based distance from each participant to the nearest private recreation facility | | |
| **Datasets:** | Enumerated list of private recreation locations; walkable road network, participant buffers | | |
| **Definition:** | A recreation facility is one where participants can usually be physically active there. Examples of private recreation facilities include fitness centers, health clubs, tennis centers, swimming pools, golf courses, outdoor arenas, camp sites, etc. Public parks are not private recreation locations.<br><br>A location is designated as a private recreation if the facility is not open to the public for free. A private recreation location requires a payment/membership to use the facilities. Examples of such places include fitness centers, health clubs, tennis clubs, etc. The full list of the types of locations to be designated as private recreation is provided in **Appendix A.**<br><br>In the U.S., enumerated lists were based on business listings, phone book listings, marketing firm's address lists, other online internet sources, and parcel data. | | |
| **Sidenotes:** | Check accuracy of parcel data against phone books in a sample. If inaccurate, then supplement parcel data with phone book and other data. Best to use multiple sources of recreation facility data.<br><br>The same walkable road network used for buffer creation should be used here. "Walkable" road network means roads where pedestrians are prohibited are removed from the network before intersections are identified and counted. | | |
| **Details:** | **Please respond to the questions below:** | Excluded/ NO | Included/ YES |
| | | | |
| 78. Did you use the private recreation definitions provided above? | | | |
| a. If the definition you used differed from the one provided | | | |

| | | |
|---|---|---|
| above in any way? Please describe below: | | |
| 79. *If private recreation locations are represented by polygons (rather than points) then there are different methods of handling polygons that intersect and partially overlap participants' buffers. Please indicate which one of these procedures best describes the method you used:* | | |
| a. A private recreation parcel was included in the count of private recreation if a private recreation parcel's centroid was contained within the buffer polygon (**acceptable**). | | |
| b. A private recreation parcel was included in the count of private recreation if any portion of a private recreation-parcel polygon intersected the buffer polygon (**recommended**). | | |
| c. We used another method of handling the partial overlap of private recreation land use on buffer polygons (**speculative**). Please describe below: | | |
| 80. What unit is the distance measurement in? Please provide below: | | |
| 81. Was the same walkable road network used for buffer creation also used to determine the distance to the nearest private recreation location? If a different road network was used how was it different? What was the reason for using a different road network? Please describe below: | | |
| 82. Did you use parcel data in the count of recreation facilities? | | |
| 83. Did you check the accuracy of parcel data against phone books or other data sources in a sample? | | |

## Included in Private Recreation Facilities

Amusement places (with known physical activity facility)
Arcades (with known physical activity facility)
Baseball
Basketball
Batting cages
Dance; Dance Companies; Dance - Instruction- Ballet, Tap; Dance - Instruction - Ballroom
Gymnastics; Gymnastics - Instruction; Gymnastics/Dance
Health club; Health club/Martial Arts; Health club/pool; Health club/tennis - private
Hockey rinks
Indoor rock climbing
Kayaking
Lasertag
Martial arts
Paintball; Paintball games and supplies
Racquetball courts - private; Racquetball courts - public
Soccer; Soccer field
Swimming pool - private; swimming pool - public
Tennis - private; tennis - public
Video games - arcades (with known physical activity facility)
YMCA
Yoga; Yoga/Dance; Yoga/Health Club; Yoga/Martial arts
YWCA

# SECTION 13: PUBLIC PARKS

| | |
|---|---|
| **Aim:** | To develop a standardized definition of **public parks** to be compared across participants across countries.<br><br>To develop park count and park land area variables within or intersecting participants' buffers that can be compared across participants across countries. |
| **Tasks:** | 1a. Determine the number of park polygons (all sizes, and by park size) which are either contained by or intersect participants' buffers. See below for the list of size ranges (**all sizes required, by park size categories desired**).<br><br>1b. Determine the number of park polygons (all sizes, and by park size) which are completely contained by (that is, are wholly within) the participant buffers (**all sizes required, by park size categories desired**).<br><br>1c. Determine the number of trails that intersect participant buffers **(desired)**.<br><br>2a. Determine the sum of land area of park polygons (all sizes, and by park size categories) which are either contained by or intersect the participant buffers (**all sizes required, by park size categories desired**). Note: the park land area summed for this variable is the entire park area, not just the segment that intersects the buffer.<br><br>2b. Determine the sum of land area of park polygons (all sizes, and by park size) which are contained by (that is, are wholly within) the participant buffers (**all sizes required, by park size categories desired**). Note: the park land area summed for this variable included the entire park area. |
| **Datasets:** | Participant buffers; park polygons, trail lines |
| **Definition:** | The following sources were used in the U.S. to enumerate the total list of parks in the study area: government supplied park lists (e.g. name, address, amenities), GIS shapefiles showing park boundaries, parcel data (indicating land uses including parks), Google maps, Thomas Guides, internet websites created by various entities, aerial photography, as well as in-field visits. We found it was necessary to use multiple sources. |

<table>
<tr><td></td><td colspan="3">The goal is for each country to use the best available source of information. What is consistent across the sites is the use of the best available data, rather than the source of the data.<br><br>The following criteria were used in the U.S. to determine if an area is a public park:<ul><li>Considered a public park by the government agency supplying the information. Government agency covers federal, state, county, municipalities, parks departments, planning departments, etc.</li><li>Considered a public park if a government agency maintains the park area.</li><li>Considered a park if it is physically accessible to the public (e.g. no fences blocking access, open entry points).</li><li>Beaches and wooded areas are considered parks if they function as public park areas.</li><li><u>Not</u> considered a park if it is: 1) maintained by a home owner’s association, 2) part of an apartment complex playground, 3) an unimproved open space with ‘no use designated’, 4) or a proposed park, or a school, religious facility, or golf course.</li></ul>A trail is a walking/hiking/bicycle facility that is not part of a roadway and is <u>not</u> a sidewalk.  The facility can be adjacent to a road, but can’t be connected to a road (e.g. a bike lane which is physically connected to the road is not a trail; a paved path (not a sidewalk) that is adjacent to a road, but separated from the road via a grass buffer is a trail.).</td></tr>
<tr><td>Sidenotes:</td><td colspan="3">Count and area variables are created for each of the following seven size categories:<ul><li>All sizes</li><li><=0.25 acres land area</li><li>>0.25 to <=1 acres land area</li><li>>1 to <=5 acres land area</li><li>>5 to <=10 acres land area</li><li>>10 acres to <=50 acres  land area</li><li>>50 acres land area</li><li>[Where 1 acre = 4,046.86 sq meters]</li></ul></td></tr>
<tr><td>Details:</td><td>Please respond to the questions below:</td><td>Excluded /NO</td><td>Included /YES</td></tr>
<tr><td colspan="2">1. Did your definition of park differ from the one provided above in any way?  Please describe below:</td><td></td><td></td></tr>
<tr><td colspan="2">h. Did parks include public and free locations only?</td><td></td><td></td></tr>
</table>

| | | |
|---|---|---|
| i. Did parks include areas maintained by government agencies? | | |
| j. Were unimproved open spaces excluded (e.g. green spaces without improvements)? | | |
| k. Were unique locations included based upon your regional or cultural understanding of park space? Please describe below: | | |
| 2. Did you count the number of park polygons that are either contained by or intersect participants' buffers? | | |
| a. Did you calculate one overall count that includes parks of all sizes **(required)**? | | |
| b. Did you calculate counts for the specific size categories shown above **(desired)**? If not, what park sizes do counts exist for? Please list below: | | |
| 3. Did you count the number of park polygons that are contained by (wholly within) each buffer? | | |
| a. Did you calculate one overall count that includes parks of all sizes **(required)**? | | |
| b. Did you calculate counts for the specific size categories shown above **(desired)**? If not, what park sizes do counts exist for? Please list below: | | |
| 4. Did you count the number of trails that intersect each buffer **(desired)**? | | |
| 5. Did you sum the land area of park polygons that are either contained by or intersect participants' buffers. | | |
| a. Did you calculate the sum of park area for parks of all sizes **(required)**? | | |

| | | |
|---|---|---|
| b. Did you calculate the sum of park area for the size categories shown above **(desired)**? If not, what park sizes do area sums exist for? Please list below: | | |
| 6. Did you sum the land area of park polygons that are contained by (are wholly within) the participant buffers. | | |
| a. Did you calculate the sum of park area for parks of all sizes **(required)**? | | |
| b. Did you calculate the sum of park area for the size categories shown above **(desired)**? If not, what park sizes do area sums exist for? Please list below: | | |
| 7. What units (e.g. acres, square meter) do your land area variables use? | | |
| 8. What sources did you use to enumerate the complete set of public parks in the study area? Please provide a general list (e.g. local government, business directories, areal photography) of all sources. We would like to compare across countries. | | |
| 9. Did you develop any other measures of park or trail access (e.g. total length of trails in buffer)? If so, please list below and provide a complete description of the measure. | | |

# SECTION 13 CONTINUED: PUBLIC PARKS (DISTANCE TO NEAREST)

| | |
|---|---|
| **Aim:** | To develop a standardized definition of distance from homes to **public parks** **and** **trails** to be compared across participants across countries.<br><br>To develop a variable that indicates the street network distance from each participant's home address to the nearest park (**required**), within different size categories (**desired**). To be done for each size range of parks shown below.<br><br>To develop a variable that indicates the street network distance from each participant's home address to the nearest trail (**required**). |
| **Tasks:** | Calculate distance to the nearest park to each participant's home address.<br><br>Calculate distance to the nearest trail to each participant's home address. |
| **Datasets:** | Home address location; walkable road network, park polygons, trail lines |
| **Definition:** | See definition of park above.<br><br>A variable showing the network distance from the participant's home to the nearest park is created for each of the following seven size categories:<br>• All sizes<br>• <=0.25 acres land area<br>• >0.25 to <=1 acres land area<br>• >1 to <=5 acres land area<br>• >5 to <=10 acres land area<br>• >10 acres to <=50 acres land area<br>• >50 acres land area<br>[Where 1 acre = 4,046.86 sq meters] |
| **Sidenotes:** | A walkable road network should be used for all distance estimates. See intersection density for a description of a walkable road network.<br><br>The distance measures in the U.S. were calculated using the network analyst extension in the ESRI ArcGIS software. Determining distances in this way requires an origin point and a destination point, both of which need to be on the walkable road network. The |

| | | | |
|---|---|---|---|
| | participant's home address is the origin point; the destination is a park represented by a polygon.<br><br>It is therefore necessary to represent the park polygon as a point on the walkable road network. Ideally each park polygon would have points associated with it indicating the entrances from the road network to the park. In the U.S. we did not have that. Instead park polygons were represented as point on the road network by buffering the polygon by 50 feet. Points representing parks were created at the locations where the buffered park polygon intersected with the road network. *This method typically this results in multiple points for a single park. These points were accepted as representative of where someone could enter the park.* In some cases parks were more than 50 feet from the road network. These cases were investigated to determine if it was possible to enter the park from the road, albeit at a distance greater than 50 feet. Depending on the results of the review, points on the road network were created for the parks or the parks were excluded from this task. | | |
| **Details:** | **Please respond to the questions below:** | Excluded /NO | Included /YES |
| 10. Did you calculate the walkable road network based distances for the park size categories shown above (**desired**)? | | | |
| a. Did you use a different method (e.g. crow-fly) for determining the distance (**required**). If so, describe the method below: | | | |
| b. Did you calculate nearest distance that includes parks of all sizes **(required)**? | | | |
| c. Did you calculate nearest distance for the specific size categories shown above **(desired)**? | | | |
| d. If different park size categories were used what are they? Please describe below: | | | |
| 11. Did you calculate the walkable road network distance for trails (**desired**)? | | | |
| 12. What units are your distance measurements in? | | | |
| 13. Did you use the same method as described above to represent park polygons as points on the road network? If not, please describe the method used below: | | | |

| | | |
|---|---|---|
| | | |
| 14. Did you use the same criteria to designate public parks as are indicated above? If not, what criteria were used? | | |
| 15. When calculating ‘distance to nearest’ variables, did you use a cut-off distance? For example, if a park was not found within 24 km, did you set the GIS software to stop looking? If so, please tell us the distance that you used? | | |

# SECTION 14

IPEN 500m Street Network Buffers Variable Naming Convention

INTERNATIONAL PHYSICAL ACTIVITY AND THE ENVIRONMENT NETWORK

# IPEN: Residential Land Use

## 500m Street Network Buffer

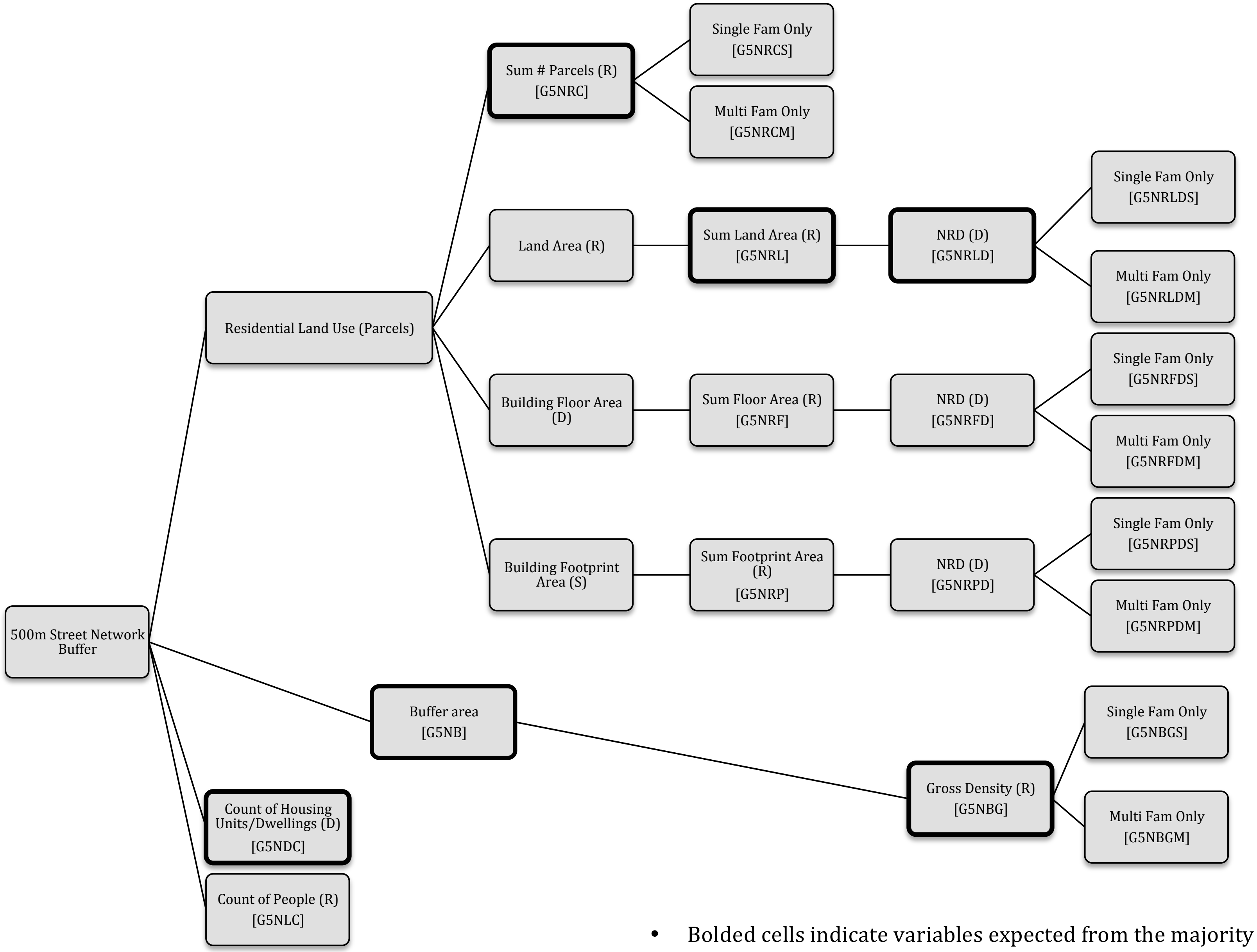


- Bolded cells indicate variables expected from the majority of countries.
- Letters in parentheses indicate whether variable is required (R), desired (D), or speculative (S).
- Alphanumeric values in [ ] provide variable names to use in GIS datafiles before [illegible]

4/10/12

# IPEN: Retail Land Use

## 500m Street Network Buffer

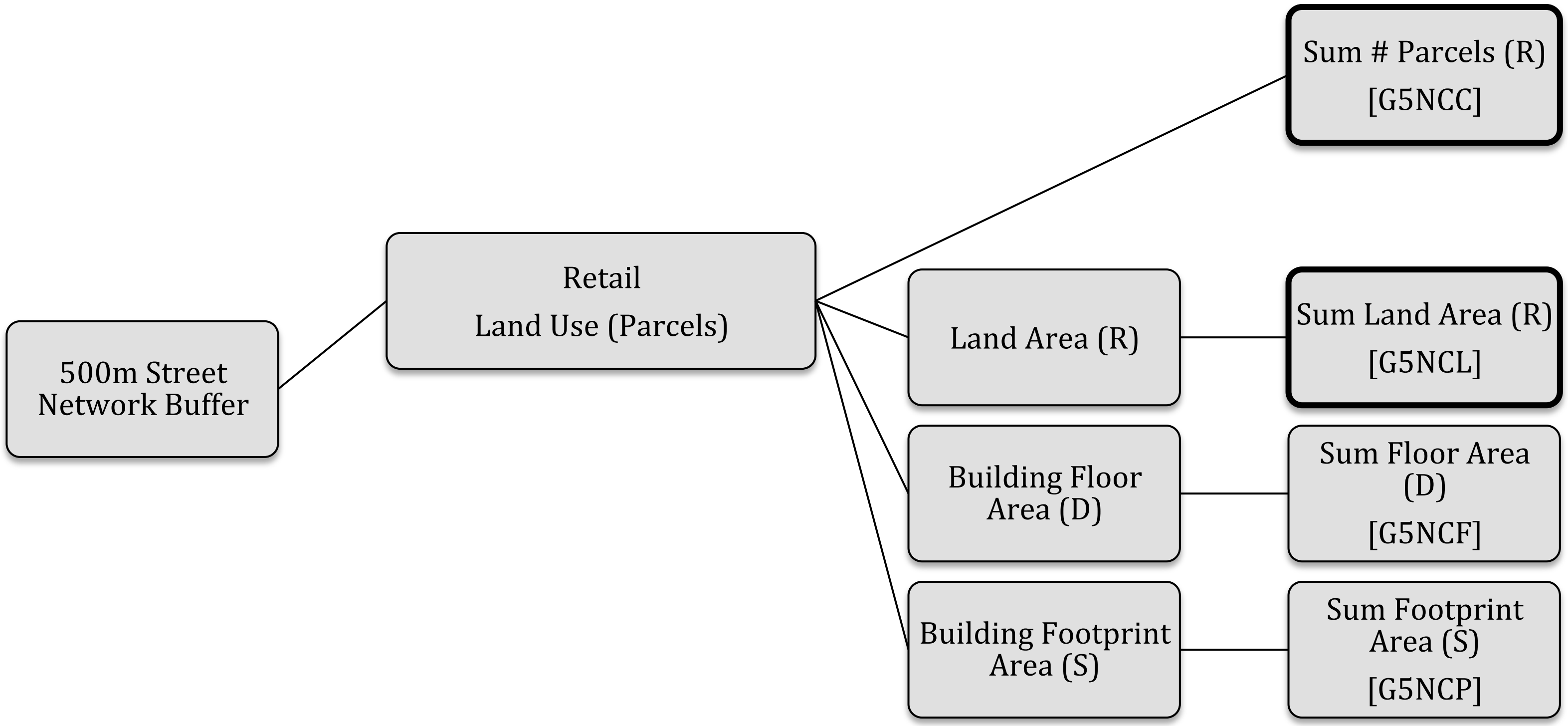


- Bolded cells indicate variables expected from the majority of countries.
- Letters in parentheses indicate whether variable is required (R) or desired (D).
- Alphanumeric values in [ ] provide variable names to use in GIS datafiles before sending to the coordinating center.

4/10/12

# IPEN: Civic and Institutional Land Use

## 500m Street Network Buffer

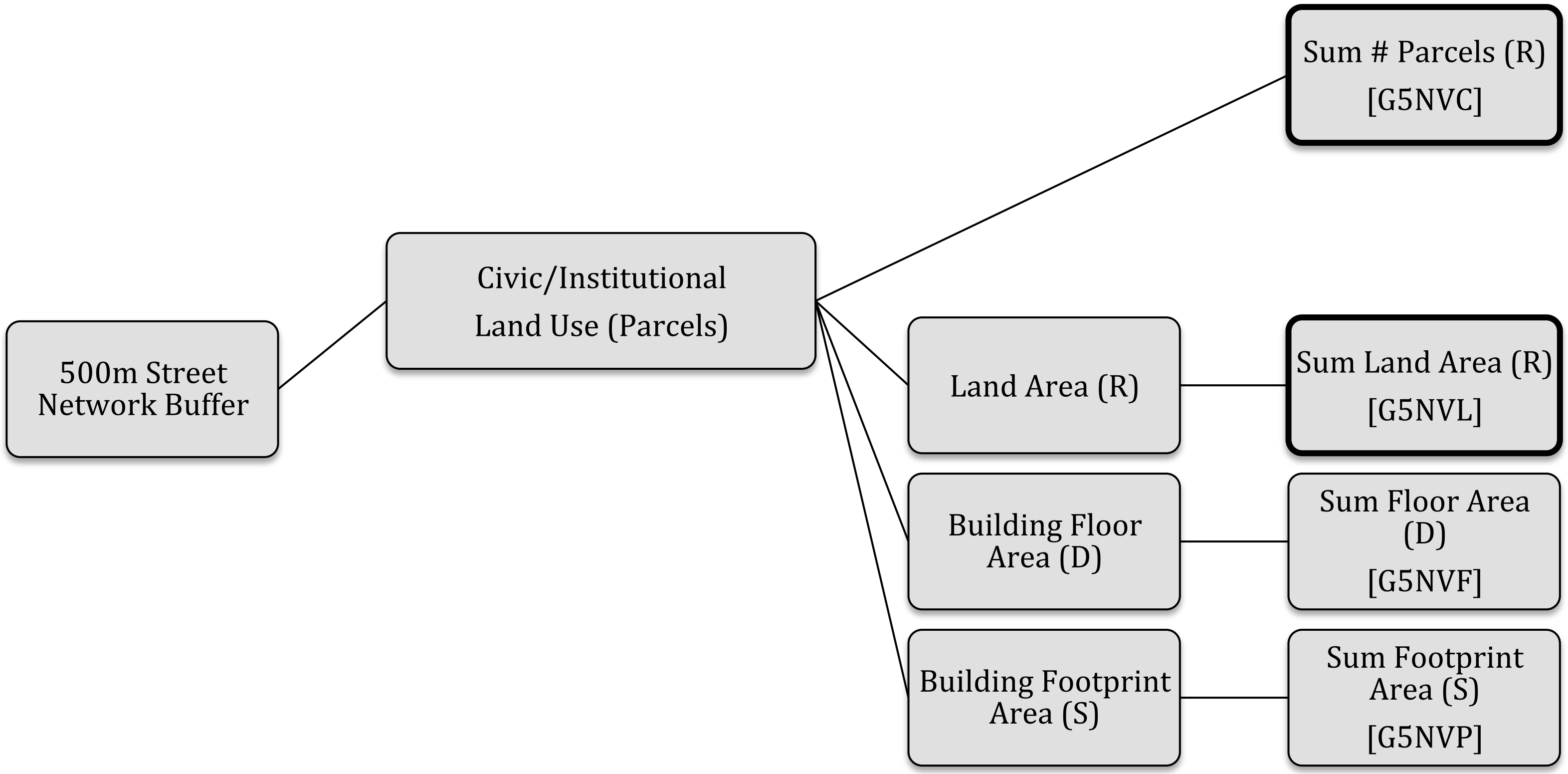


- Bolded cells indicate variables expected from the majority of countries.
- Letters in parentheses indicate whether variable is required (R) or desired (D).
- Alphanumeric values in [ ] provide variable names to use in GIS datafiles before sending to the coordinating center.

4/10/12

# IPEN: Entertainment Land Use

## 500m Street Network Buffer

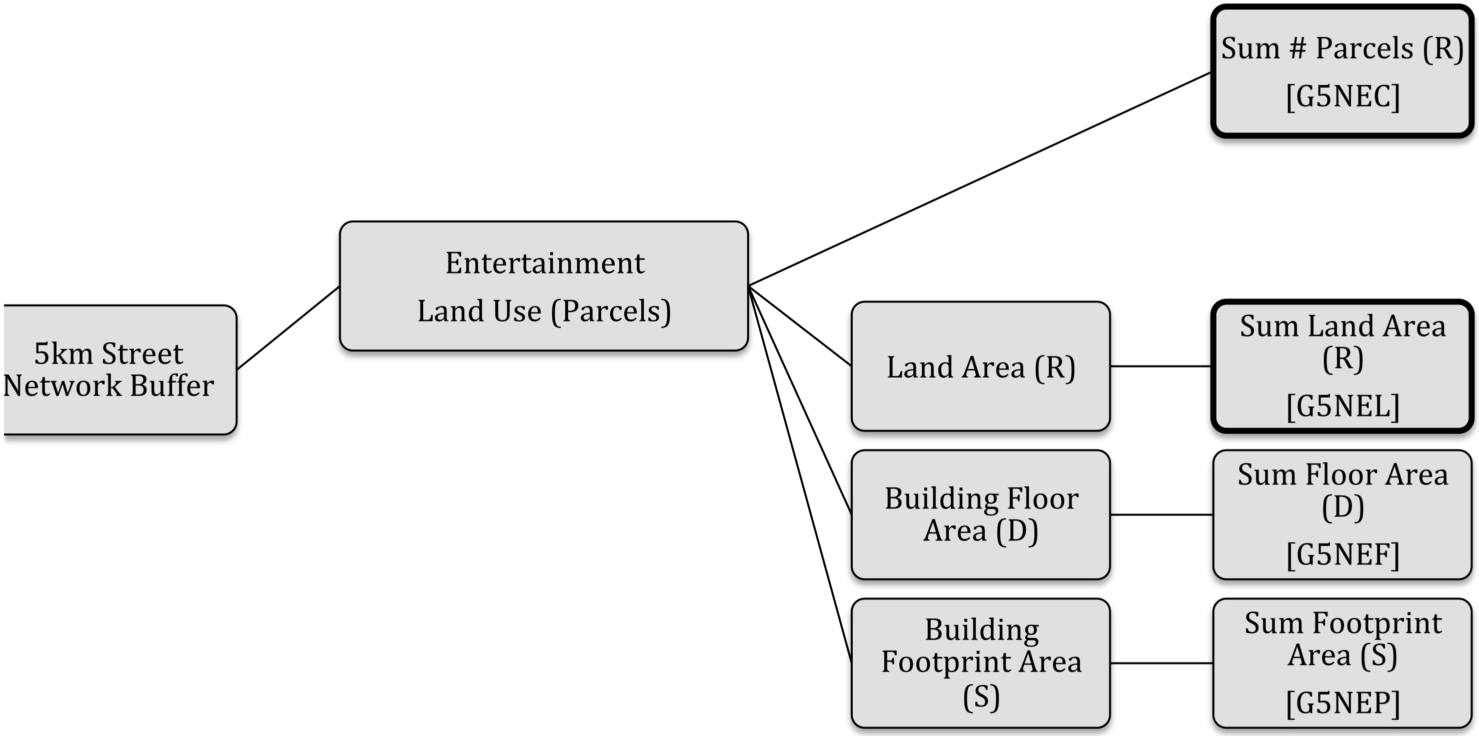


- Bolded cells indicate variables expected from the majority of countries.
- Letters in parentheses indicate whether variable is required (R) or desired (D).
- Alphanumeric values in [ ] provide variable names to use in GIS datafiles before sending to the coordinating center.

4/10/12

# IPEN: Private and Public Recreation Land Use

## 500m Street Network Buffer

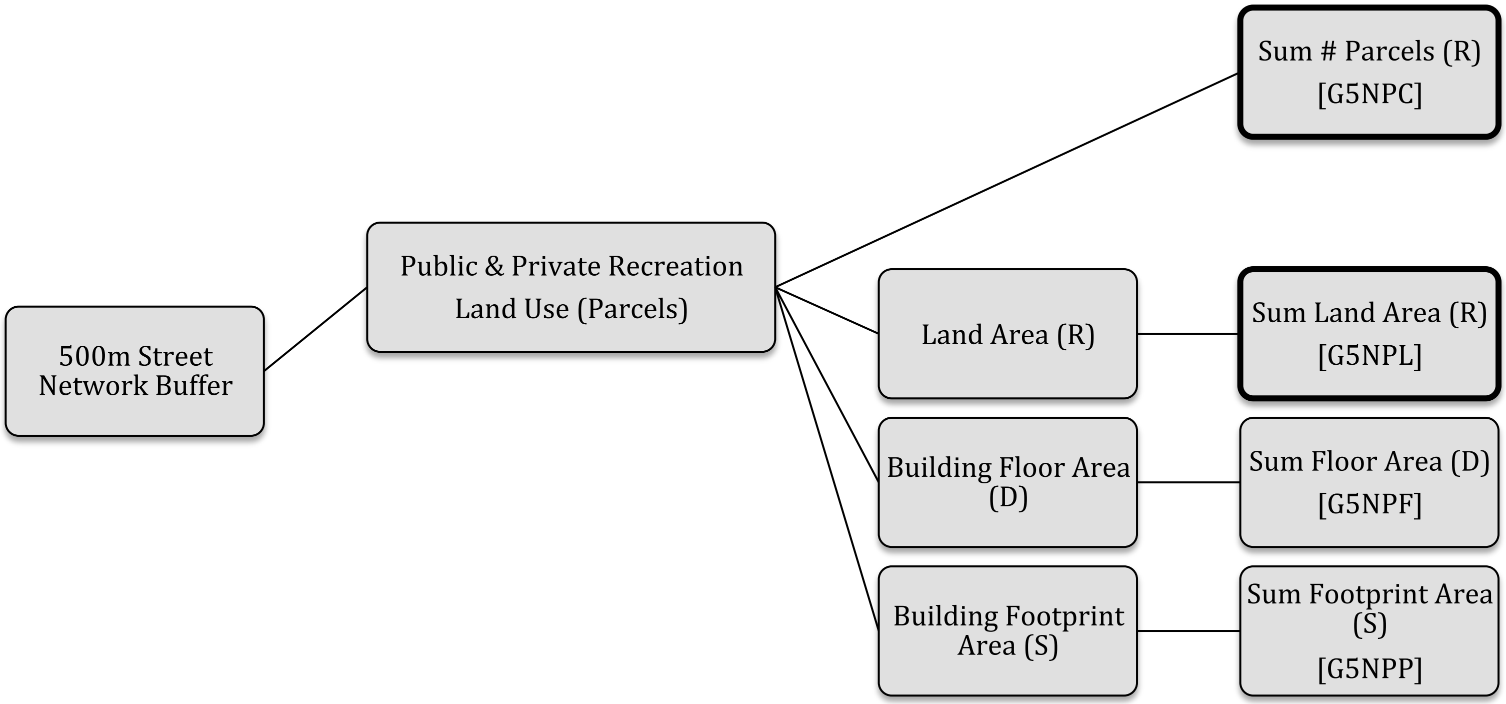


- Bolded cells indicate variables expected from the majority of countries.
- Letters in parentheses indicate whether variable is required (R) or desired (D).
- Alphanumeric values in [ ] provide variable names to use in GIS datafiles before sending to the coordinating center.

4/10/12

IPEN: Food and Restaurant Land Use

500 Street Network Buffer

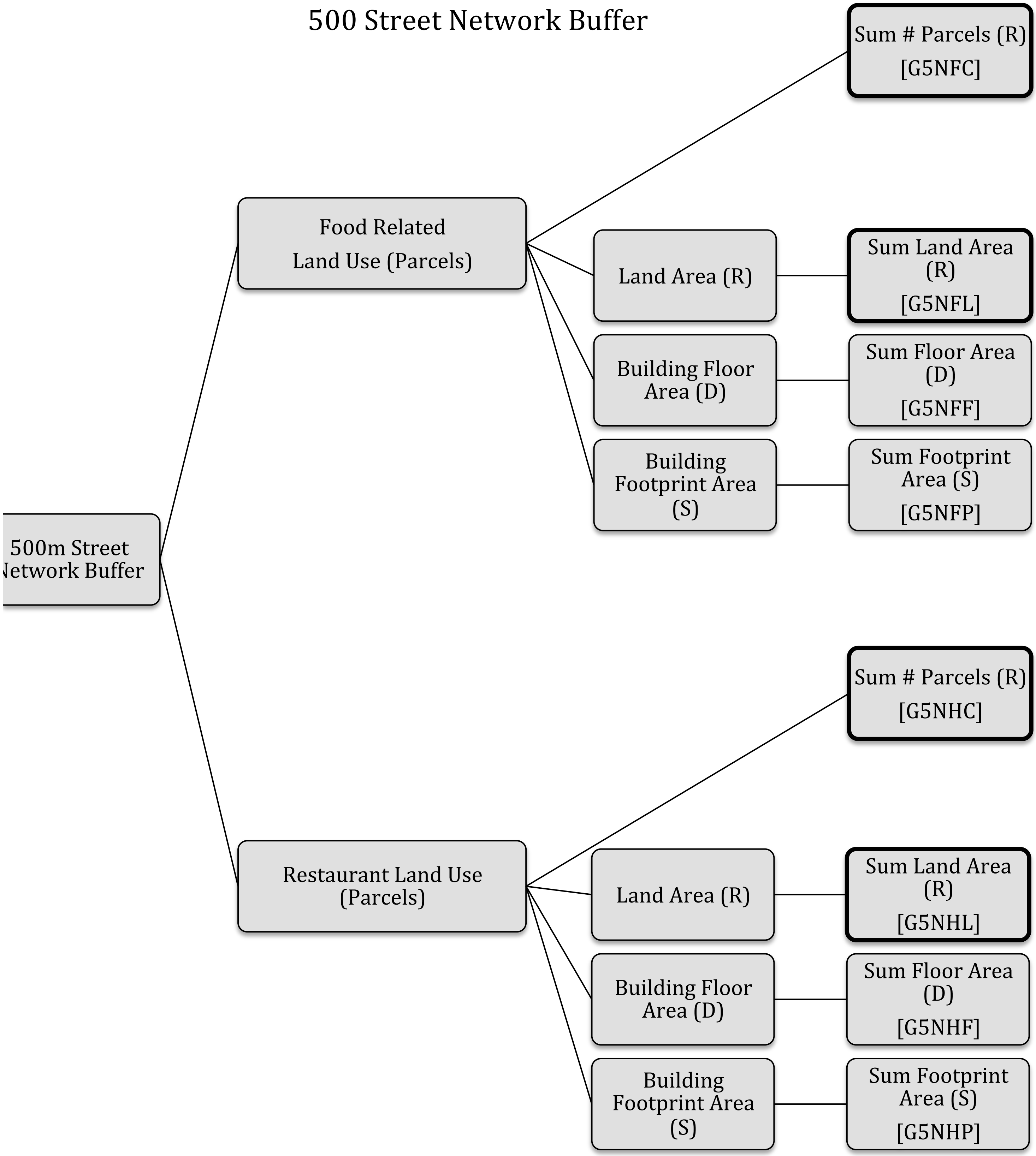


- Bolded cells indicate variables expected from the majority of countries.
- Letters in parentheses indicate whether variable is required (R) or desired (D).
- Alphanumeric values in [ ] provide variable names to use in GIS datafiles before sending to the coordinating center.

# IPEN:  Intersections

## 500m Street Network Buffer

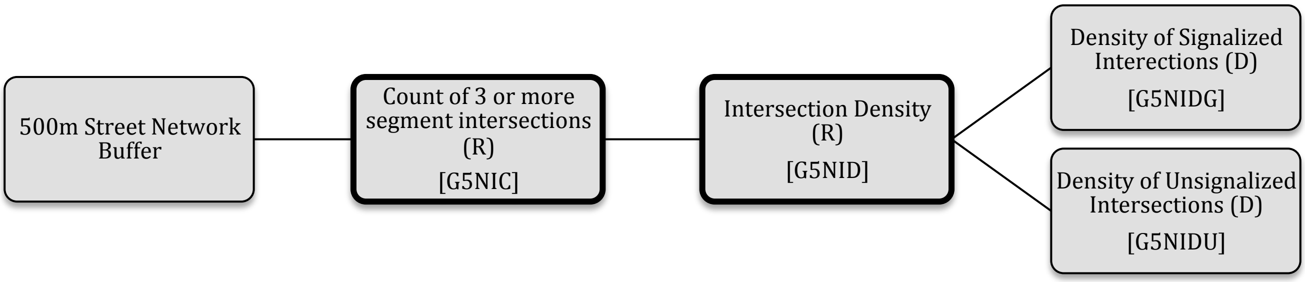


- Bolded cells indicate variables expected from the majority of countries.
- Letters in parentheses indicate whether variable is required (R) or desired (D).
- Alphanumeric values in [  ] provide variable names to use in GIS datafiles before sending to the coordinating center.

# IPEN: Public Transportation

# 500m Street Network Buffer

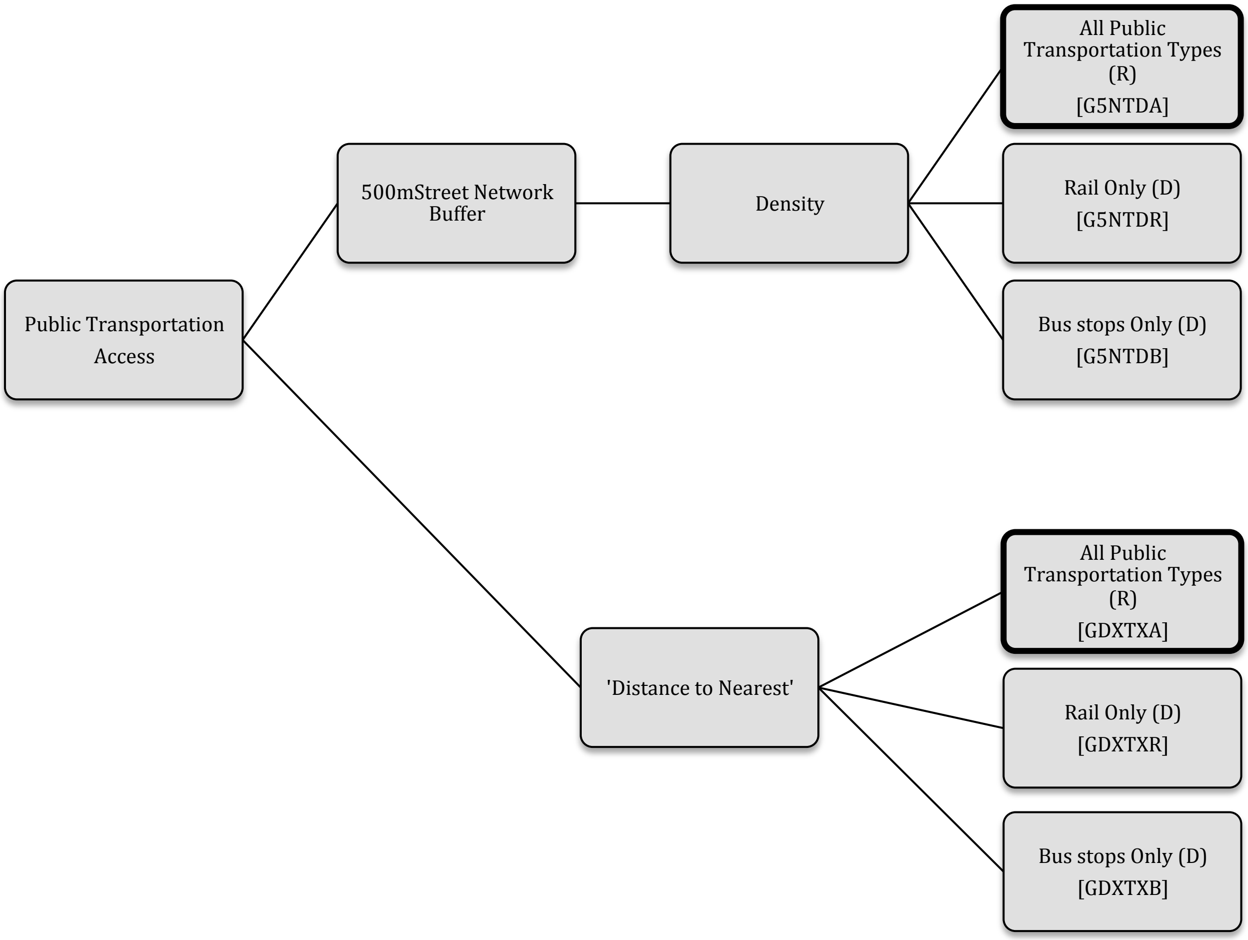


- Bolded cells indicate variables expected from the majority of countries.
- Letters in parentheses indicate whether variable is required (R) or desired (D).
- Alphanumeric values in [ ] provide variable names to use in GIS datafiles before sending to the coordinating center.

4/10/12

# IPEN:  Private Recreation Facility

## 500m Street Network Buffer

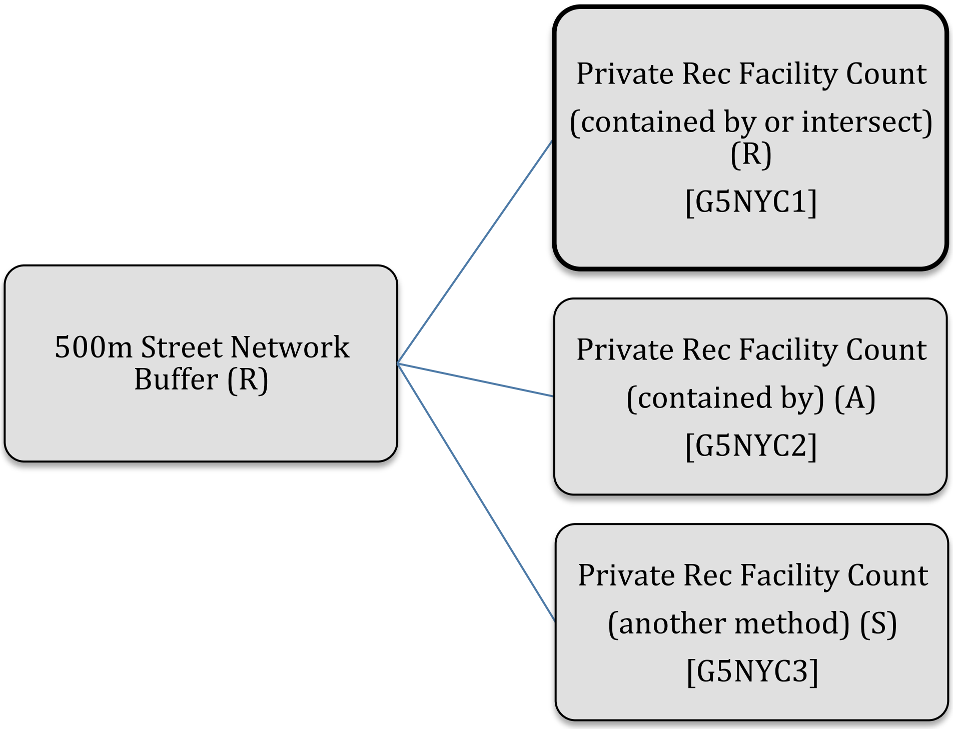


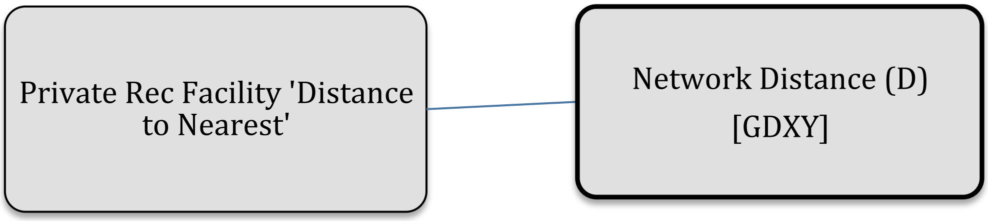


- Bolded cells indicate variables expected from the majority of countries.
- Letters in parentheses indicate whether variable is required (R) or desired (D).
- Alphanumeric values in [ ] provide variable names to use in GIS datafiles before sending to the coordinating center.

# IPEN: Public Parks

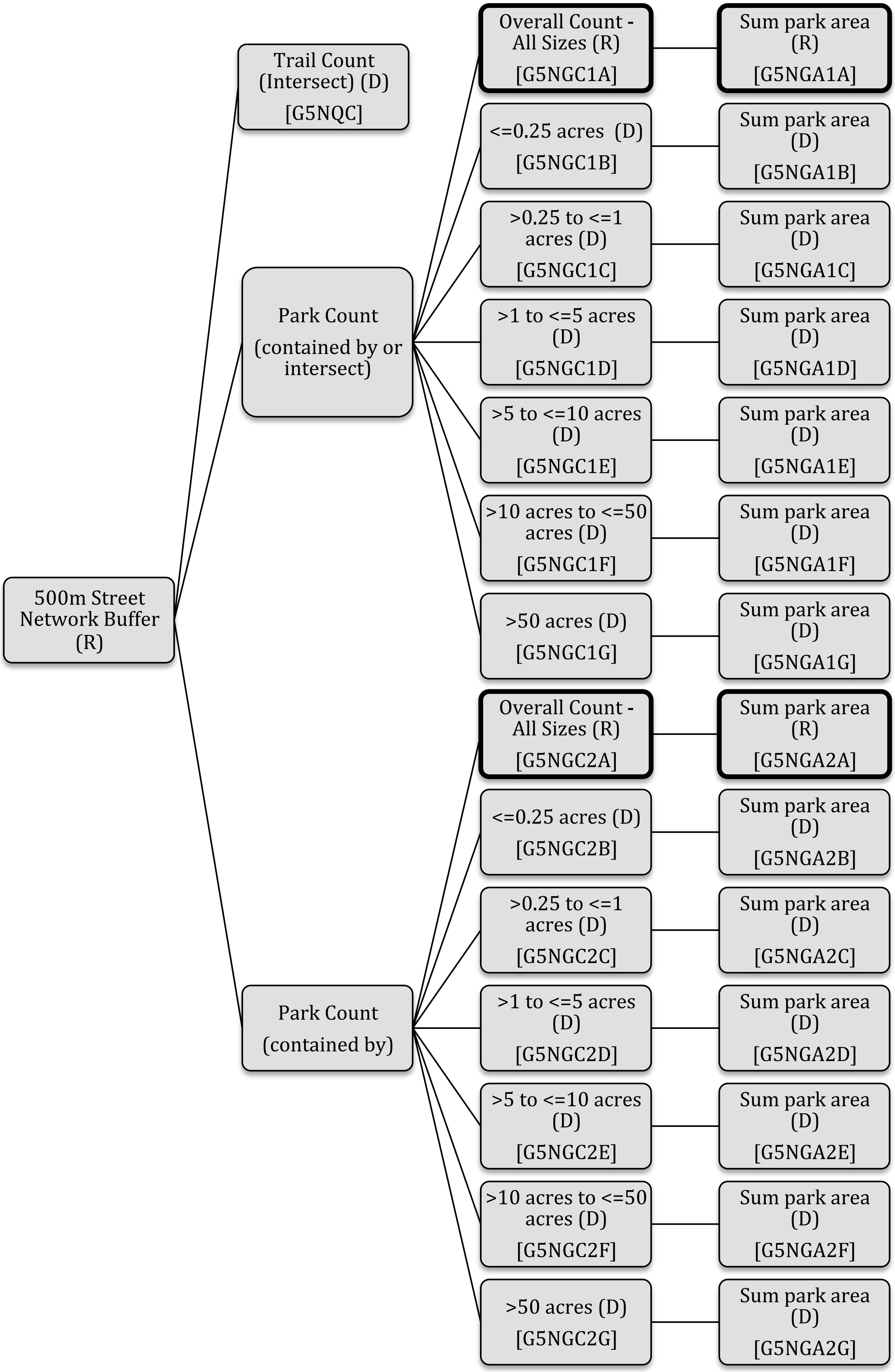


4/10/12

# SECTION 15

IPEN 1km Street Network Buffers Variable Naming Convention

# IPEN: Residential Land Use

## 1km Street Network Buffer

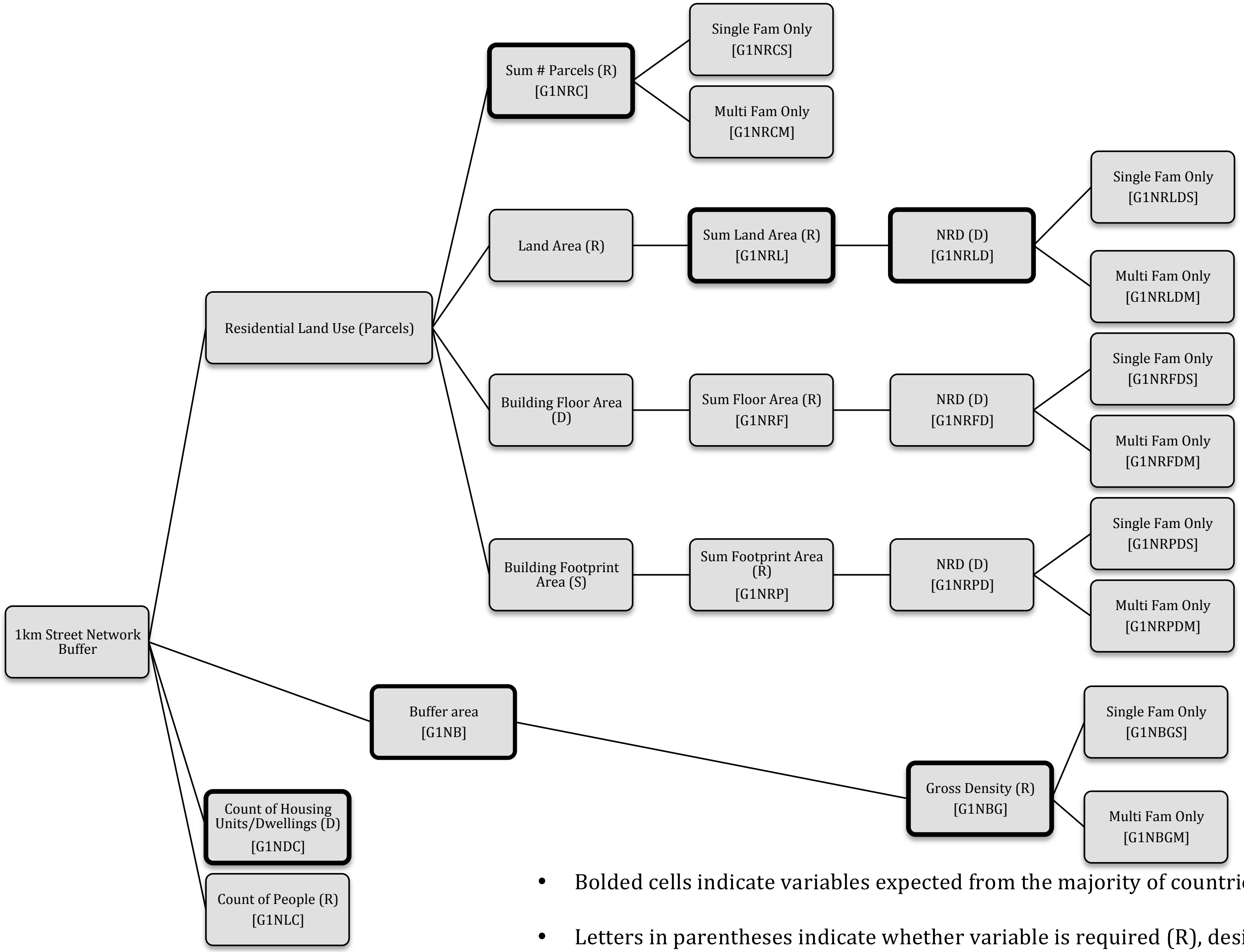


- Bolded cells indicate variables expected from the majority of countries.
- Letters in parentheses indicate whether variable is required (R), desired (D), or speculative (S).
- Alphanumeric values in [ ] provide variable names to use in GIS datafiles before sending to the coordinating center.

4/10/12

IPEN: Retail Land Use

1km Street Network Buffer

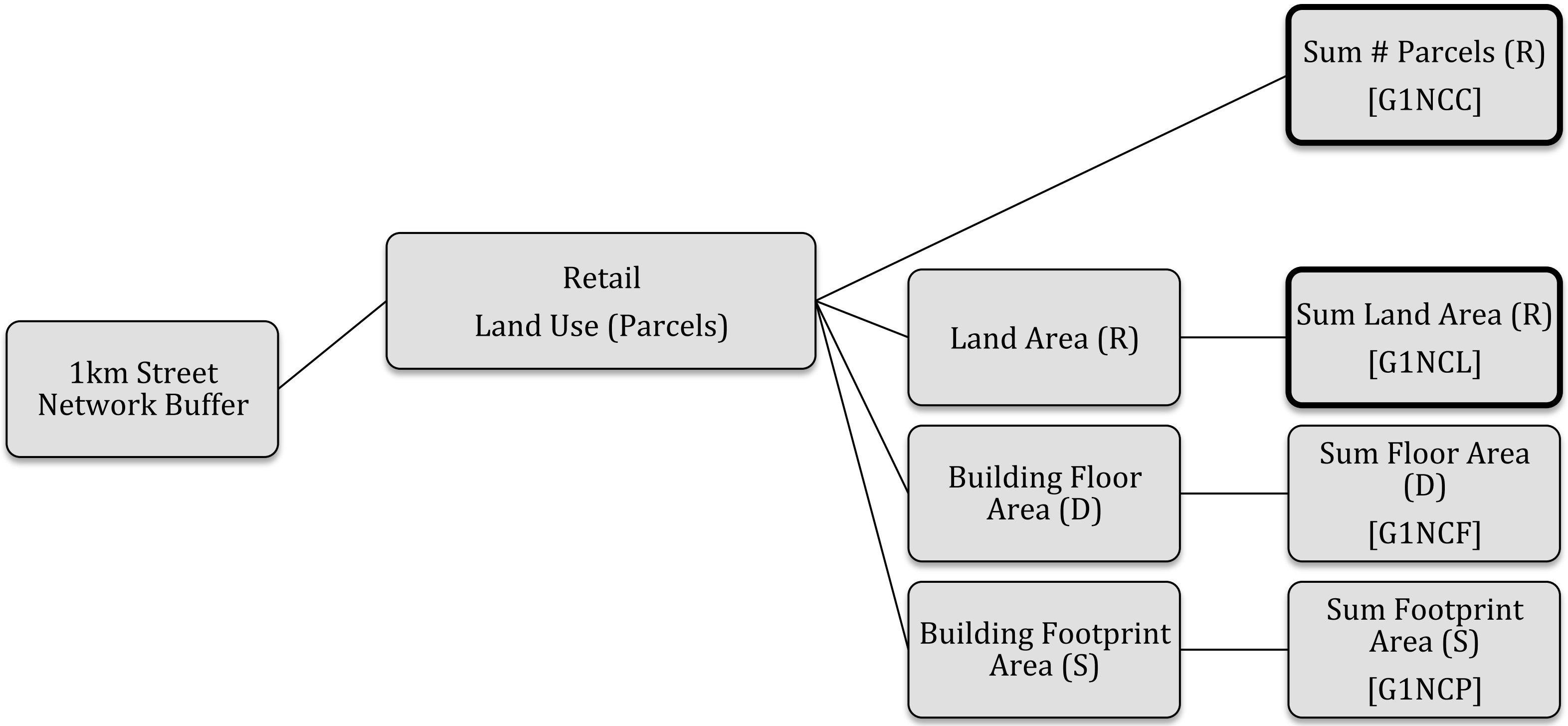


- Bolded cells indicate variables expected from the majority of countries.
- Letters in parentheses indicate whether variable is required (R) or desired (D).
- Alphanumeric values in [ ] provide variable names to use in GIS datafiles before sending to the coordinating center.

4/10/12

# IPEN: Civic and Institutional Land Use

## 1km Street Network Buffer

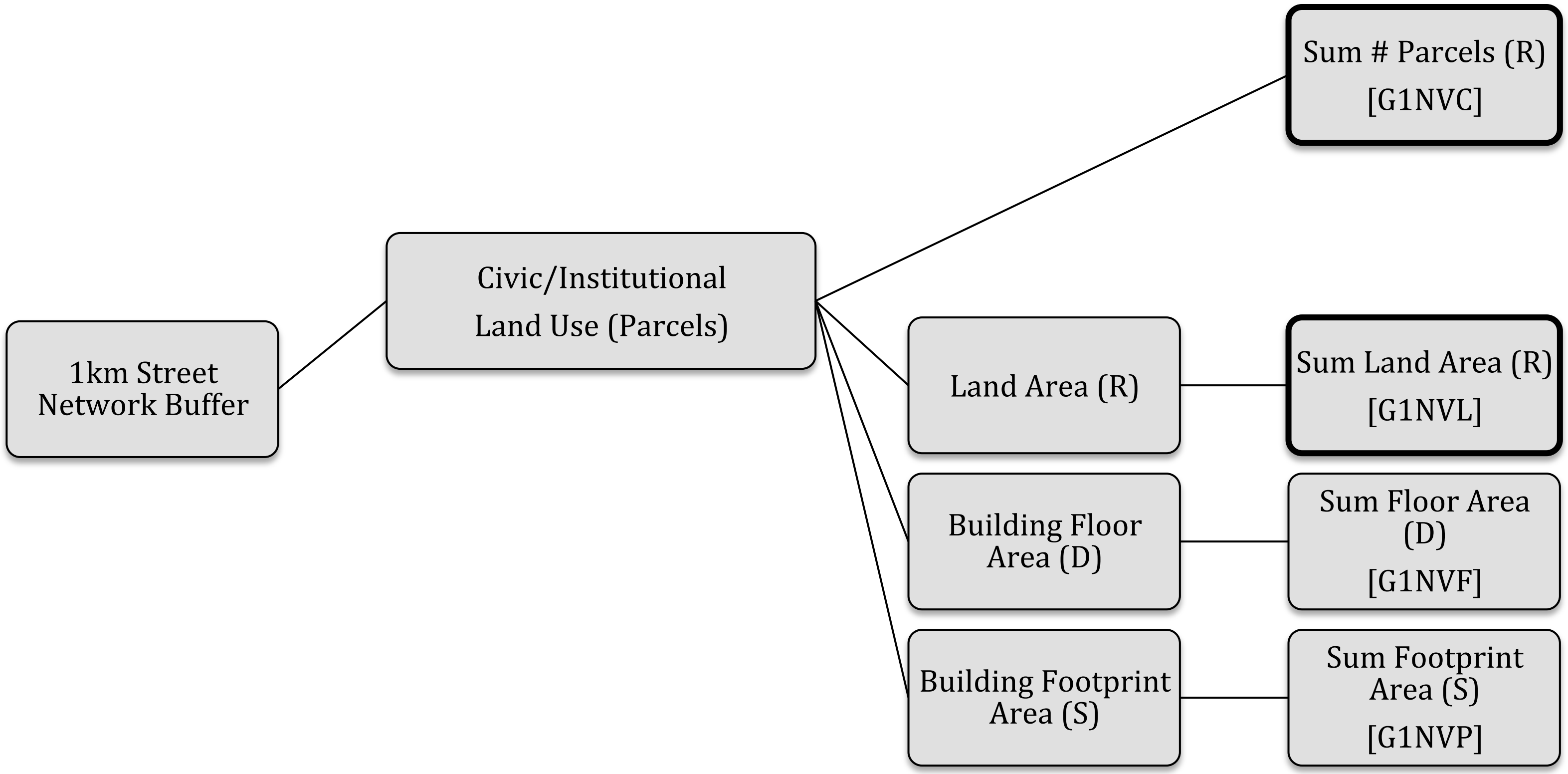


- Bolded cells indicate variables expected from the majority of countries.
- Letters in parentheses indicate whether variable is required (R) or desired (D).
- Alphanumeric values in [ ] provide variable names to use in GIS datafiles before sending to the coordinating center.

4/10/12

# IPEN:  Entertainment Land Use

## 1km Street Network Buffer

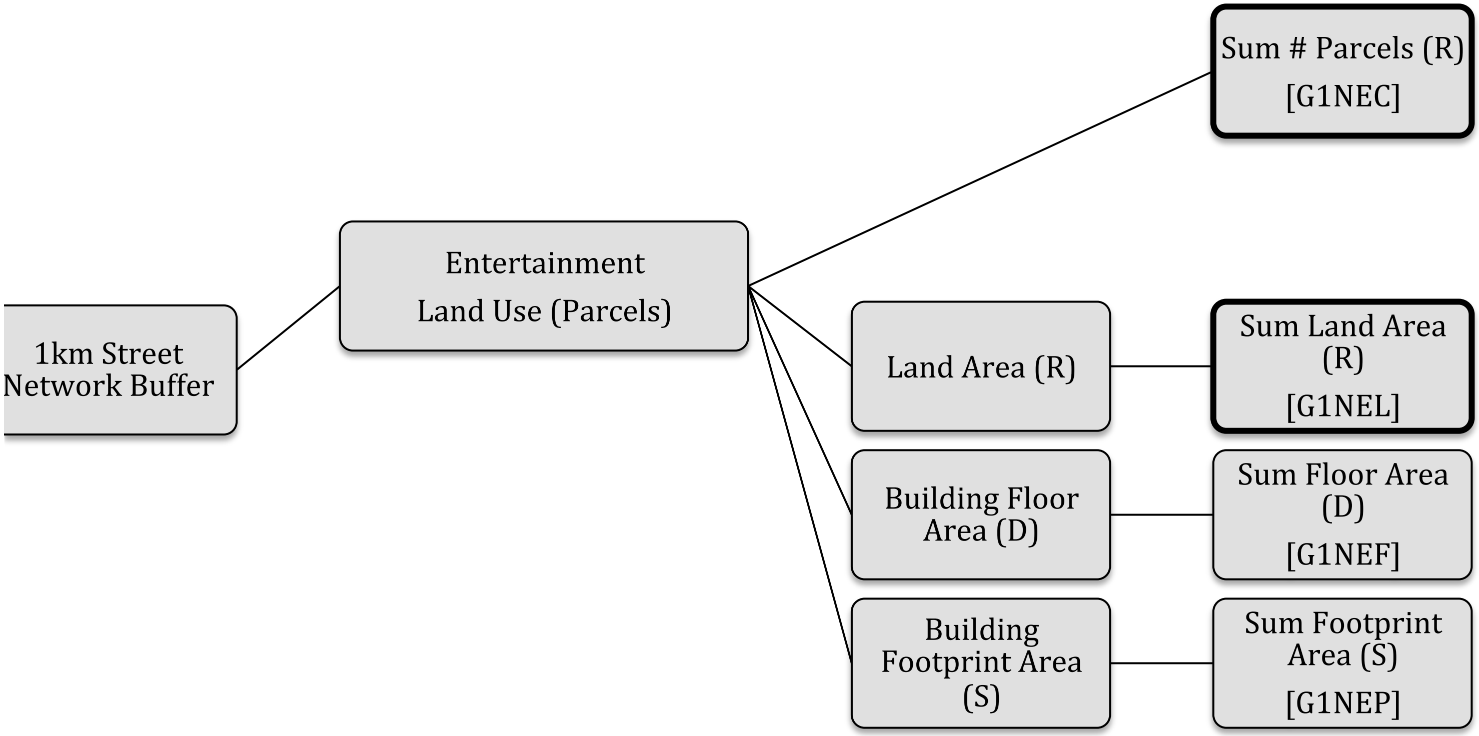


- Bolded cells indicate variables expected from the majority of countries.
- Letters in parentheses indicate whether variable is required (R) or desired (D).
- Alphanumeric values in [  ]  provide variable names to use in GIS datafiles before sending to the coordinating center.

4/10/12

# IPEN: Private and Public Recreation Land Use

## 1km Street Network Buffer

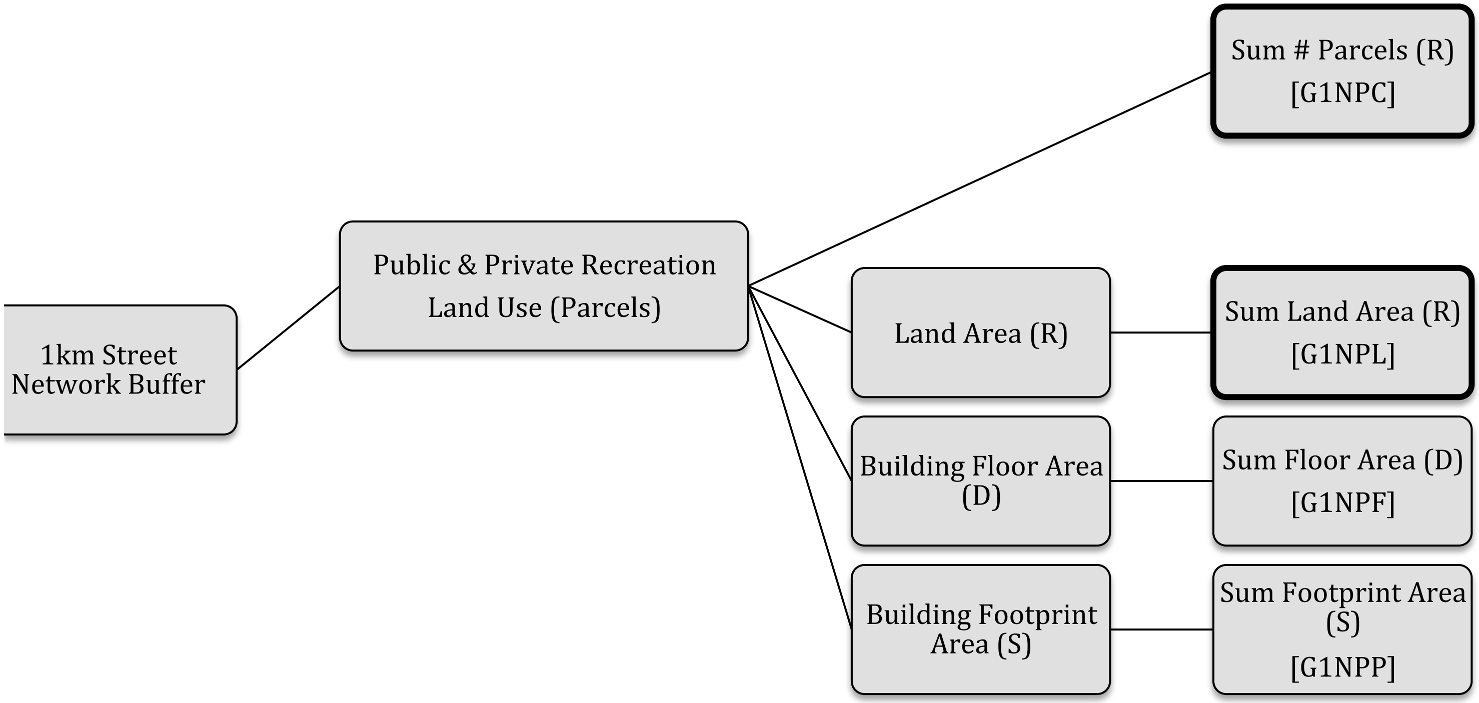


- Bolded cells indicate variables expected from the majority of countries.
- Letters in parentheses indicate whether variable is required (R) or desired (D).
- Alphanumeric values in [ ] provide variable names to use in GIS datafiles before sending to the coordinating center.

4/10/12

# IPEN: Food and Restaurant Land Use

## 1km Street Network Buffer

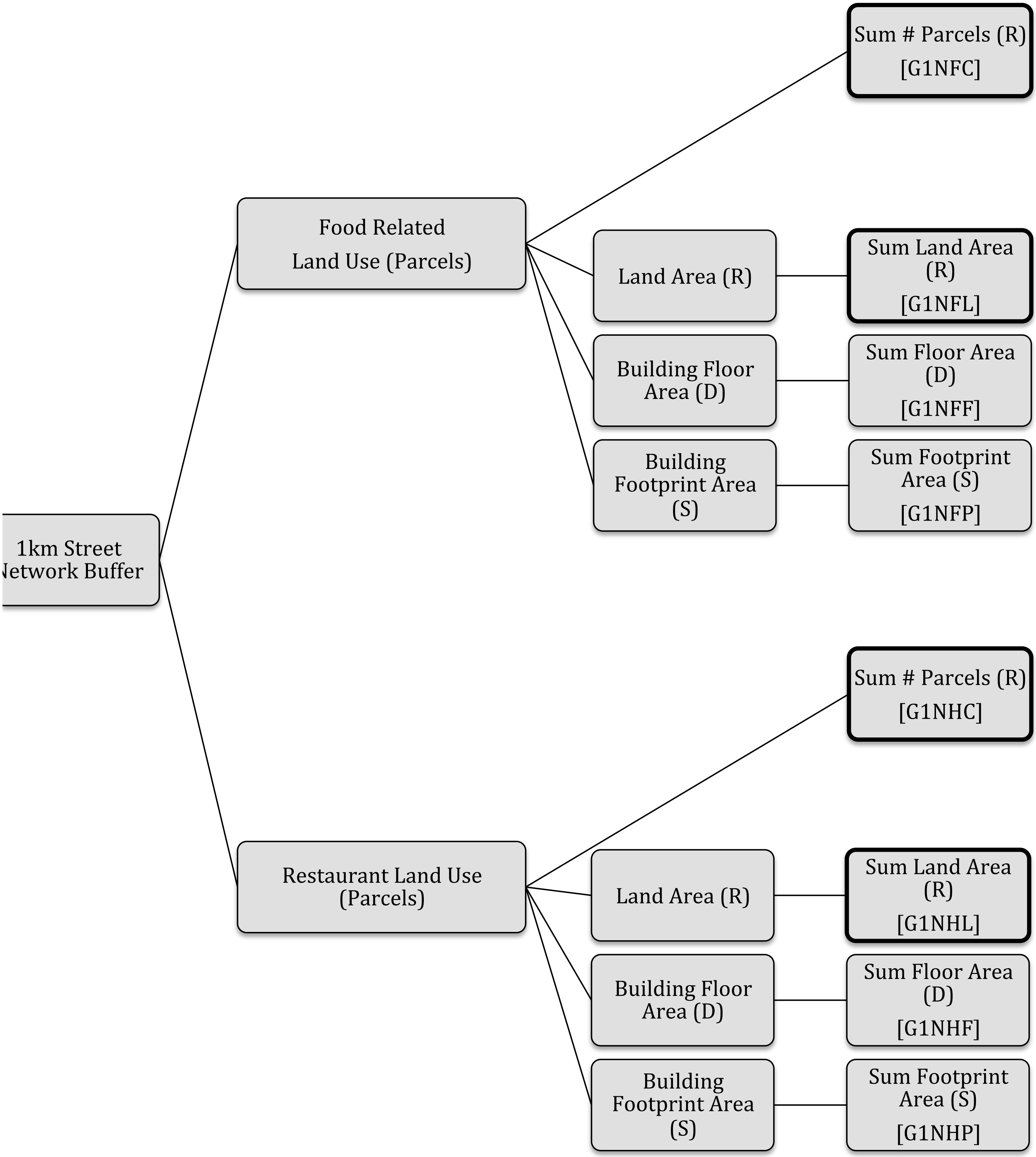


- Bolded cells indicate variables expected from the majority of countries.
- Letters in parentheses indicate whether variable is required (R) or desired (D).
- Alphanumeric values in [ ] provide variable names to use in GIS datafiles before sending to the coordinating center.

# IPEN: Intersections

## 1km Street Network Buffer

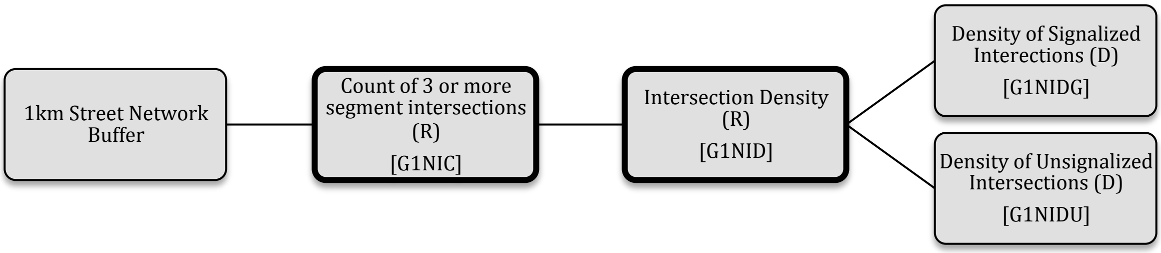


- Bolded cells indicate variables expected from the majority of countries.
- Letters in parentheses indicate whether variable is required (R) or desired (D).
- Alphanumeric values in [ ] provide variable names to use in GIS datafiles before sending to the coordinating center.

# IPEN: Public Transportation

# 1km Street Network Buffer

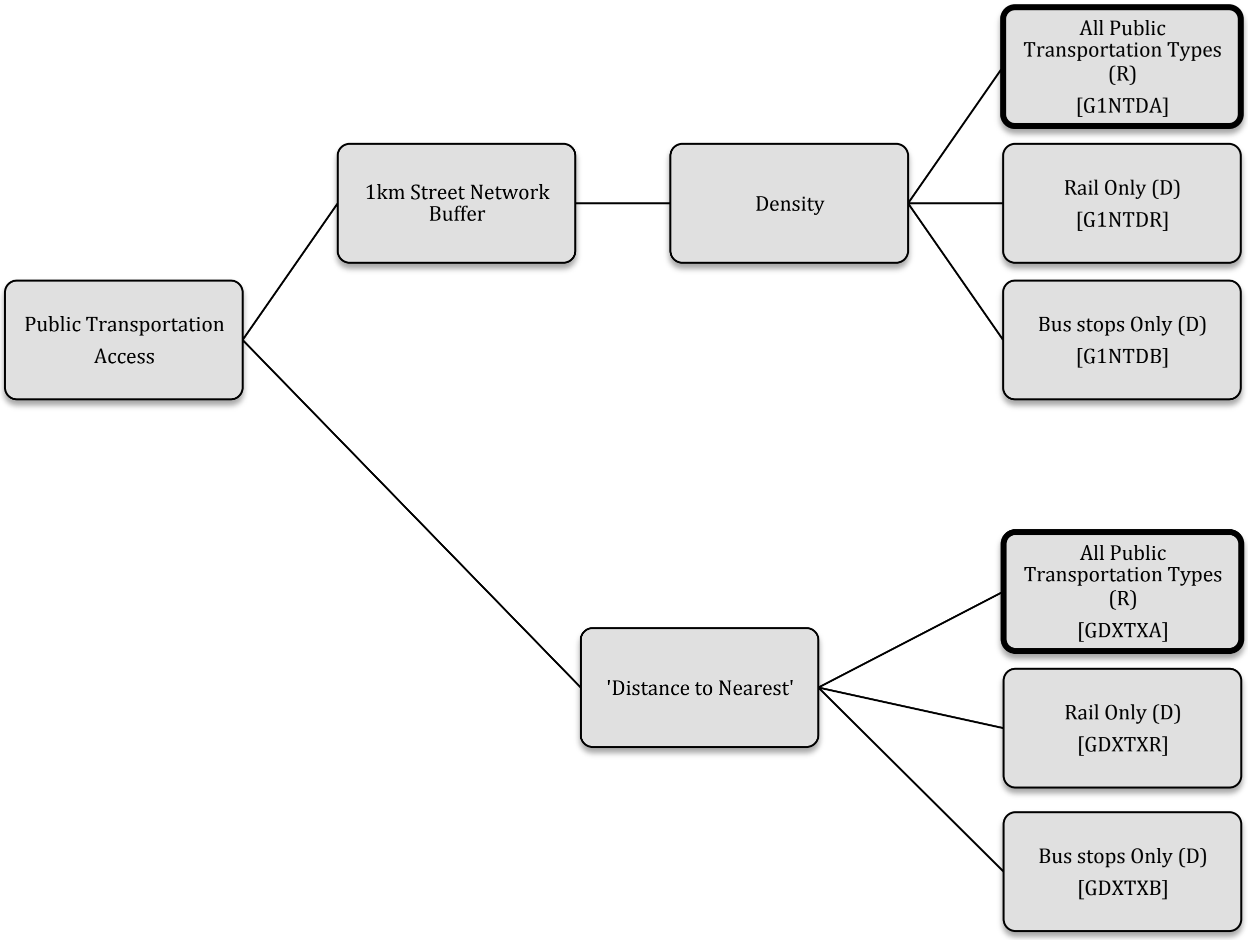


- Bolded cells indicate variables expected from the majority of countries.
- Letters in parentheses indicate whether variable is required (R) or desired (D).
- Alphanumeric values in [ ] provide variable names to use in GIS datafiles before sending to the coordinating center.

4/10/12

# IPEN: Private Recreation Facility

## 1km Street Network Buffer

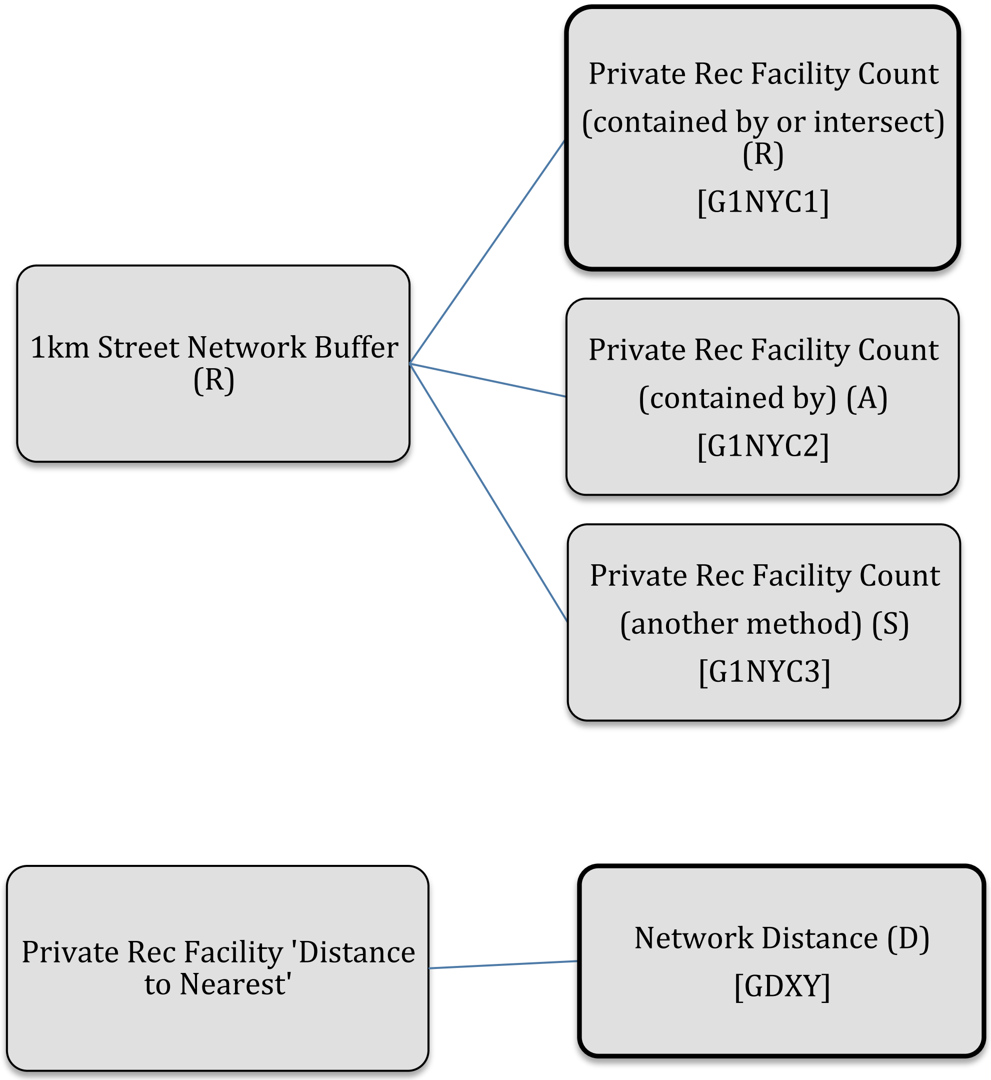


- Bolded cells indicate variables expected from the majority of countries.
- Letters in parentheses indicate whether variable is required (R) or desired (D).
- Alphanumeric values in [ ] provide variable names to use in GIS datafiles before sending to the coordinating center.

4/10/12

# IPEN: Public Parks

## 1km Street Network Buffer

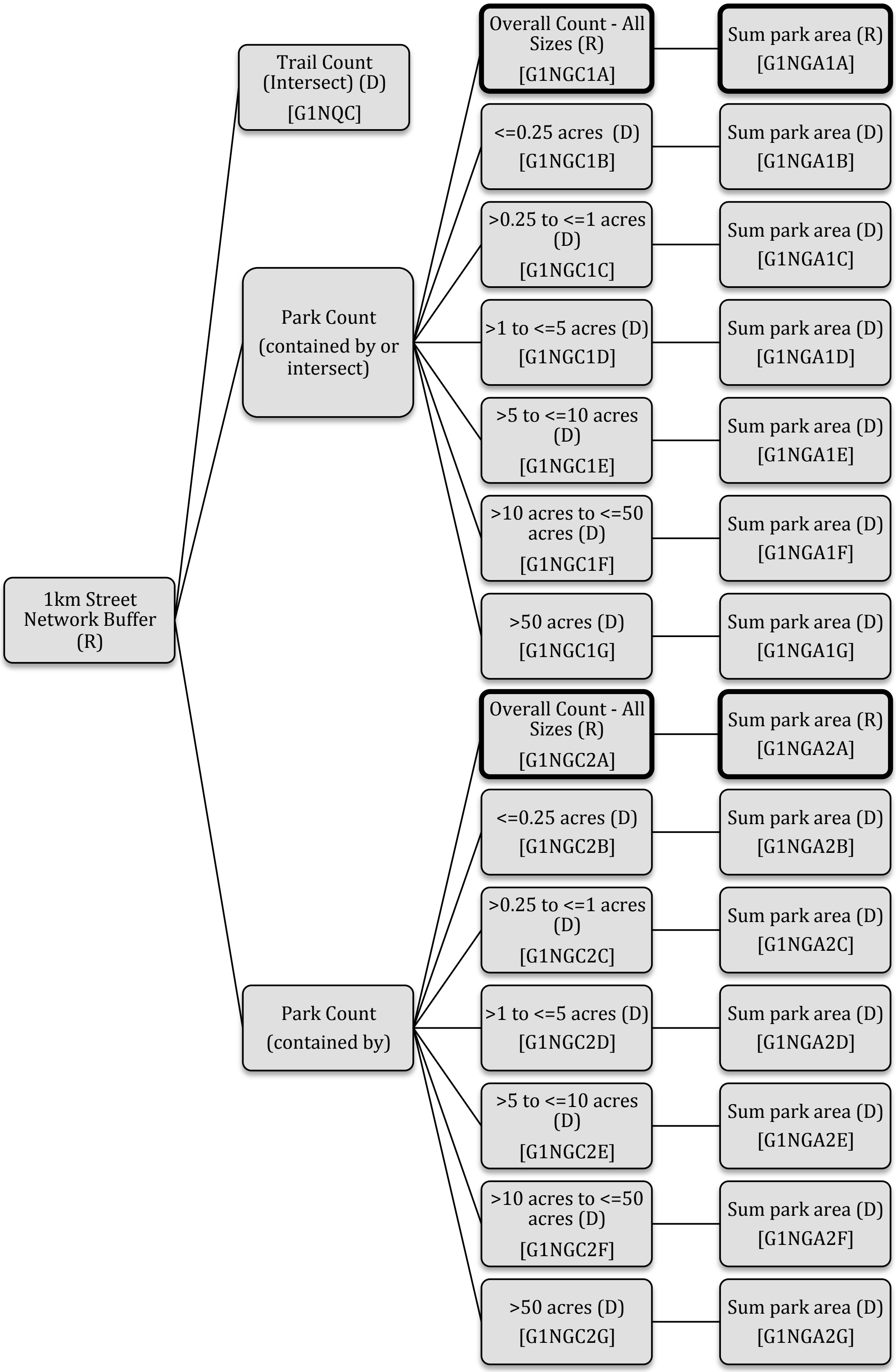


4/10/12

# SECTION 16

IPEN 500m Pedestrian Enhanced Street Network Buffers Variable Naming Convention

4/10/12

# IPEN: Residential Land Use

## 500m Pedestrian Enhanced Street Network Buffer

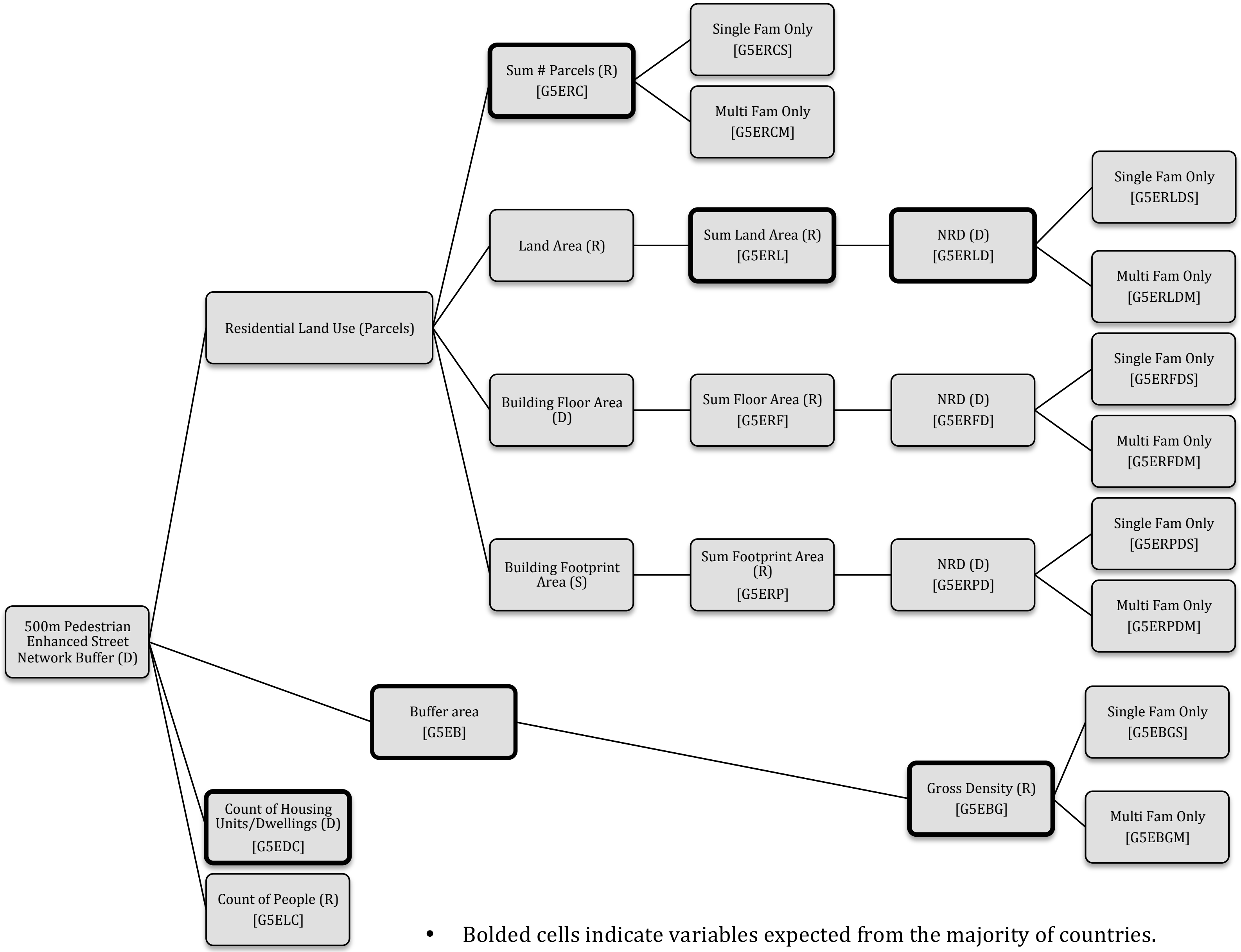


- Bolded cells indicate variables expected from the majority of countries.
- Letters in parentheses indicate whether variable is required (R), desired (D), or speculative (S).
- Alphanumeric values in [ ] provide variable names to use in GIS datafiles before [illegible]

4/10/12

# IPEN: Retail Land Use

## 500m Pedestrian Enhanced Street Network Buffer

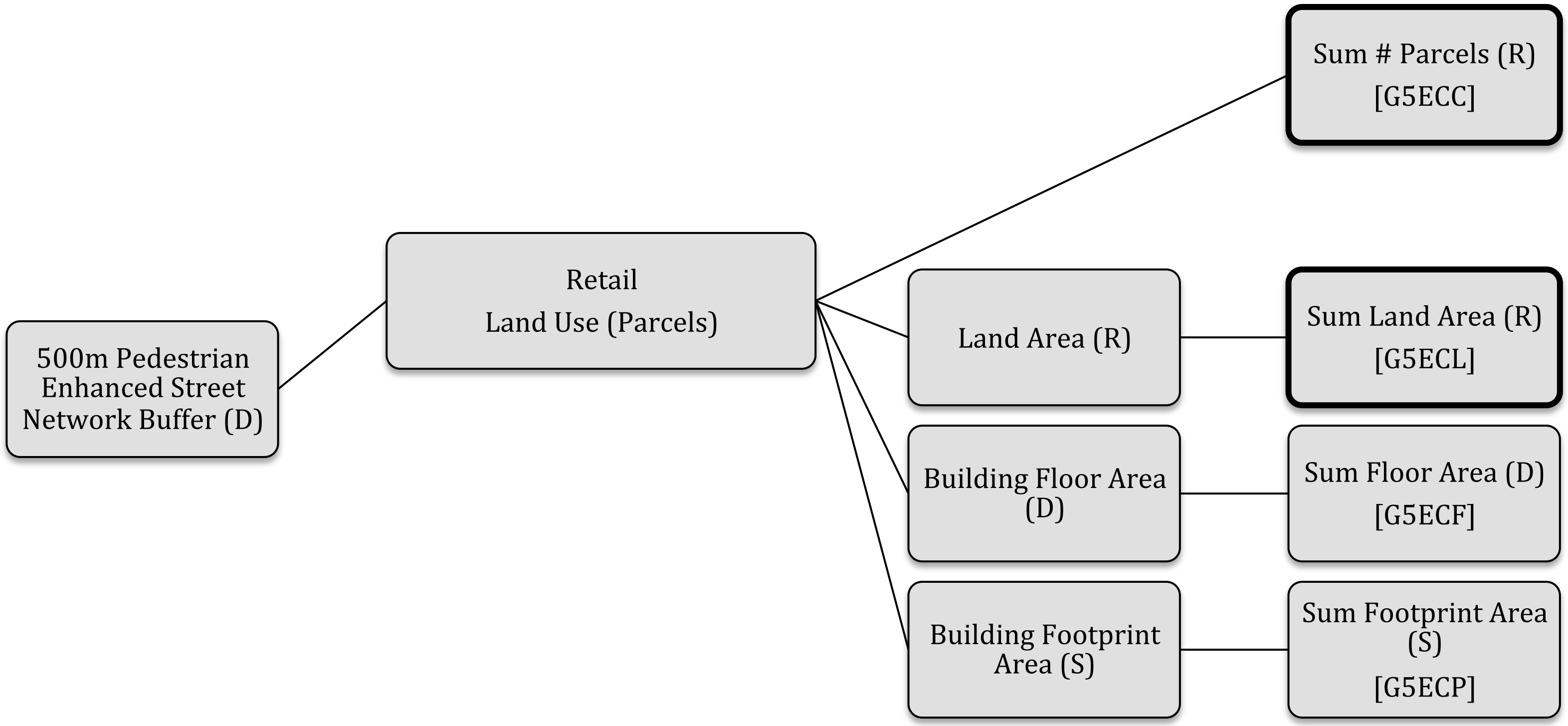


- Bolded cells indicate variables expected from the majority of countries.
- Letters in parentheses indicate whether variable is required (R) or desired (D).
- Alphanumeric values in [ ] provide variable names to use in GIS datafiles before sending to the coordinating center.

4/10/12

# IPEN: Civic and Institutional Land Use

## 500m Pedestrian Enhanced Street Network Buffer

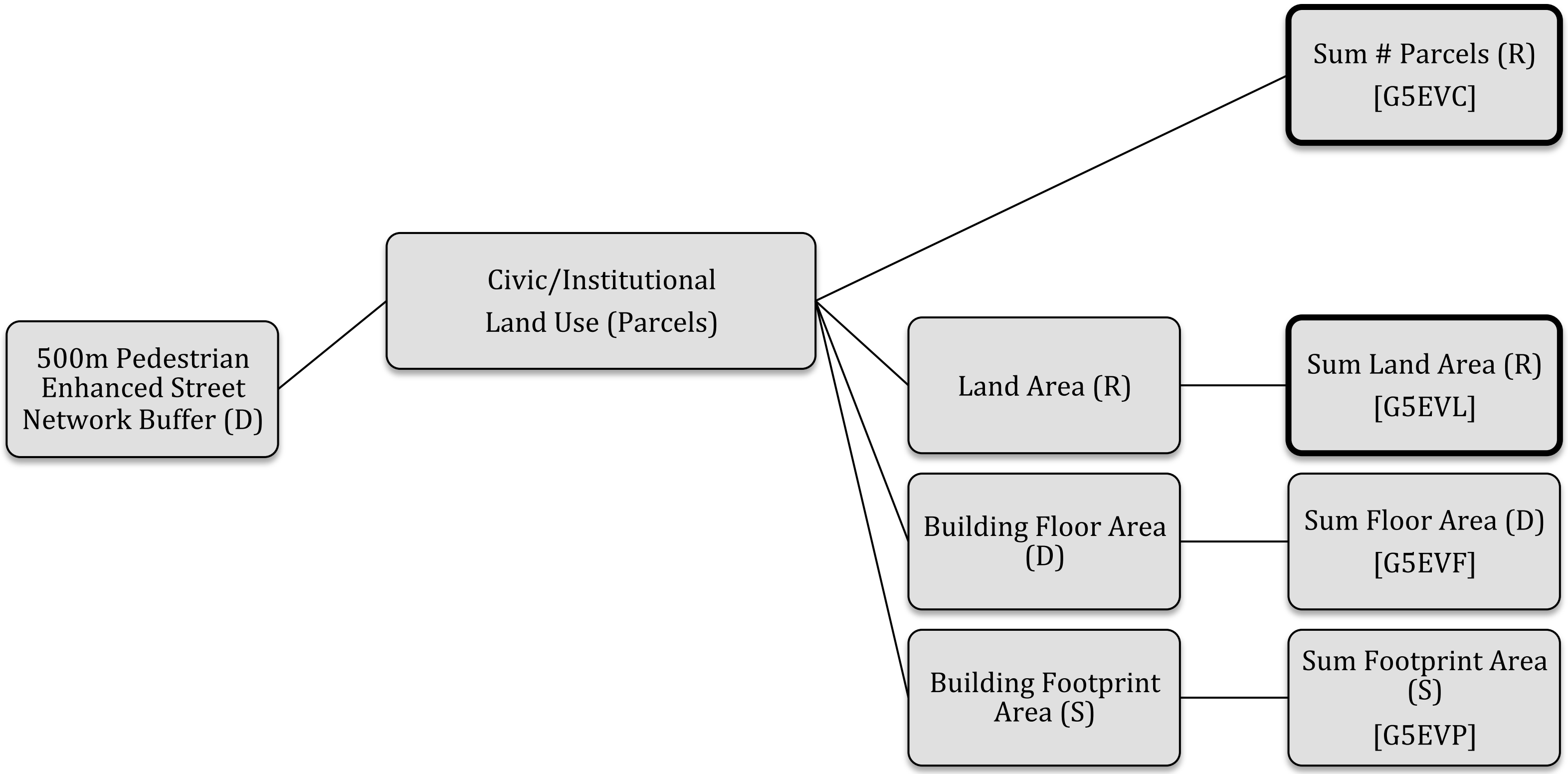


- Bolded cells indicate variables expected from the majority of countries.
- Letters in parentheses indicate whether variable is required (R) or desired (D).
- Alphanumeric values in [ ] provide variable names to use in GIS datafiles before sending to the coordinating center.

4/10/12

IPEN: Entertainment Land Use

500m Pedestrian Enhanced Street Network Buffer

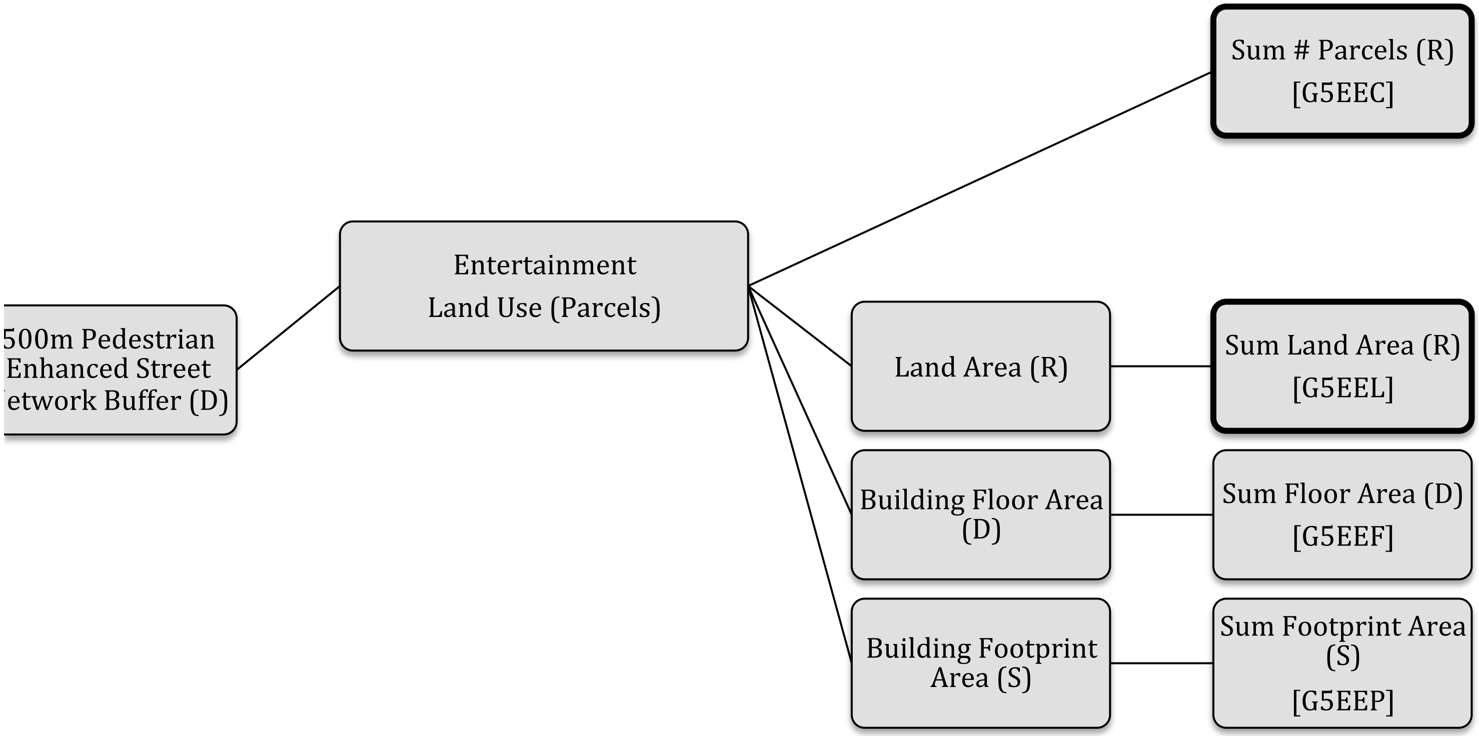


- Bolded cells indicate variables expected from the majority of countries.
- Letters in parentheses indicate whether variable is required (R) or desired (D).
- Alphanumeric values in [ ] provide variable names to use in GIS datafiles before sending to the coordinating center.

4/10/12

# IPEN: Private and Public Recreation Land Use

## 500m Pedestrian Enhanced Street Network Buffer

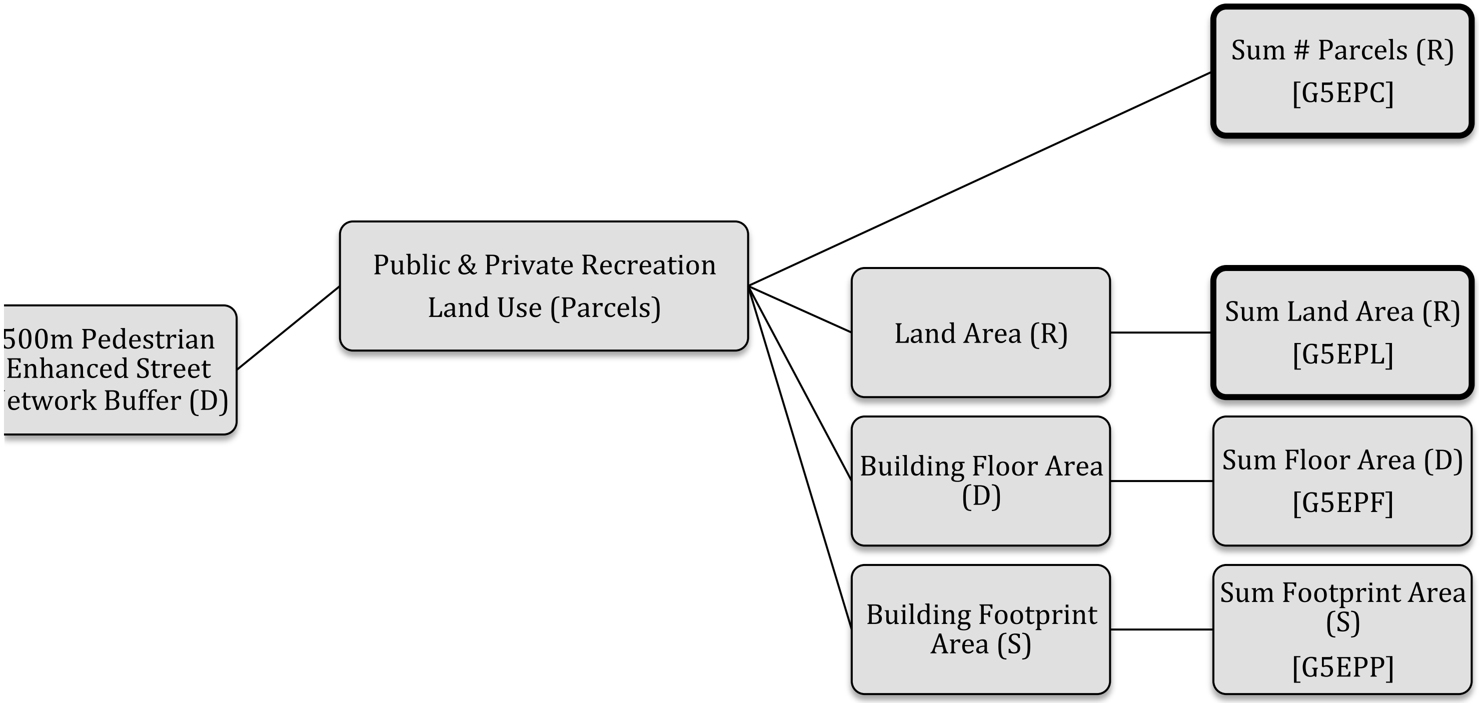


- Bolded cells indicate variables expected from the majority of countries.
- Letters in parentheses indicate whether variable is required (R) or desired (D).
- Alphanumeric values in [ ] provide variable names to use in GIS datafiles before sending to the coordinating center.

4/10/12

IPEN: Food and Restaurant Land Use

500m Pedestrian Enhanced Street Network Buffer

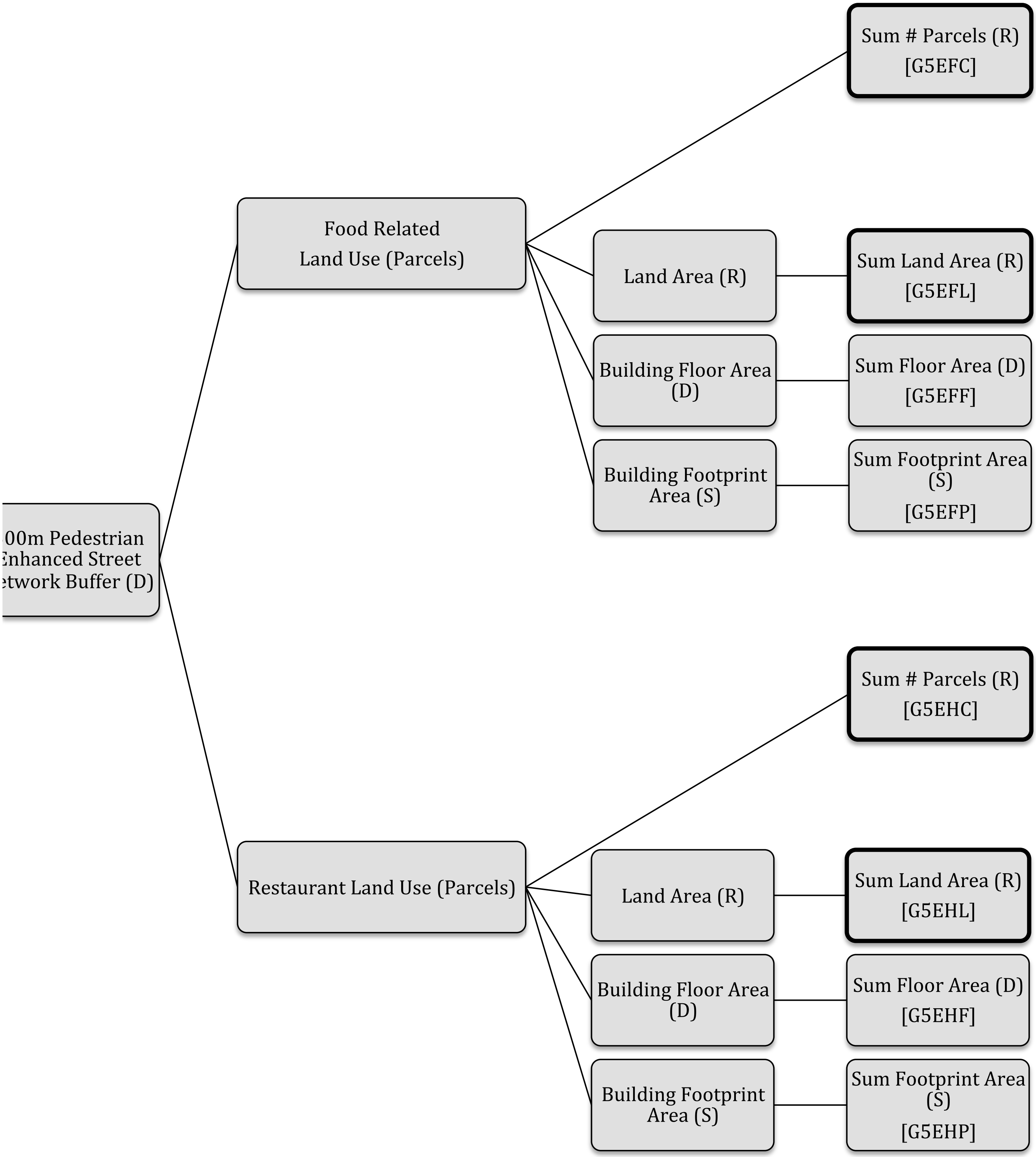


- Bolded cells indicate variables expected from the majority of countries.
- Letters in parentheses indicate whether variable is required (R) or desired (D).
- Alphanumeric values in [ ] provide variable names to use in GIS datafiles before sending to the coordinating center.

# IPEN: Intersections

## 500m Pedestrian Enhanced Street Network Buffer

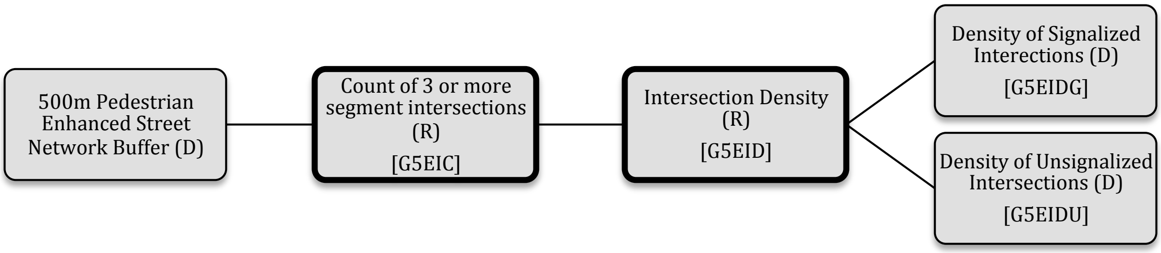


- Bolded cells indicate variables expected from the majority of countries.
- Letters in parentheses indicate whether variable is required (R) or desired (D).
- Alphanumeric values in [ ] provide variable names to use in GIS datafiles before sending to the coordinating center.

# IPEN: Public Transportation

## 500m Pedestrian Enhanced Street Network Buffer

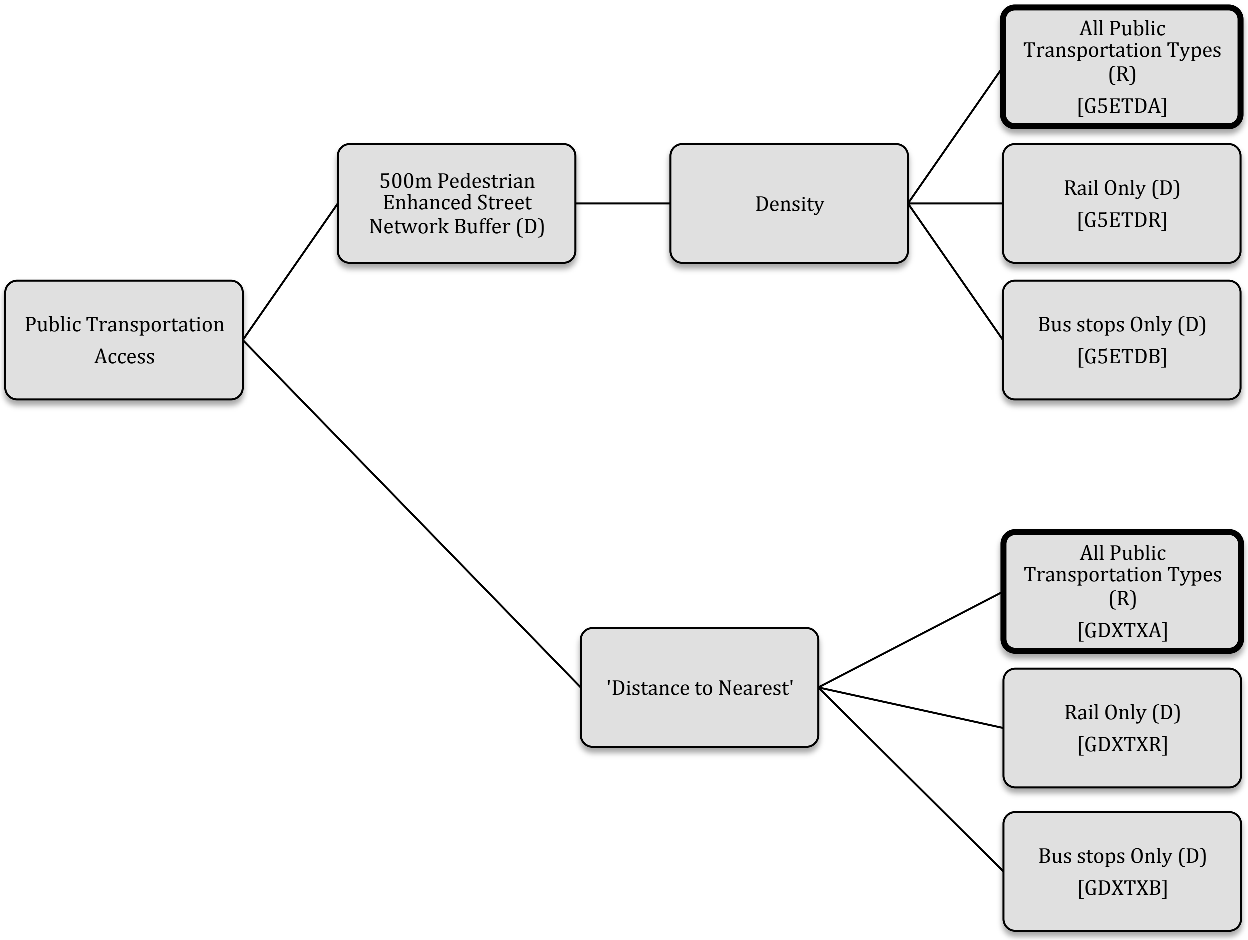


- Bolded cells indicate variables expected from the majority of countries.
- Letters in parentheses indicate whether variable is required (R) or desired (D).
- Alphanumeric values in [ ] provide variable names to use in GIS datafiles before sending to the coordinating center.

4/10/12

# IPEN:  Private Recreation Facility

## 500m Pedestrian Enhanced Street Network Buffer

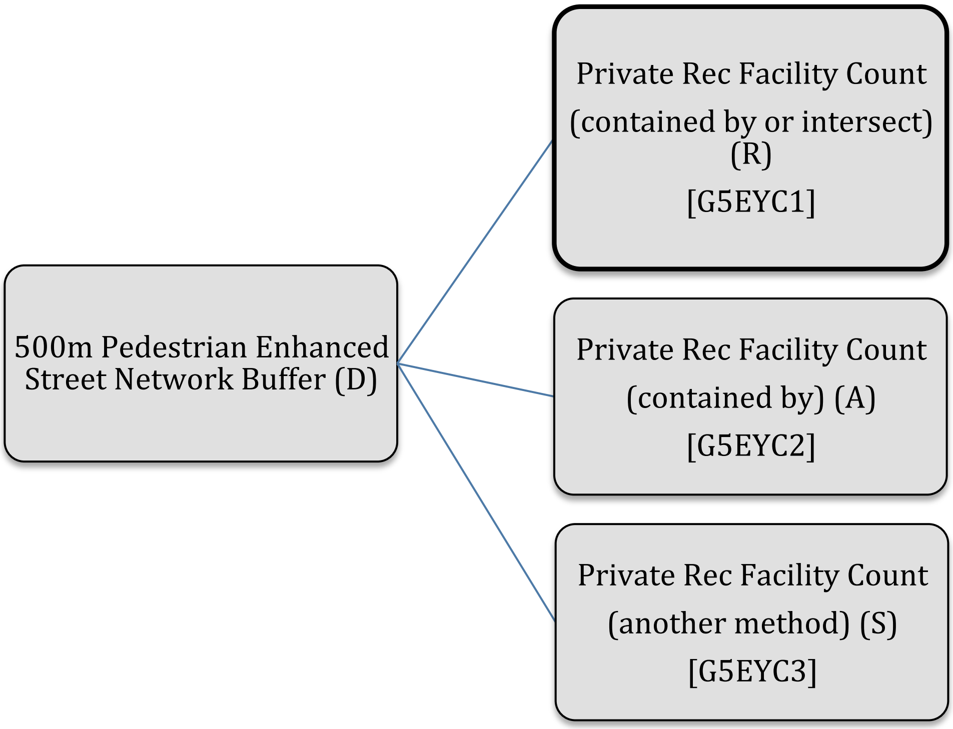


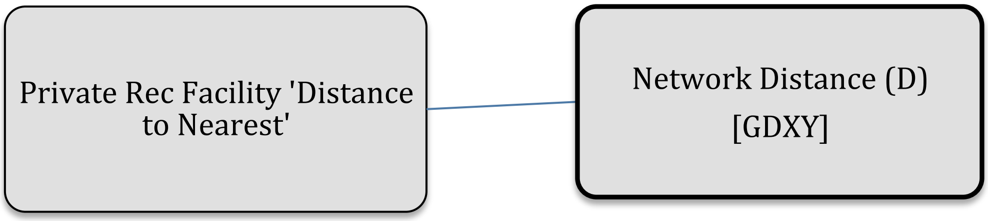


- Bolded cells indicate variables expected from the majority of countries.
- Letters in parentheses indicate whether variable is required (R) or desired (D).
- Alphanumeric values in [ ] provide variable names to use in GIS datafiles before sending to the coordinating center.

4/10/12

# SECTION 17

IPEN 1km Pedestrian Enhanced Street Network Buffers Variable Naming Convention

# IPEN: Residential Land Use

## 1km Pedestrian Enhanced Street Network Buffer

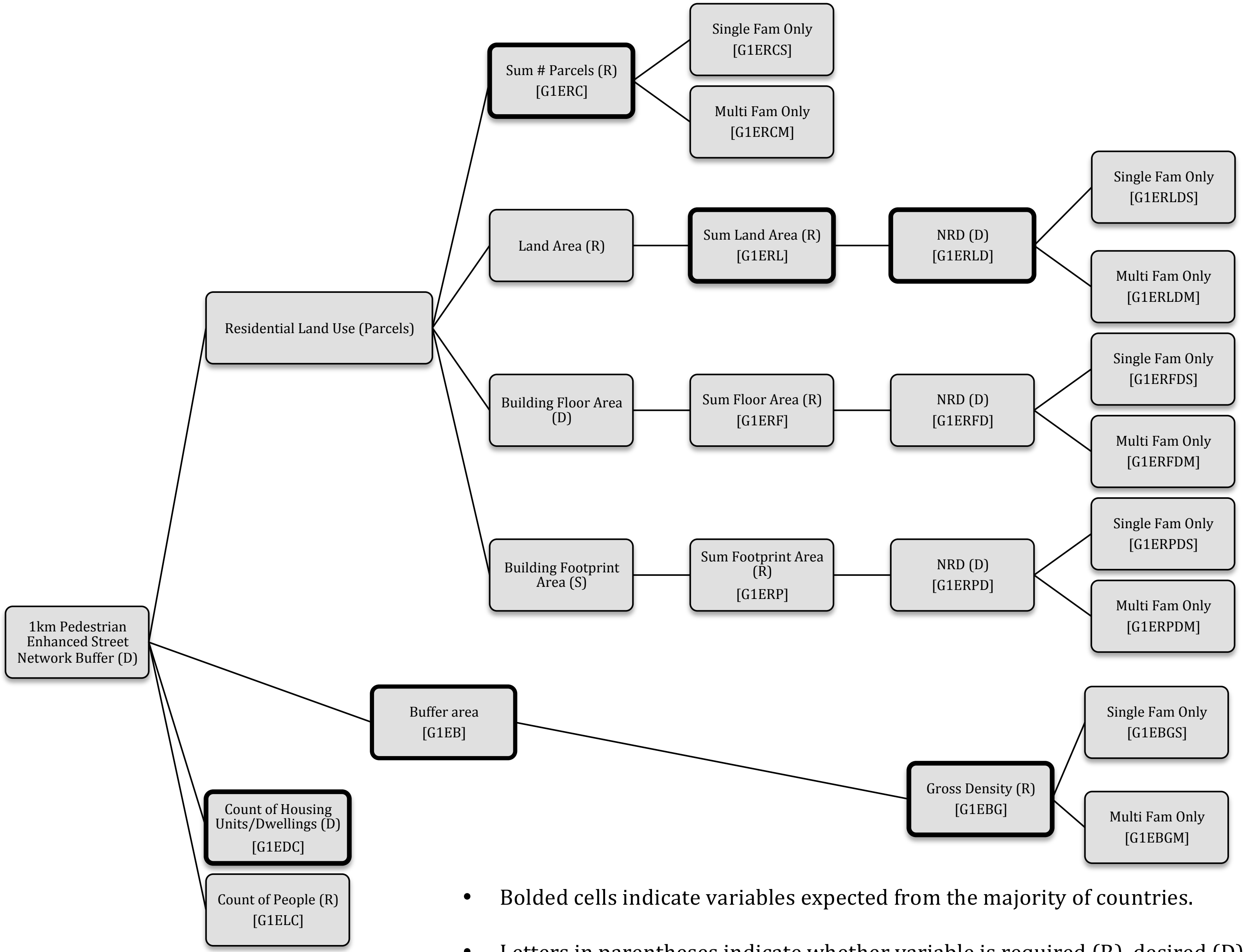


- Bolded cells indicate variables expected from the majority of countries.
- Letters in parentheses indicate whether variable is required (R), desired (D), or speculative (S).
- Alphanumeric values in [ ] provide variable names to use in GIS datafiles before sending to the coordinating center.

4/10/12

# IPEN: Retail Land Use

## 1km Pedestrian Enhanced Street Network Buffer

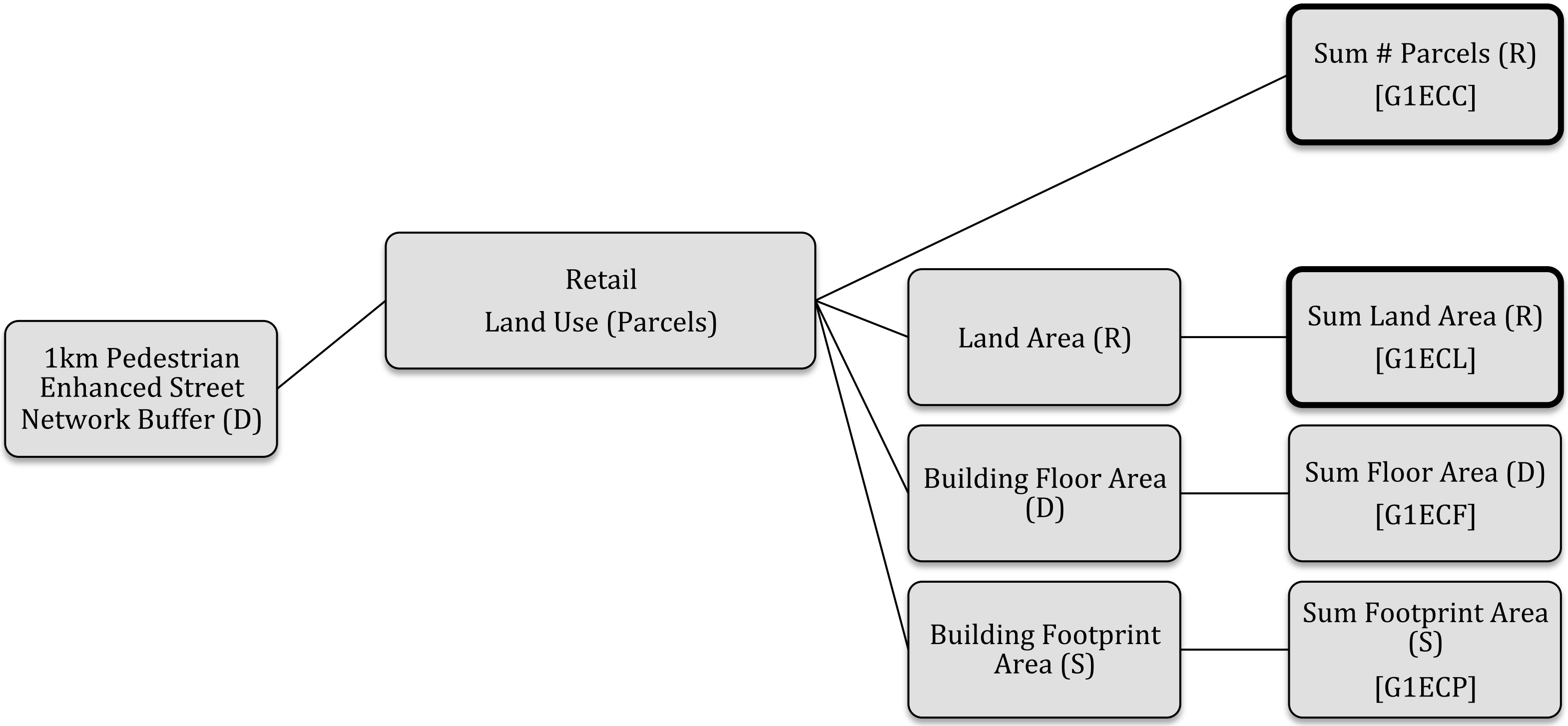


- Bolded cells indicate variables expected from the majority of countries.
- Letters in parentheses indicate whether variable is required (R) or desired (D).
- Alphanumeric values in [ ] provide variable names to use in GIS datafiles before sending to the coordinating center.

4/10/12

# IPEN: Civic and Institutional Land Use

## 1km Pedestrian Enhanced Street Network Buffer

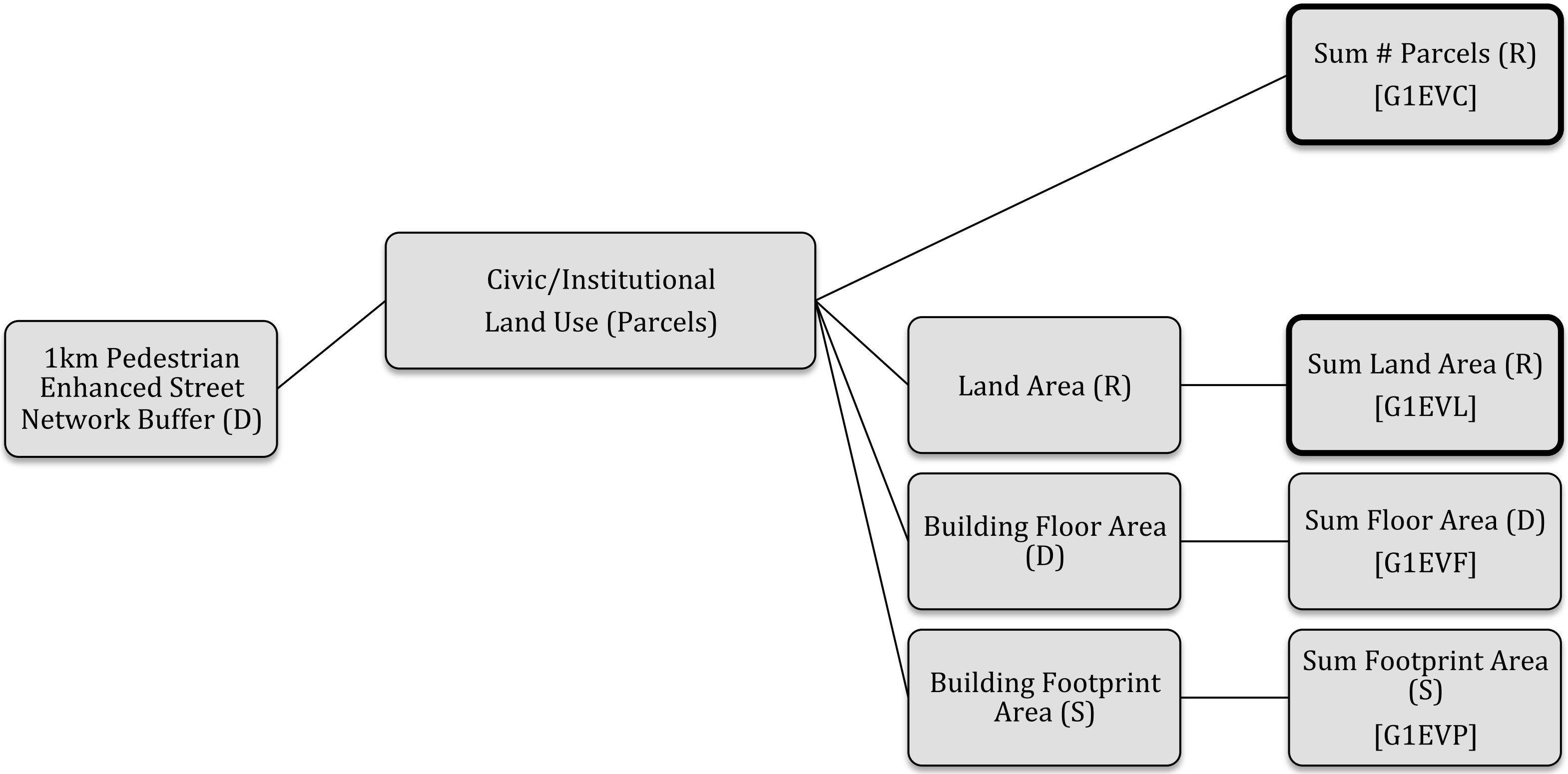


- Bolded cells indicate variables expected from the majority of countries.
- Letters in parentheses indicate whether variable is required (R) or desired (D).
- Alphanumeric values in [ ] provide variable names to use in GIS datafiles before sending to the coordinating center.

4/10/12

# IPEN: Entertainment Land Use

## 1km Pedestrian Enhanced Street Network Buffer

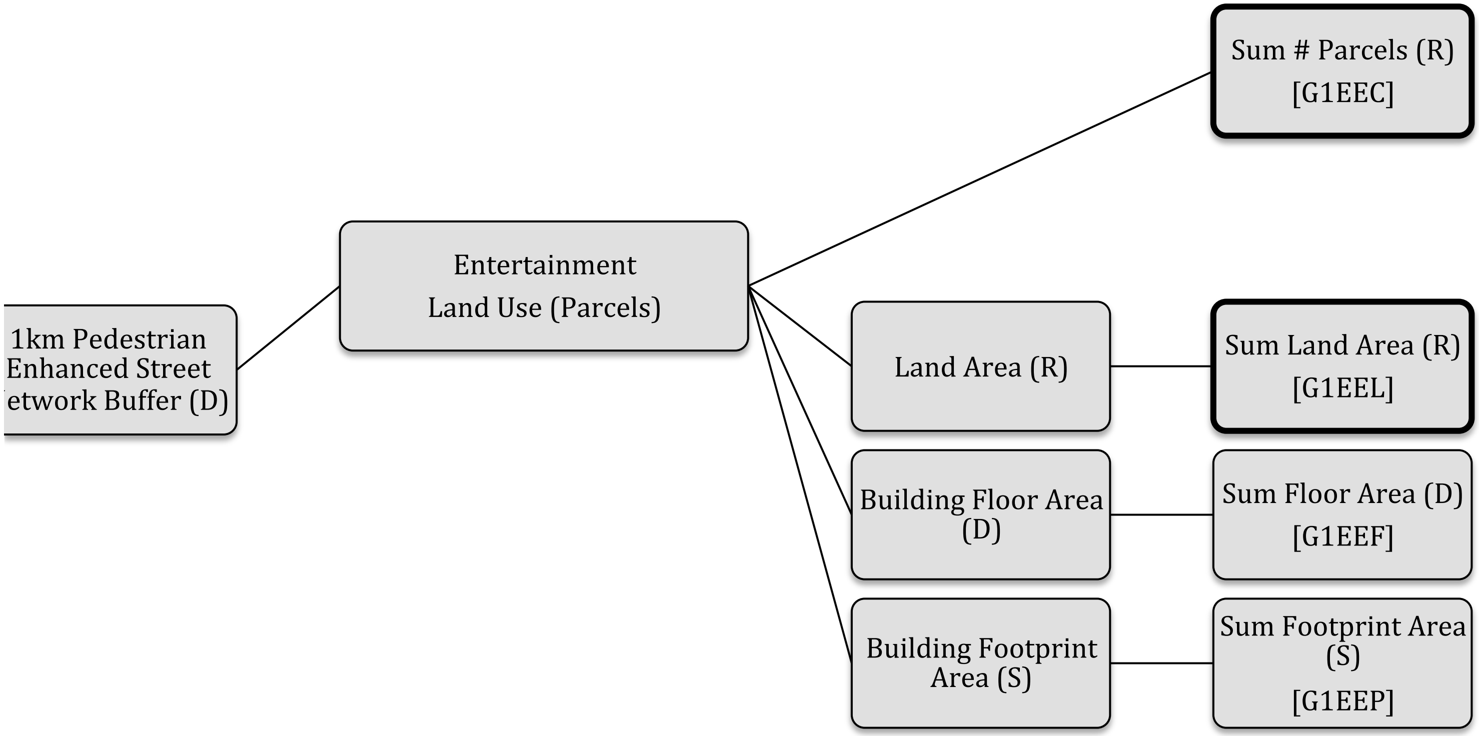


- Bolded cells indicate variables expected from the majority of countries.
- Letters in parentheses indicate whether variable is required (R) or desired (D).
- Alphanumeric values in [ ] provide variable names to use in GIS datafiles before sending to the coordinating center.

4/10/12

# IPEN: Private and Public Recreation Land Use

## 1km Pedestrian Enhanced Street Network Buffer

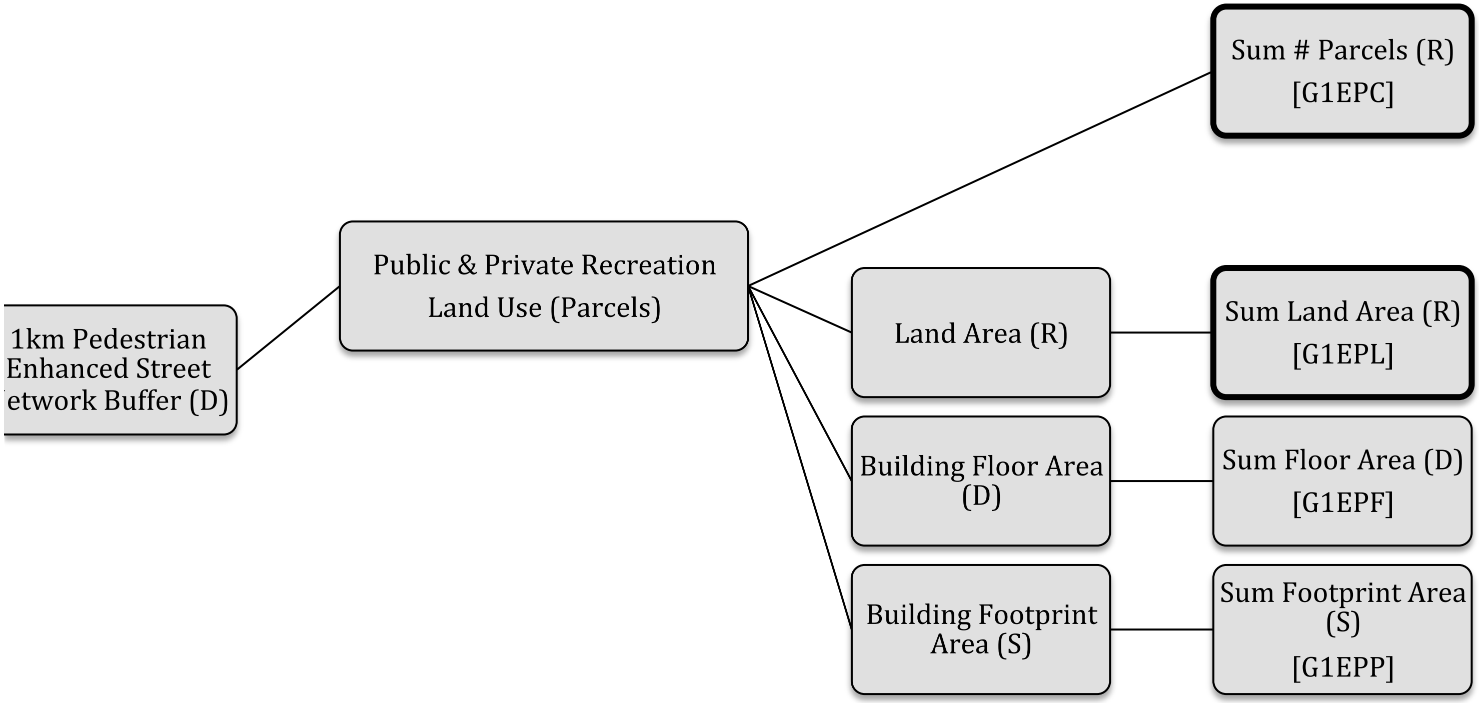


- Bolded cells indicate variables expected from the majority of countries.
- Letters in parentheses indicate whether variable is required (R) or desired (D).
- Alphanumeric values in [ ] provide variable names to use in GIS datafiles before sending to the coordinating center.

4/10/12

# IPEN: Food and Restaurant Land Use

## 1km Pedestrian Enhanced Street Network Buffer

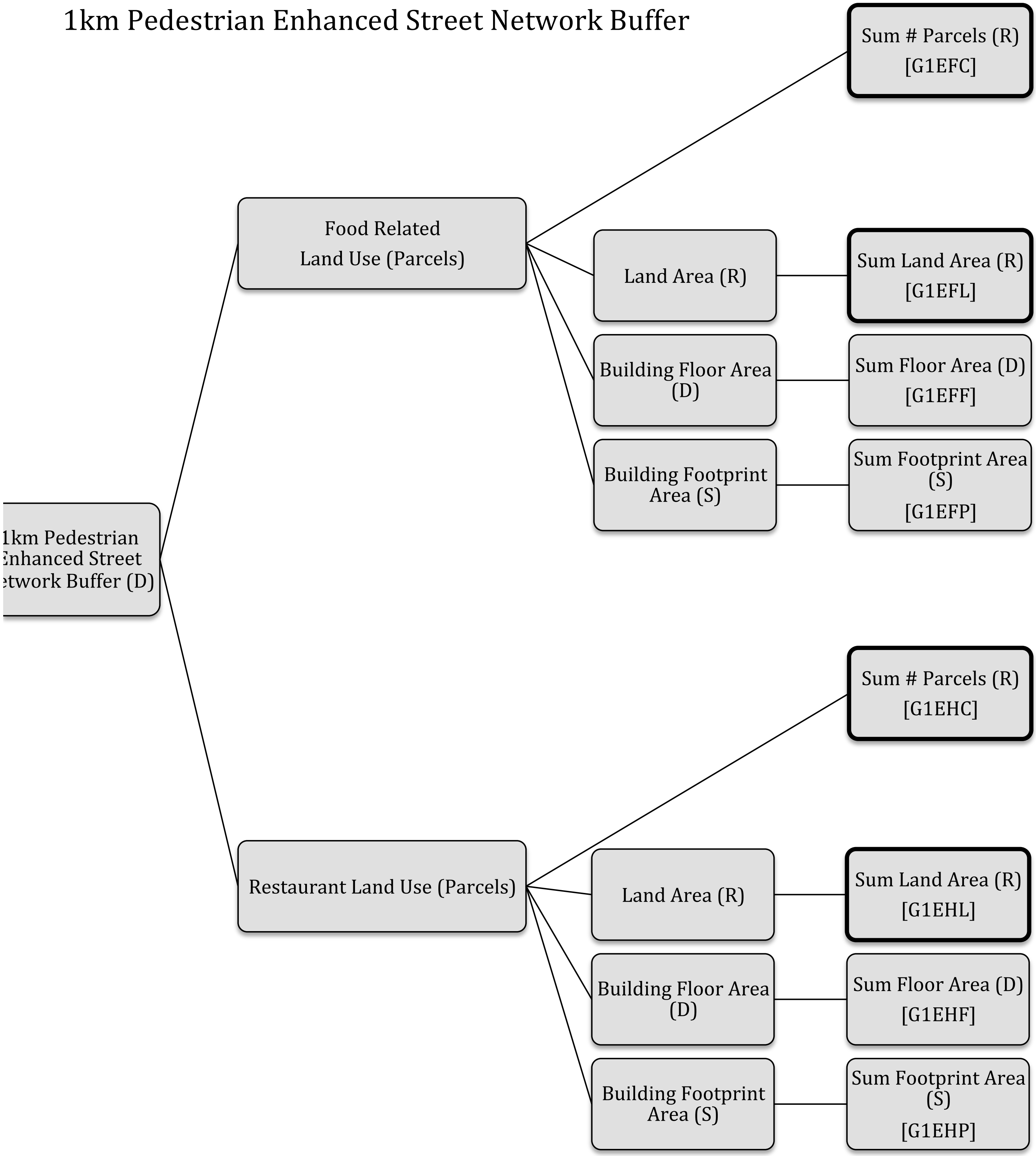


- Bolded cells indicate variables expected from the majority of countries.
- Letters in parentheses indicate whether variable is required (R) or desired (D).
- Alphanumeric values in [ ] provide variable names to use in GIS datafiles before sending to the coordinating center.

# IPEN: Street Connectivity

# 1km Pedestrian Enhanced Street Network Buffer

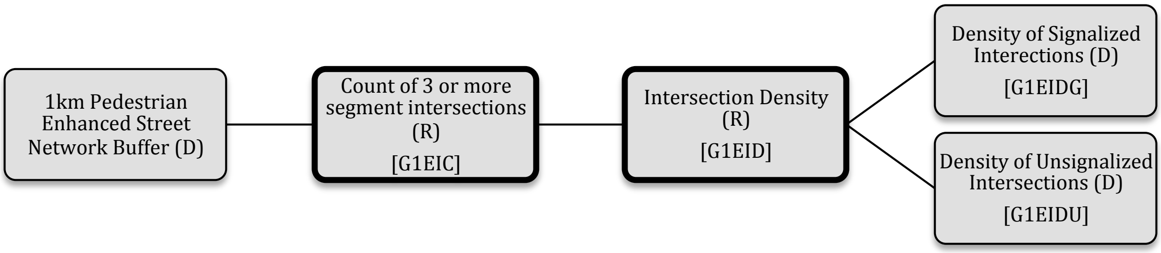


- Bolded cells indicate variables expected from the majority of countries.
- Letters in parentheses indicate whether variable is required (R) or desired (D).
- Alphanumeric values in [ ] provide variable names to use in GIS datafiles before sending to the coordinating center.

# IPEN: Public Transportation

## 1km Pedestrian Enhanced Street Network Buffer

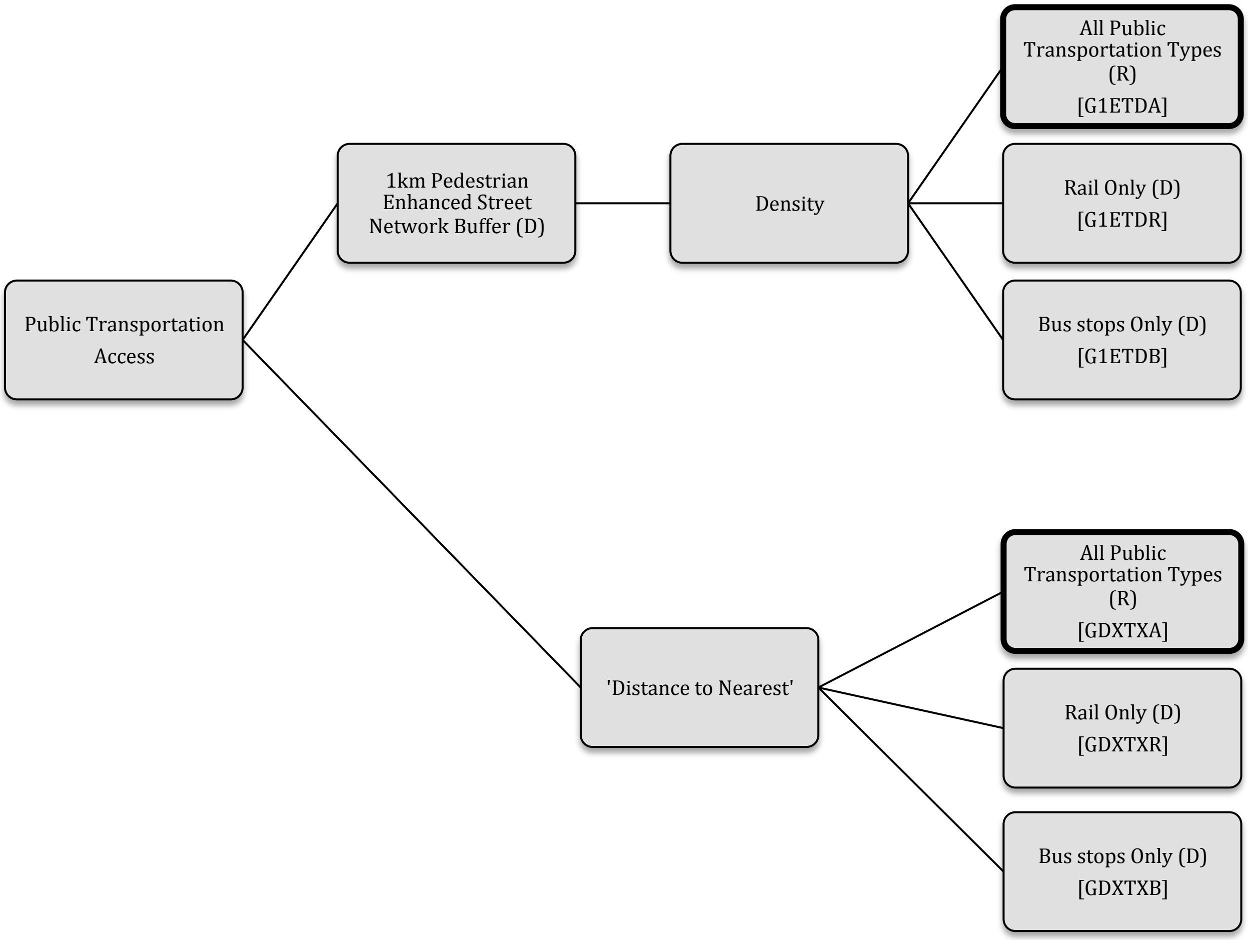


- Bolded cells indicate variables expected from the majority of countries.
- Letters in parentheses indicate whether variable is required (R) or desired (D).
- Alphanumeric values in [ ] provide variable names to use in GIS datafiles before sending to the coordinating center.

4/10/12

# IPEN: Private Recreation Facility

## 1km Pedestrian Enhanced Street Network Buffer

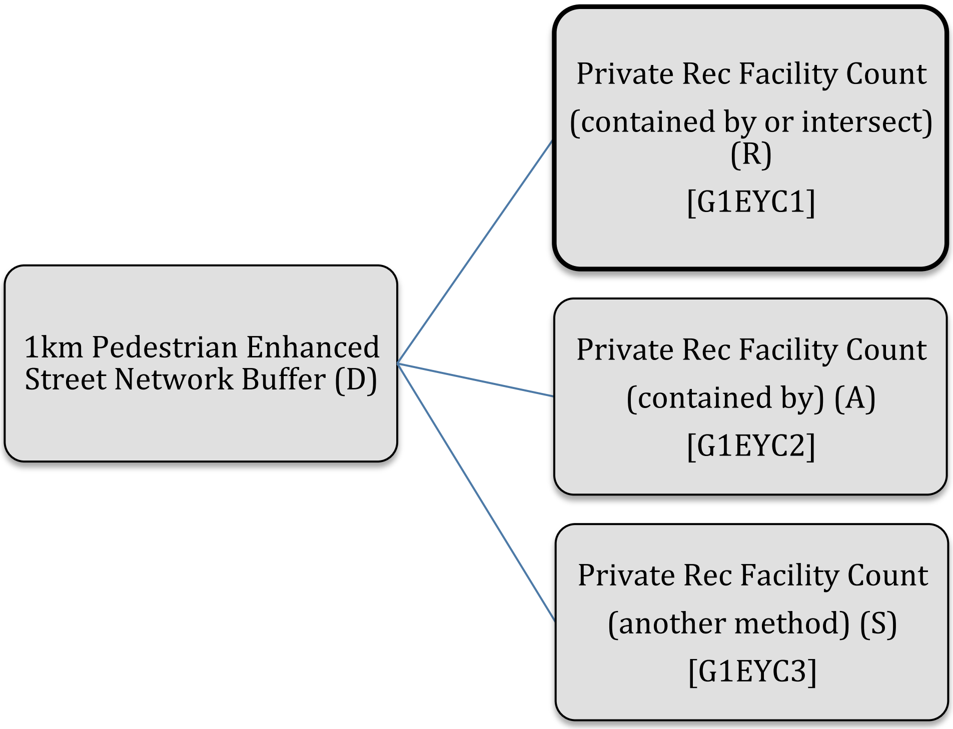


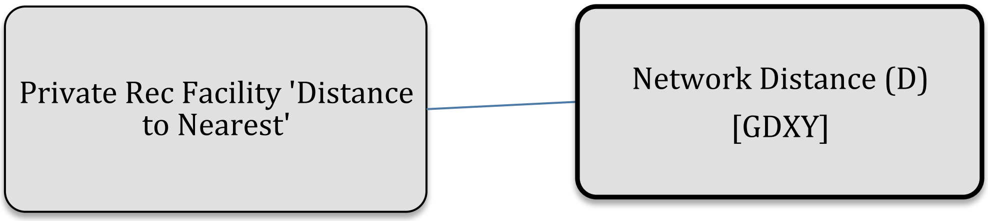


- Bolded cells indicate variables expected from the majority of countries.
- Letters in parentheses indicate whether variable is required (R) or desired (D).
- Alphanumeric values in [ ] provide variable names to use in GIS datafiles before sending to the coordinating center.

# IPEN:  Park and Trail Access Variables: Pedestrian Enhanced Street Network Buffer

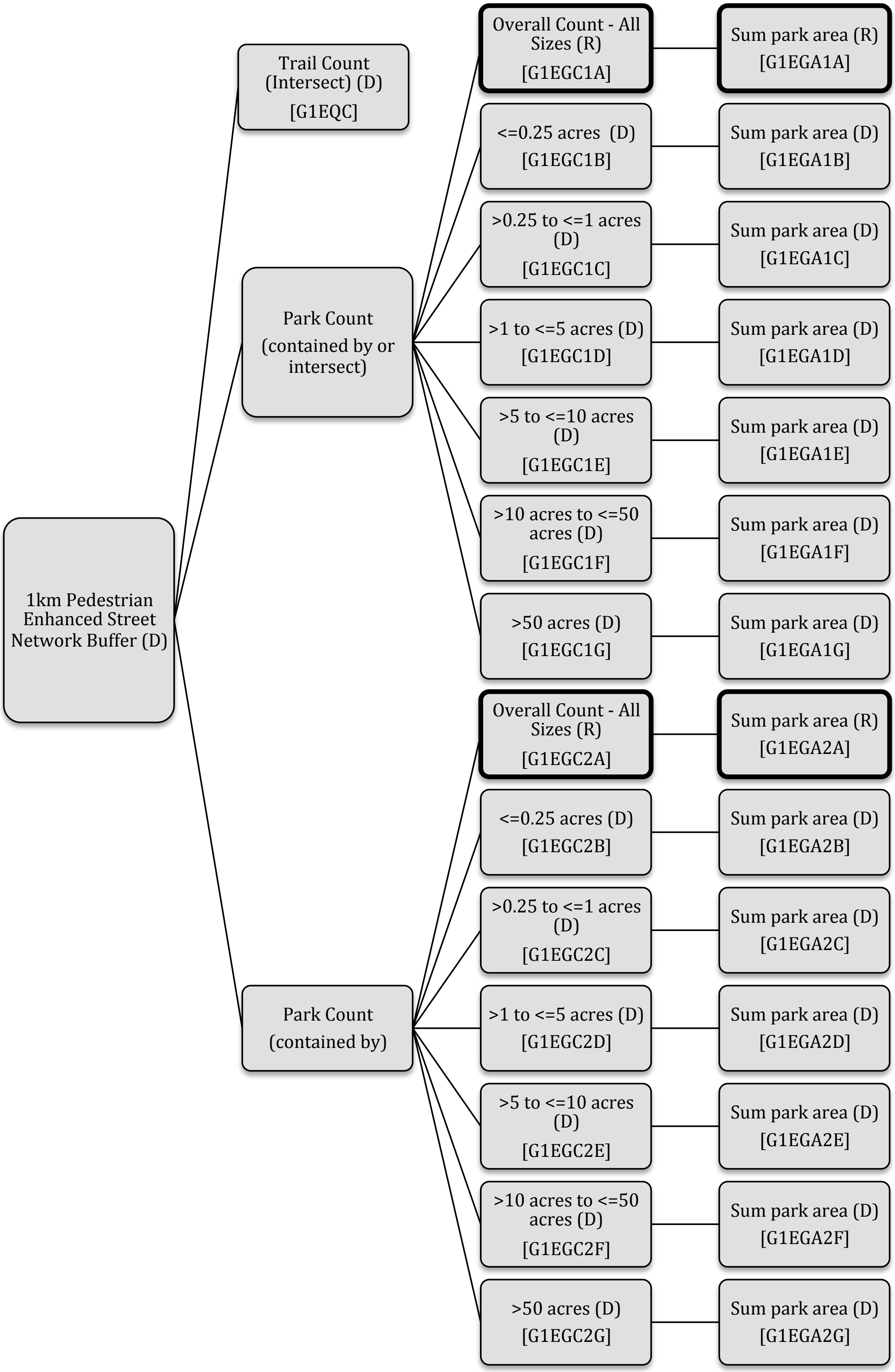

# SECTION 18: PARK DISTANCE VARIABLE NAMES

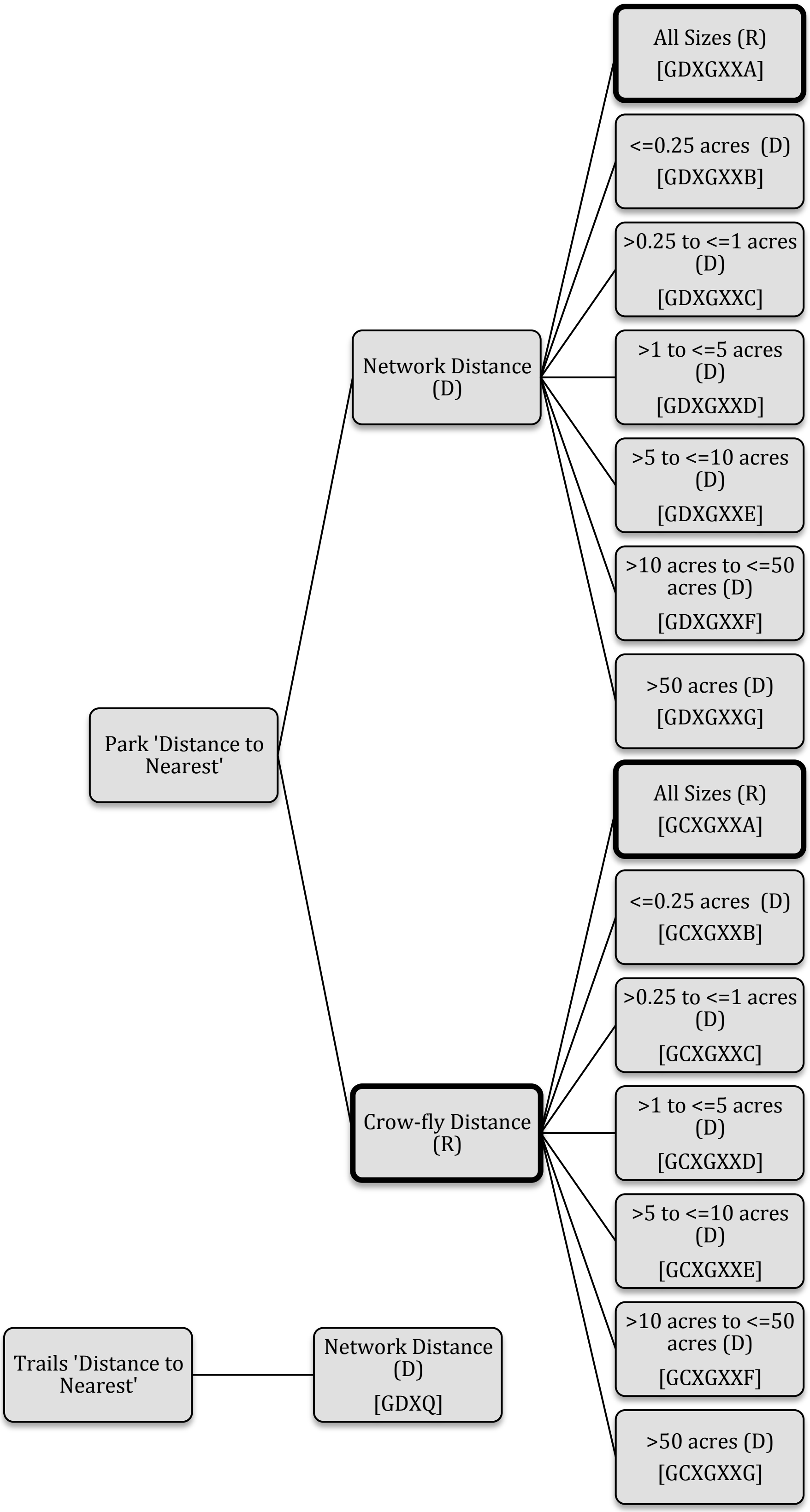

# SECTION 19: CHARACTER KEY FOR VARIABLE NAMES

| | |
|---|---|
| First character: | G = GIS variable |
| Second character: | 1 = 1km |
| | 5 = 500 meters |
| | D = Distance to nearest – network distance |
| | C = Distance to nearest – straight line distance |
| Third character: | N = Street network buffer |
| | E = Pedestrian enhanced street network buffer |
| | X = None |
| Fourth character: | B = Buffer |
| | C = Retail / Commercial |
| | D = Dwellings |
| | E = Entertainment |
| | F = Food |
| | G = Park |
| | H = Restaurant |
| | I = Intersection |
| | L = People |
| | P = Private and Public Recreation Land |
| | Q = Trail |
| | R = Residential |
| | T = Transportation |
| | V = Civic / Institutional |
| | Y = Private Recreation Facilities |
| Fifth character: | A = Area |

C = Count

D = Density

G = Gross Density

F = Floor Area Sum

L = Land Area Sum

P = Footprint Area Sum

X = None

Sixth character: 1 = Contained by or intersect

2 = Contained by

3 = Another method

A = Overall

B = Bus Only

D = Net Density

G = Signalized

M = Multiple Family

R = Rail Only

S = Single Family

U = Unsignalized

X = None

Seventh character: A = Overall

B = less than or equal to .25 acre

C = greater than .25 acre but less than or equal to 1 acre

D = Greater than 1 acre but less than or equal to 5 acres

E = Greater than 5 acres but less than or equal to 10 acres

F = Greater than 10 acres but less than or equal to 50 acres

G = Greater than 50 acres

M = Multiple Family

S = Single Family
