# Supplementary material for: International variation in neighborhood walkability, transit, and recreation environments using geographic information systems: the IPEN adult study
Source: Int J Health Geogr. 2014 Oct 25;13:43. doi: 10.1186/1476-072X-13-43 (PMC4221715; doi:10.1186/1476-072X-13-43)
Supplement: Supplementary file 6 — Additional file 6: “Intersection densities for participants’ 500-m network buffers across cities and countries”. (PDF 61 KB) [file 12942_2014_609_MOESM6_ESM.pdf]

Additional file 6: Intersection densities for participants' 500m network buffers across cities and countries.

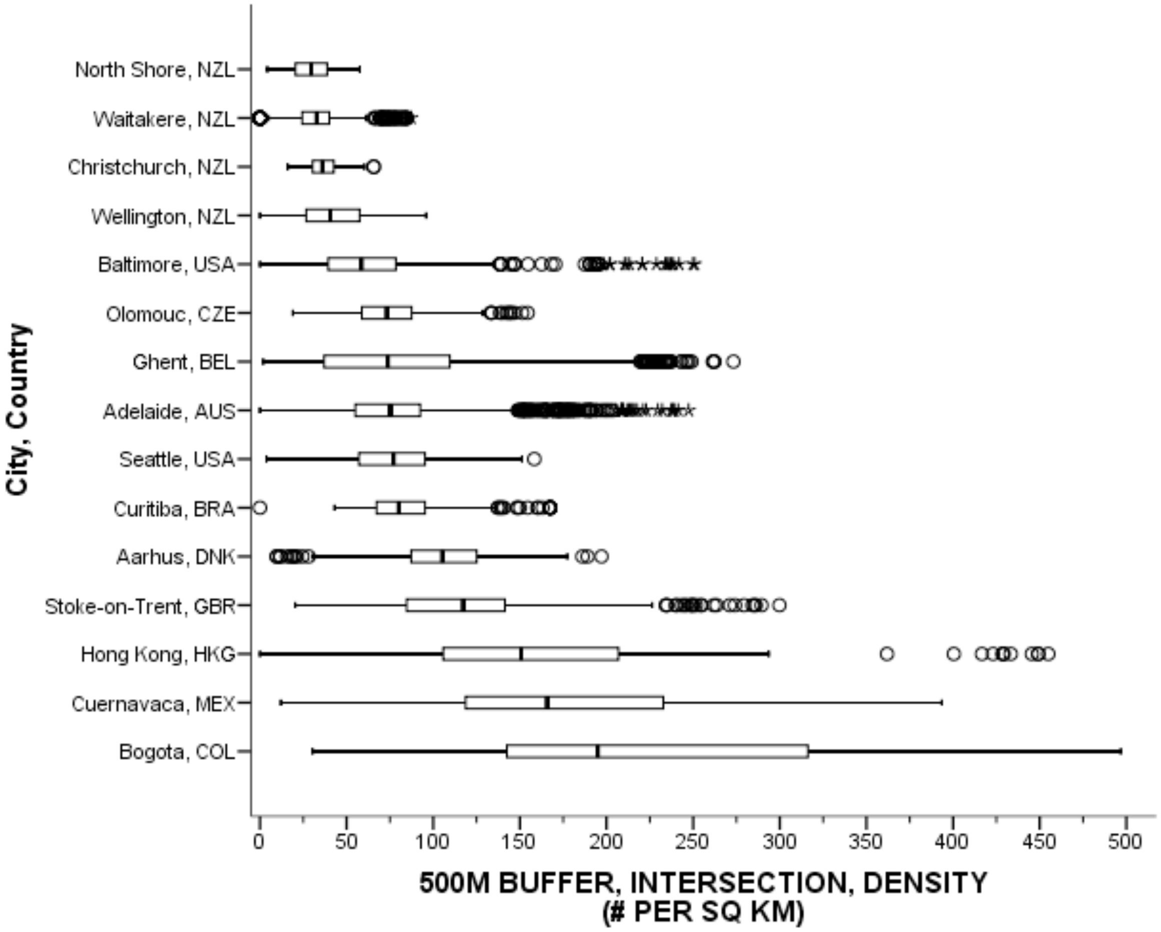

Circles are outliers that extend past the whiskers and asterisks represent extreme outliers defined as values greater than three times the length of the interquartile range.
