# Supplementary material for: International variation in neighborhood walkability, transit, and recreation environments using geographic information systems: the IPEN adult study
Source: Int J Health Geogr. 2014 Oct 25;13:43. doi: 10.1186/1476-072X-13-43 (PMC4221715; doi:10.1186/1476-072X-13-43)
Supplement: Supplementary file 7 — Additional file 7: “Walkability scores across cities and countries within participants’ 500-m network buffer”. (PDF 60 KB) [file 12942_2014_609_MOESM7_ESM.pdf]

Additional file 7: Walkability scores across cities and countries within participants' 500m network buffer.

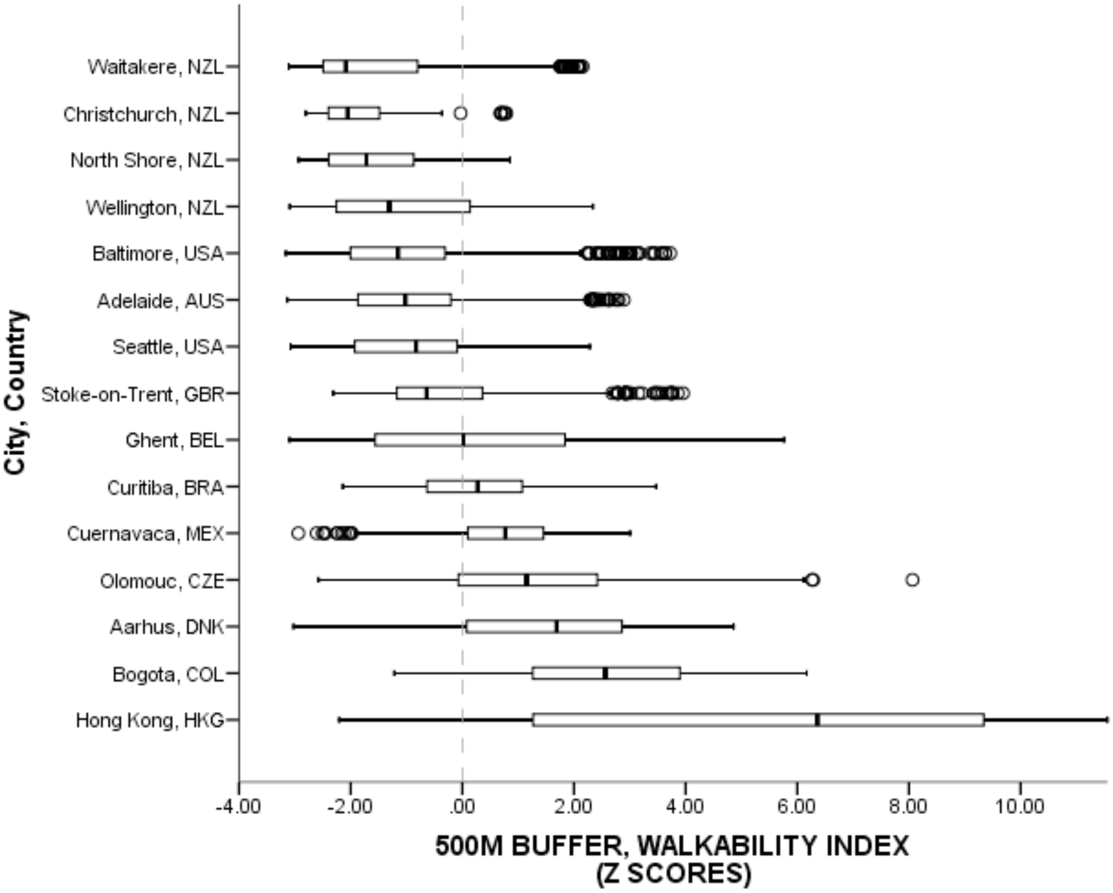

[1]Circles are outliers that extend past the whiskers and asterisks represent extreme outliers defined as values greater than three times the length of the interquartile range. [2]Walkability z-score equaled the sum of z-scores for residential density, land use mix, and intersection density. Z-scores allowed for standardized pooled standard deviations necessary for comparisons across countries.
