# Supplementary material for: International variation in neighborhood walkability, transit, and recreation environments using geographic information systems: the IPEN adult study
Source: Int J Health Geogr. 2014 Oct 25;13:43. doi: 10.1186/1476-072X-13-43 (PMC4221715; doi:10.1186/1476-072X-13-43)
Supplement: Supplementary file 10 — Additional file 10: “Density of recreation facilities for participants’ 500-m network buffers across cities and countries”. (PDF 57 KB) [file 12942_2014_609_MOESM10_ESM.pdf]

Additional file 10: Density of private recreation facilities for participants' 500m network buffers across cities and countries.

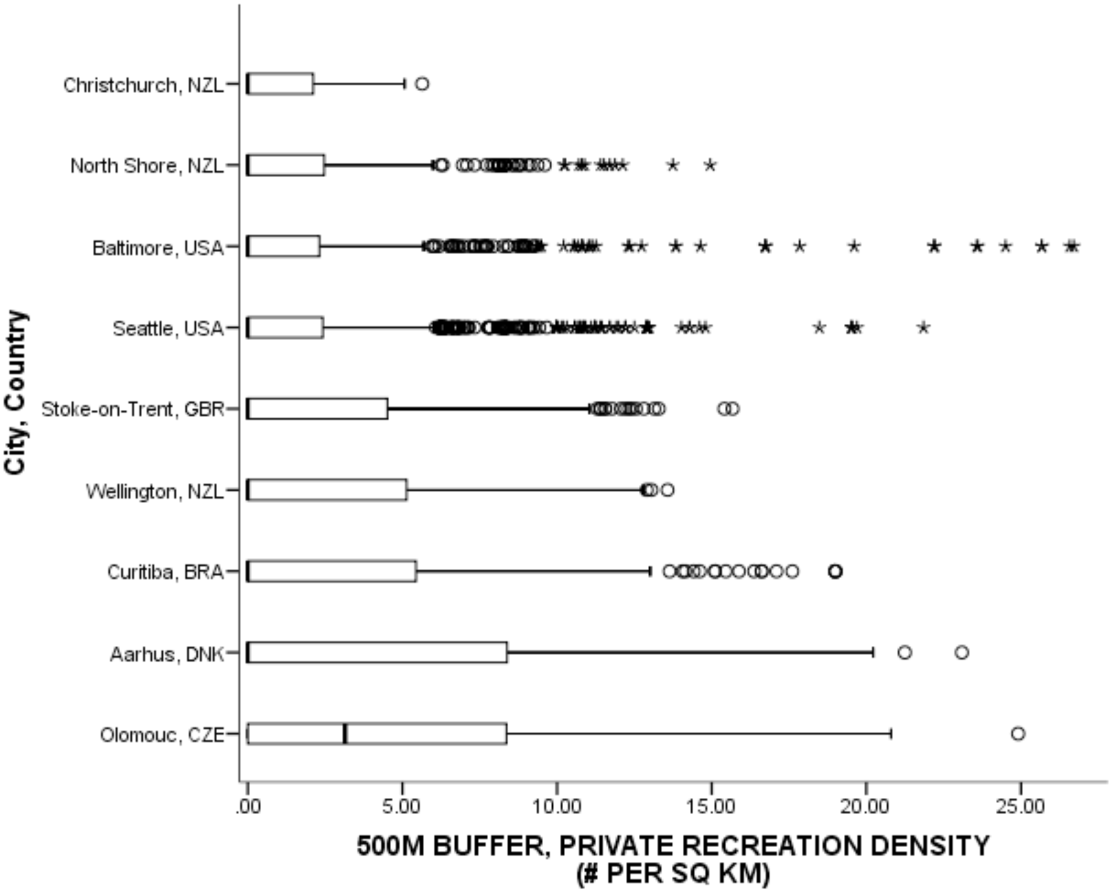

Circles are outliers that extend past the whiskers and asterisks represent extreme outliers defined as values greater than three times the length of the interquartile range.
